# Genetic variant predictors of gene expression provide new insight into risk of colorectal cancer

**DOI:** 10.1007/s00439-019-01989-8

**Published:** 2019-02-28

**Authors:** Stephanie A. Bien, Yu-Ru Su, David V. Conti, Tabitha A. Harrison, Conghui Qu, Xingyi Guo, Yingchang Lu, Demetrius Albanes, Paul L. Auer, Barbara L. Banbury, Sonja I. Berndt, Stéphane Bézieau, Hermann Brenner, Daniel D. Buchanan, Bette J. Caan, Peter T. Campbell, Christopher S. Carlson, Andrew T. Chan, Jenny Chang-Claude, Sai Chen, Charles M. Connolly, Douglas F. Easton, Edith J. M. Feskens, Steven Gallinger, Graham G. Giles, Marc J. Gunter, Jochen Hampe, Jeroen R. Huyghe, Michael Hoffmeister, Thomas J. Hudson, Eric J. Jacobs, Mark A. Jenkins, Ellen Kampman, Hyun Min Kang, Tilman Kühn, Sébastien Küry, Flavio Lejbkowicz, Loic Le Marchand, Roger L. Milne, Li Li, Christopher I. Li, Annika Lindblom, Noralane M. Lindor, Vicente Martín, Caroline E. McNeil, Marilena Melas, Victor Moreno, Polly A. Newcomb, Kenneth Offit, Paul D. P. Pharaoh, John D. Potter, Chenxu Qu, Elio Riboli, Gad Rennert, Núria Sala, Clemens Schafmayer, Peter C. Scacheri, Stephanie L. Schmit, Gianluca Severi, Martha L. Slattery, Joshua D. Smith, Antonia Trichopoulou, Rosario Tumino, Cornelia M. Ulrich, Fränzel J. B. van Duijnhoven, Bethany Van Guelpen, Stephanie J. Weinstein, Emily White, Alicja Wolk, Michael O. Woods, Anna H. Wu, Goncalo R. Abecasis, Graham Casey, Deborah A. Nickerson, Stephen B. Gruber, Li Hsu, Wei Zheng, Ulrike Peters

**Affiliations:** 1 Division of Public Health Sciences, Fred Hutchinson Cancer Research Center, Seattle, WA 98109 USA; 2 USC Norris Comprehensive Cancer Center, University of Southern California, Los Angeles, CA 90089 USA; 3 Department of Preventive Medicine, Keck School of Medicine, University of Southern California, Los Angeles, CA 90033 USA; 4 Division of Epidemiology, Vanderbilt University School of Medicine, Nashville, TN 37232 USA; 5 Division of Cancer Epidemiology and Genetics, National Cancer Institute, Bethesda, MD 20892 USA; 6 Joseph J. Zilber School of Public Health, University of Wisconsin-Milwaukee, Milwaukee, WI 53205 USA; 7 Centre Hospitalier Universitaire Hotel-Dieu, 44093 Nantes, France; 8 Service de Génétique Médiczle, Centre Hospitalier Universitaire (CHU), 44093 Nantes, France; 9 Division of Clinical Epidemiology and Aging Research, German Cancer Research Center (DKFZ), 69120 Heidelberg, Germany; 10 Division of Preventive Oncology, German Cancer Research Center (DKFZ) and National Center for Tumor Diseases (NCT), 69120 Heidelberg, Germany; 11 German Cancer Consortium (DKTK), 69120 Heidelberg, Germany; 12 Centre for Epidemiology and Biostatistics, Melbourne School of Population and Global Health, The University of Melbourne, Parkville, VIC 3010 Australia; 13 Colorectal Oncogenomics Group, Department of Pathology, University of Melbourne, Melbourne, VIC 3010 Australia; 14 Genetic Medicine and Familial Cancer Centre, The Royal Melbourne Hospital, Parkville, VIC 3010 Australia; 15 Division of Research, Kaiser Permanente Medical Care Program of Northern California, Oakland, CA 94612 USA; 16 Epidemiology Research Program, American Cancer Society, Atlanta, GA 30329-4251 USA; 17 Division of Gastroenterology, Massachusetts General Hospital, Harvard Medical School, Boston, MA 02114 USA; 18 Channing Division of Network Medicine, Brigham and Women’s Hospital and Harvard Medical School, Boston, MA 02115 USA; 19 Unit of Genetic Epidemiology, Division of Cancer Epidemiology, German Cancer Research Center (DKFZ), 69120 Heidelberg, Germany; 20 Genetic Tumour Epidemiology Group, University Medical Center Hamburg-Eppendorf, University Cancer Center Hamburg, 20246 Hamburg, Germany; 21 Department of Biostatistics, University of Michigan, Ann Arbor, MI 48109 USA; 22 Department of Public Health and Primary Care School of Clinical Medicine, University of Cambridge, Cambridge, England 01223 UK; 23 Division of Human Nutrition, Wageningen University & Research, Wageningen, The Netherlands; 24 Lunenfeld Tanenbaum Research Institute, Mount Sinai Hospital, University of Toronto, Toronto, ON 1X5 Canada; 25 Cancer Epidemiology & Intelligence Division, Cancer Council Victoria, Melbourne, 3004 Australia; 26 Section for Epidemiology, Department of Public Health, Aarhus University, Aarhus, Denmark; 27 Medical Department 1, University Hospital Dresden, TU Dresden, 01307 Dresden, Germany; 28 Ontario Institute for Cancer Research, Toronto, ON Canada; 29 AbbVie Inc, 1500 Seaport Blvd, Redwood City, CA 94063 USA; 30 Division of Cancer Epidemiology, German Cancer Research Center (DKFZ), Heidelberg, Germany; 31 Clalit Health Services National Israeli Cancer Control Center, 34361 Haifa, Israel; 32 Department of Community Medicine and Epidemiology, Carmel Medical Center, 34361 Haifa, Israel; 33 University of Hawai’i Cancer Center, Honolulu, Hawaii 96813 USA; 34 Department of Family Medicine and Community Health, Case Western Reserve University, Cleveland, OH 44106 USA; 35 Department of Clinical Genetics, Karolinska University Hospital Solna, 171 77 Stockholm, Sweden; 36 Department of Molecular Medicine and Surgery, Karolinska Institutet Solna, 171 77 Stockholm, Sweden; 37 Department of Health Science Research, Mayo Clinic Arizona, Scottsdale, AZ 85259 USA; 38 Biomedicine Institute (IBIOMED), University of León, León, Spain; 39 CIBER Epidemiología y Salud Pública (CIBERESP), 28029 Madrid, Spain; 40 Catalan Institute of Oncology, Bellvitge Biomedical Research Institute (IDIBELL), 08028 Barcelona, Spain; 41 University of Barcelona, 08007 Barcelona, Spain; 42 Department of Medicine, Clinical Genetics Service, Memorial Sloan Kettering Cancer Center, New York, NY 10065 USA; 43 Department of Public Health and Primary Care, Centre for Cancer Genetic Epidemiology, University of Cambridge, Cambridge, CB2 1TN UK; 44 School of Public Health, Imperial College London, London, UK; 45 Unit of Nutrition and Cancer, Cancer Epidemiology Research Program, Catalan Institute of Oncology-IDIBELL, L’Hospitalet de Llobregat, 08908 Barcelona, Spain; 46 Molecular Epidemiology Group, Translational Research Laboratory, Catalan Institute of Oncology-IDIBELL, L’Hospitalet de Llobregat, 08908 Barcelona, Spain; 47 Department of General and Thoracic Surgery, University Hospital Schleswig-Holstein, Campus Kiel, 24118 Kiel, Germany; 48 Department of Genetics and Genome Sciences, Case Western Reserve University, Cleveland, OH 44106 USA; 49 Department of Cancer Epidemiology, H. Lee Moffitt Cancer Center and Research Institute, Inc, Tampa, FL 33612 USA; 50 Department of Gastrointestinal Oncology, H. Lee Moffitt Cancer Center and Research Institute, Inc, Tampa, FL 33612 USA; 51 Centre for Research in Epidemiology and Population Health, Institut de Cancérologie Gustave Roussy, Villejuif, France; 52 Department of Internal Medicine, University of Utah, Salt Lake City, UT USA; 53 Department Genome Sciences, University of Washington, 98195 Seattle, WA USA; 54 Hellenic Health Foundation, 13 Kaisareias & Alexandroupoleos, 115 27 Athens, Greece; 55 WHO Collaborating Center for Nutrition and Health, Unit of Nutritional Epidemiology and Nutrition in Public Health, Department of Hygiene, Epidemiology and Medical Statistics, Medical School, National and Kapodistrian University of Athens, Mikras Asias 75, 115 27 Athens, Greece; 56 Affiliation Cancer Registry, Department of Prevention, Azienda Sanitaria Provinciale di Ragusa, Ragusa, Italy; 57 Population Sciences, Huntsman Cancer Institute, Salt Lake City, UT 84112 USA; 58 Department of Medical Biosciences, Pathology, Umeå University, Umeå, Sweden; 59 Institute of Environmental Medicine, Karolinska Institutet Solna, 17177 Stockholm, Sweden; 60 Department of Surgical Sciences, Uppsala University, 75121 Uppsala, Sweden; 61 Discipline of Genetics, Faculty of Medicine, Memorial University of Newfoundland, Saint John’s, NL A1B 3V6 Canada; 62 Department of Public Health Sciences, University of Virginia School of Medicine, Charlottesville, VA 22908 USA; 63 Vanderbilt-Ingram Cancer Center, Vanderbilt University, Nashville, TN 37232 USA

## Abstract

Genome-wide association studies have reported 56 independently associated colorectal cancer (CRC) risk variants, most of which are non-coding and believed to exert their effects by modulating gene expression. The computational method PrediXcan uses *cis*-regulatory variant predictors to impute expression and perform gene-level association tests in GWAS without directly measured transcriptomes. In this study, we used reference datasets from colon (*n* = 169) and whole blood (*n* = 922) transcriptomes to test CRC association with genetically determined expression levels in a genome-wide analysis of 12,186 cases and 14,718 controls. Three novel associations were discovered from colon transverse models at FDR ≤ 0.2 and further evaluated in an independent replication including 32,825 cases and 39,933 controls. After adjusting for multiple comparisons, we found statistically significant associations using colon transcriptome models with *TRIM4* (discovery *P* = 2.2 × 10^− 4^, replication *P* = 0.01), and *PYGL* (discovery *P* = 2.3 × 10^− 4^, replication *P* = 6.7 × 10^− 4^). Interestingly, both genes encode proteins that influence redox homeostasis and are related to cellular metabolic reprogramming in tumors, implicating a novel CRC pathway linked to cell growth and proliferation. Defining CRC risk regions as one megabase up- and downstream of one of the 56 independent risk variants, we defined 44 non-overlapping CRC-risk regions. Among these risk regions, we identified genes associated with CRC (*P* < 0.05) in 34/44 CRC-risk regions. Importantly, CRC association was found for two genes in the previously reported 2q25 locus, *CXCR1* and *CXCR2*, which are potential cancer therapeutic targets. These findings provide strong candidate genes to prioritize for subsequent laboratory follow-up of GWAS loci. This study is the first to implement PrediXcan in a large colorectal cancer study and findings highlight the utility of integrating transcriptome data in GWAS for discovery of, and biological insight into, risk loci.

**Electronic supplementary material:**

The online version of this article (10.1007/s00439-019-01989-8) contains supplementary material, which is available to authorized users.

## Introduction

It is estimated that genetic variants explain 12–35% of the heritability in colorectal cancer (CRC) risk (Lichtenstein et al. 2000; Czene et al. 2002; Jiao et al. 2014). To date, Genome-Wide Association Studies (GWAS) have identified 56 independent common risk variants that are robustly associated with CRC (Peters et al. 2015; Schumacher et al. 2015; Orlando et al. 2016). However, the functional relevance of most discovered CRC-risk variants (89%) remains unclear. The biological mechanisms linking CRC-associated risk variants with target genes have only been validated in the laboratory for six regions [8q24 *MYC* (Pomerantz et al. 2009), 8q23.3 *EIF3H* (Pittman et al. 2010), 11q23.1 *COLCA1* and *COLCA2* (Biancolella et al. 2014), 15q13.3 *GREM1* (Lewis et al. 2014), 16q22.1 *CDH1* (Shin et al. 2004), and 18q21.1 *SMAD7* (Fortini et al. 2014)]. Given that most of the associated loci do not include coding variants, a large portion of CRC genetic risk is thought to be explained by regulatory variation that modulates the expression of target genes. This hypothesis is supported by the observation that CRC risk variants are enriched in colon expression quantitative trait loci (eQTLs) (Hulur et al. 2015) and active regulatory regions of colorectal enhancers (Bien et al. 2017). Together, this evidence highlights the value of studying transcriptional regulation in relation to CRC risk.

Large-scale efforts are underway to map regulatory elements across tissues and cell types. Many transcriptome studies have been conducted where genotype and expression levels are jointly assayed for many individuals, enabling the discovery of tissue-specific eQTLs. For instance, the Genotype-Tissue Expression (GTEx) Project (GTEx Consortium 2013) is building a biospecimen repository to comprehensively map tissue-specific eQTLs across human tissues, which currently includes transcriptomes from 169 colon transverse samples. These data provide a remarkable new resource for understanding function in non-coding regions that can be used to inform GWAS.

We employed the computational method, PrediXcan (Gamazon et al. 2015), to perform a CRC transcriptome-wide association study using reference datasets to ‘impute’ unobserved expression levels into GWAS datasets. Variant prediction models were developed using colon transverse transcriptomes (*n* = 169) from GTEx (GTEx Consortium 2013) and a larger whole blood transcriptome panel (*n* = 922) from the depression genes and networks (DGN) (Battle et al. 2014). We included whole blood as a previous analysis demonstrated that gene regulatory elements of immune cell types from peripheral blood are enriched for variants with more significant CRC association *P* (Bien et al. 2017). Further, laboratory follow-up of the CRC GWAS locus 11q23 implicates two genes, *COLCA1* and *COLCA2*, which are co-expressed in immune cell types and correlate with inflammatory processes (Peltekova et al. 2014). In addition to novel discovery, the PrediXcan approach can aid in prioritization of candidate target genes in non-coding GWAS loci and thereby inform testable hypotheses for laboratory follow-up. Therefore, as a secondary analysis we investigated the association of imputed gene expression with CRC in the 44 genetic regions harboring one or more of the 56 independent variants (*r*^2^ < 0.2) that are associated with CRC in previous GWAS (*P* ≤ 5 × 10^− 8^) and were replicated in an independent dataset.

**Fig. 1:**
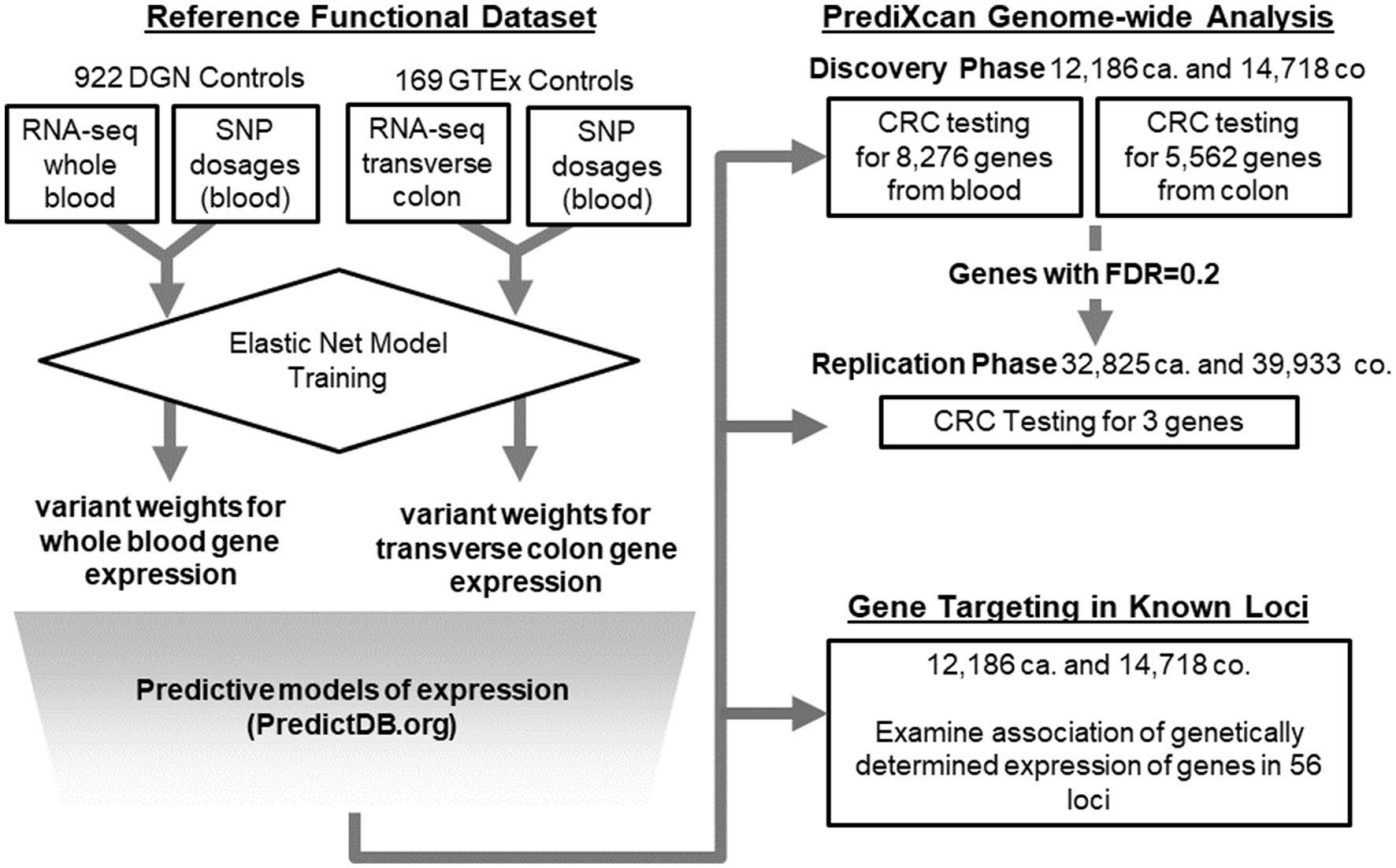
Schematic illustration of the study design training data was comprised of joint observations of imputed variant genotypes and tissue-specific gene expression from reference datasets (DGN and GTEx). Elastic net regularization was used to train genetic variant predictors of gene expression and downloaded from PredictDB.org. Models for colon transverse tissues and whole blood were used for imputation of expression into independent GWAS datasets for Colorectal Cancer (CRC). Imputed gene expression was then tested for association with case (ca.)–control (co.) status in the discovery stage. Novel gene associations with a false discovery rate (FDR) = 0.2 were assessed in an independent CRC GWAS dataset. As a secondary analysis, the association of genetically determined expression of genes in 44 GWAS-associated risk regions was examined

We aimed to discover novel loci associated with CRC, and refine established regulatory risk loci by reducing the list of putative gene targets. Employing PrediXcan, we tested genetically regulated gene expression for association with CRC in a two-stage approach. In the discovery stage, up to 8277 gene sets were tested in 12,186 cases and 14,718 controls from the Genetics and Epidemiology of Colorectal Cancer Consortium (GECCO) and the Colon Cancer Family Registry (CCFR). This discovery set was also used to identify potential target genes in the 44 genetic regions harboring 56 known CRC risk variants. We attempted replication of three novel genes that were not positioned within 1 Mb of the 56 previously reported risk variants and with false discovery rate (FDR) ≤ 0.2 for CRC risk in a large and independent study of 32,825 cases and 39,933 controls from the Colorectal Transdisciplinary (CORECT) consortium, UK Biobank, and additional CRC GWAS (Fig. 1).

## Results

### Imputation of genetically regulated gene expression

Gene expression levels were imputed using previously published multi-variant models built using elastic net regularization (variant weight gene models V6 available online from PredictDB.org). For each tissue and gene, a quality metric referred to as predictive *R*^2^ was provided as the correlation between the observed and predicted expression from the multi-variant model based on a tenfold cross validation. After restricting to protein coding genes with a predictive *R*^2^ > 0.01 (≥ 10% correlation between predicted and observed expression), the discovery analysis tested the association of imputed expression for 4850 genes using colon transverse models and 8277 genes using whole blood models. On average, colon transverse models used 22 variants (SD = 19) per gene with a range of 1–173 variants. The number of variants in whole blood models were slightly larger on average with a mean of 34 variants (SD = 24) per gene, ranging from 1 to 213 variants. We report CRC association results and predictive *R*^2^ for imputed expression of each gene with *P* ≤ 0.05 in either colon transcriptome or whole blood analysis (Online Resource 2 Table S2).

### Discovery of new CRC susceptibility genes

In total, multivariate logistic regression was used to test the association of CRC with genetically impute gene expression for 4850 genes from colon transverse models and 8277 genes from whole blood models. We employed PrediXcan in 12,186 cases and 14,718 controls from 16 GWAS studies. Replication was attempted for associations meeting an FDR = 0.2 threshold in the discovery phase if they were in a novel CRC region using an independent GWAS dataset comprised of 32,825 cases and 39,933 controls from the CORECT consortium, UK Biobank, and additional GWAS as described in Online Resource 1. In the discovery phase, colon transcriptome models identified CRC association with imputed genetically regulated gene expression in three putative novel regions. Two out of three genes tested in the replication dataset were significant after adjusting for multiple comparisons (*α* = 0.05/3 = 0.017) (Online Resource Fig S1, Table 1). In addition to being more than 1 Mb away from previously identified risk variants, we confirmed that none of the variant predictors used to impute gene expression for these three genes were in LD (*r*^2^ ≤ 0.1) with previously published CRC-risk variants. In the 7q22.1 locus, increased expression of *TRIM4* was associated with reduced CRC risk with an odds ratio (OR) of 0.94 [95% confidence interval (CI) 0.91–0.97, discovery *P* = 2.2 × 10^− 4^]. Reduced CRC risk was also statistically associated with increased genetically regulated gene expression of *TRIM4* in the independent replication dataset (*P* = 0.01). The second novel locus, 14q22.1, was also found to be inversely associated, where increased genetically regulated gene expression of *PYGL* was associated with decreased CRC risk, showing an OR of 0.90 (95% CI 0.85–0.96) in the discovery dataset (discovery *P* = 2.3 × 10^− 4^) as well as in the replication dataset (*P* = 7.9 × 10^− 4^). Imputed genetically regulated gene expression for *SLC22A31* was associated with increased CRC risk in the discovery phase (*P* = 1.3 × 10^− 4^), but did not replicate in the independent dataset. We found no associations in novel regions using whole blood variant models that reached FDR = 0.2 in the discovery phase.

**Table 1:** Genes passing discovery threshold in novel loci from colon transverse PrediXcan

Locus	Gene	Direction of gene expression for increased CRC risk	Discovery (*n* ca./co. = 12,186/14,718)	Replication (*n* ca./co. = 32,825/39,939)	PrediXcan gene model information	
			*P*	*P*	*R* ^2^	Number of predictive variants
7q22.1	*TRIM4*	Decrease	1.7 × 10^− 4^	1.1 × 10^− 2^	0.51	62
14q22.1	*PYGL*	Decrease	2.3 × 10^− 4^	8.7 × 10^− 4^	0.26	23
16q24.3	*SLC22A31*	Increase	1.3 × 10^− 4^	0.62	0.14	29

*P* For the association between CRC and the genetically determined gene expression in discovery and replication GWAS studies

*R*^2^ = the cross-validated *R*^2^ value found when training the model (predictive *R*^2^ from PredictDB.org). Replicated at *α* = 0.05/3 genes = 1.7 × 10^− 2^

Colon Transverse PrediXcan analyses were repeated for *TRIM4* and *PYGL* in the discovery dataset stratifying cases by proximal (*n* = 4454 cases), distal (*n* = 3580 cases), and rectal (*n* = 2936 cases) cancer sites. We excluded 1216 cases from the stratified analysis because the colon cancer site was unspecified. We found that for both genes the effects and p values were similar between the three sites. For *TRIM4*, the CRC association with genetically imputed gene expression had an OR of 0.94 (95% CI 0.90–0.98, *P* = 3 × 10^− 3^) in proximal colon cases compared to an OR of 0.95 (95% CI 0.90–1.0, *P* = 5 × 10^− 2^) in distal colon cases and an OR of 0.93 (95% CI 0.88–0.98, *P* = 2 × 10^− 2^) in rectal cases. There was no significant difference in the effect estimates between these cancer sites for *TRIM4* (*Q*-test for heterogeneity *P* = 1.0). Similarly, for *PYGL*, the CRC association with genetically regulated gene expression had an OR of 0.89 (95% CI 0.82–0.97, *P* = 3 × 10^− 3^) in proximal colon cases compared to an OR of 0.91 (95% CI 0.83–1.0, *P* = 2 × 10^− 2^) in distal colon cases and an OR of 0.86 (95% CI 0.77–0.95, *P* = 5 × 10^− 4^) in rectal cases with no significant difference in effects (*Q* test for heterogeneity *P* = 0.98).

We further investigated the replicated CRC-associated PrediXcan genes by summarizing the single-variant CRC association results for variants that were included in the prediction models, referred to hereafter as ‘variant predictors’ (Online Resources 3–6 Fig S2). In *TRIM4*, the association was mostly driven by one LD block with 62 correlated genetic variant predictors used to impute genetically regulated gene expression in colon tissue models. Among the variant predictors of *TRIM4*, rs2527886 was most significantly associated with CRC (*P* = 1.8 × 10^− 4^). Bioinformatic follow-up of the *TRIM4* locus showed that in the genomic region containing variants correlated with rs2527886, there were six enhancers with strong Chromatin Immunoprecipitation Sequencing (ChIP-seq) H3K27ac signal in either normal colorectal crypt cells or a CRC cell line (Online Resource 1 Fig S3). Using peak signal from H3K27ac activity to define enhancer regions, two enhancers were gained in ten or more CRC cell lines compared to normal colorectal crypt cells, referred to as recurrent variant enhancer loci (VEL) (Akhtar-Zaidi et al. 2012). Rs2527886 is positioned within one of these VEL. Peak ChIP-seq binding region for CTCF suggests that the VEL harboring rs2527886 may be in physical contact with the *TRIM4* promoter. In the same VEL, one of the LD variants, rs2525548 (LD *r*^2^ = 0.99), is positioned within transcription factor binding sites for RUNX3, FOX, NR3C1, and BATF (Online Resource 1 Fig S3). In the *PYGL* locus, rs12589665 is the variant predictor with the strongest marginal association with CRC (*P* = 3.2 × 10^− 4^). We identified 7 enhancers in the region spanning the variants in LD with rs12589665, and three variants in LD with the lead predictor variant were positioned in VEL. Two of these variants, rs72685325 (*r*^2^ = 0.62) and rs72685323 (*r*^2^ = 0.53), were positioned within binding sites for 7 transcription factors (Online Resource 1 Fig S3).

A series of exploratory analyses were conducted to assess whether the observed inflation in association signals (*λ* = 1.1) was the result of bias in our data or modeling error. Results suggest that inflation was not driven by genes with low predictive *R*^2^ values (Online Resource 1 Fig S4), other potential confounding factors common to GWAS like genotyping batch effects (Online Resource 1 Fig S5) or cryptic population structure (Online Resource 1 Fig S6–S7), or due to inflated *Z* statistics by modeling genes with little variability in expression (Online Resource 1 Fig. S8–S11). Observed inflation was slightly reduced, but still elevated when looking at the marginal association results for the variant predictors (*λ* = 1.07; Online Resource 1 Fig S12) and when excluding genes with high predicted co-expression (*λ* = 1.07; Online Resource 1 Fig S13). Collectively, this exploration suggests that the observed inflation is less likely to be the result of modeling or analytical error and more likely reflects the polygenicity of CRC.

### Refinement of known CRC GWAS-risk regions

We first assembled a list of 56 previously reported independent (*r*^2^ ≤ 0.2) CRC GWAS risk variants and defined a distance-based region surrounding each variant as the chromosomal position of the first reported (index) variant ± 1 Mb (Online Resource 1 Table S3). We then combined overlapping risk regions by taking the minimum and maximum chromosomal positions of all regions that overlapped, resulting in a total of 44 CRC risk regions harboring 1–4 independent CRC-risk variants. In these 44 regions, there was an average of 20 (SD ± 17) protein-coding genes per region annotated by the Consensus Coding Sequence Database (CCDS). The average number of protein-coding genes per region with imputed genetically regulated gene expression in the tissue-specific models was reduced to an average of 10 (SD ± 8) genes in colon transverse, and 14 (SD ± 11) genes in whole blood. Further, in these regions we found that of the total number of genes with genetically regulated gene expression across the two models, an average 45% of the genes overlapped. We found that 34/44 (77%) of CRC-risk regions overlapped the transcription start site of a gene associated with CRC at a *P* < 0.05. Comparing the number of genes with a *P* < 0.05 to the total number of CCDS genes within 1 Mb of an index variant resulted in an average reduction of 82% per region (Table 2).

**Table 2:** Known GWAS-risk regions overlapping genes that show association of genetically regulated gene expression with CRC

Region	Gene count in region				PrediXcan results for genes with *P* ≤ 0.05					GWAS publication for independent index variant(s)^c^			Variant(s) with differential allelic effects and gene regulated
	CCDS gene build	Genes with genetically imputed gene expression^a^			Gene set (decreasing order of significance)		Number of genes (% reduced from CCDS)^b^	*P* for most significant gene		Reported gene(s)	rsID dbSNP function (note)	References	
		CT	WB	CT∩WB (% overlap)^d^	CT	WB	CT + WB	CT	WB				
1p36.12	20	11	16	9 (50)	*–*	*CDC42*	1 (95)	–	0.02	*WNT4, CDC42*	rs72647484, intergenic	Al-Tassan et al. (2015)	–
1q25.3	19	8	14	7 (47)	*ARPC5*	*LAMC1, RGL1, TEDDM1*	4 (79)	0.02	3 × 10^− 6^	*LAMC1*	rs10911251, intronic	Peters et al. (2013)	–
1q41	8	6	8	5 (56)	*MIA3*	*FAM177B, AIDA*	3 (63)	0.05	0.02	*DUSP10*	rs6691170, intergenic	Houlston et al. (2010)	–
2q35	41	12	34	10 (27)	*GPBAR1, WNT10A, ARPC2*	*CXCR1, CXCR2, ARPC2, AAMP, PNKD, GPBAR1, TMBIM1*	8 (80)	3 × 10^− 3^	8 × 10^− 5^	*PNKD, TMBIM1*	rs992157, intronic (tags missense)	Orlando et al. (2016)	–
3p22.1	9	2	5	2 (40)	*–*	*ZNF621*	1 (88)	–	0.04	*CTNNB1*	rs35360328, intergenic	Schumacher et al. (2015)	–
3p14.1	4	2	4	2 (50)	*SLC25A26*	*SLC25A26, SUCLG2*	1 (75)	2 × 10^− 3^	1 × 10^− 3^	*SLC25A26, LRIG1*	rs812481, intronic	Schumacher et al. (2015)	–
5p15.33	20	17	16	10 (43)	*PDCD6*	*AHRR*	2 (90)	0.02	0.02	*TERT*	rs2736100, intronic	Kinnersley et al. (2012)	–
5q22.2	8	6	6	6 (100)	*SRP19*	*–*	1 (87)	9 × 10^− 3^	–	*APC*	rs1801155, missense-*APC*	Niell et al. (2003)	–
5q31.1	24	10	14	8 (50)	*–*	*CAMLG, DDX46*	2 (92)	*–*	0.04	*PITX1, CATSPER3, PCBD2, MIR4461, H2AFY*	rs647161, intergenic	Jia et al. (2013)	–
6p21.2	31	21	24	15 (50)	*–*	*ETV7, KCTD20, C6orf89, PXT1*	4 (87)	–	0.01	*CDKN1A*	rs1321311, intergenic	Dunlop et al. (2012)	–
6p21.1	30	14	22	10 (38)	*–*	*UBR2*	1 (97)	*–*	0.01	*TFEB*	rs4711689, intronic	Zeng et al. (2016)	–
6q22.1	16	9	6	3 (25)	*DCBLD1, ROS1, VGLL2*	*DCBLD1*	3 (81)	9 × 10^− 3^	0.01	*DCDBL2*	rs4946260, intronic	Schumacher et al. (2015)	–
6q25.3	16	11	11	8 (57)	*MAP3K4*	*–*	1 (94)	7 × 10^− 3^	*–*	*SCL22A3*	rs7758229	Cui et al. (2011)	–
8q23.3	6	5	5	3 (43)	*AARD, SLC30A8*	*UTP23*	3 (50)	0.02	6 × 10^− 3^	*EIF3H*	rs2450115, intergenic; rs16892766, intergenic; rs6469656, intergenic	Tomlinson et al. (2008) and Zeng et al. (2016)	rs16888589; *EIF3H*
8q24.21	5	2	4	2 (67)	*POU5F1B, FAM84B*	*POU5F1B*	2 (60)	6 × 10^− 10^	0.01	*POU5F1B, MYC*	rs6983267, intergenic	Tomlinson et al. (2008)	rs6983267; *MYC*
9q24	11	8	9	6 (55)	*KIAA1432*	*–*	1 (91)	0.03	*–*	*Not reported*	rs719725, intergenic	Zanke et al. (2007)	–
10p14	5	2	4	2 (50)	*ITIH2*	*–*	1 (80)	0.01	*–*	*GATA3*	rs10795668	Tomlinson et al. (2008)	–
10q24.2	74	26	42	9 (53)	*CUTC, HIF1AN, SEC31B*	*SLC25A28, COX15, SEC31B, HIF1AN, ENTPD7*	6 (70)	5 × 10^− 3^	9 × 10^− 4^	*ABCC2, MRP2*	rs1035209, intergenic	Whiffin et al. (2014)	–
10q25.2	12	6	7	5 (63)	*–*	*GPAM*	1 (92)	*–*	0.05	*VTI1A, TCF7L2*	rs12241008, intronic; rs11196172	Zhang et al. (2014) and Wang et al. (2017)	–
11q12.2	74	26	42	15 (28)	*FADS2, GANAB*	*C11orf10, FADS1, FADS2,TAF6L, C11orf9, DAGLA, FADS3*	8 (89)	4 × 10^− 3^	5 × 10^− 4^	*MYRF*	rs174537, intronic rs60892987, intergenic	Zhang et al. (2014) and Schmit et al. (2016)	–
11q13.4	29	14	22	9 (33)	*OR2AT4, RNF169, NEU3, DNAJB13*	*POLD3, RAB6A, MRPL48*	4 (67)	7 × 10^− 5^	8 × 10^− 3^	*POLD3*	rs3824999, intronic	Dunlop et al. (2012)	–
11q23.1	27	14	13	8 (42)	*COLCA2, COLCA1, C11orf53, DLAT*	*–*	4 (85)	1 × 10^− 6^	–	*COLCA1, COLCA2*	rs3802842, intronic	Tenesa et al. (2008)	rs7130173; *COLCA1, COLCA2*
12p13.32	71	40	53	33 (55)	*NOP2*	*CCND2, SCNN1A*	3 (96)	0.04	6 × 10^− 3^	*CCND2, C12orf5, FGF6, RAD51AP1, FGF23, PARP11*	rs10774214, intergenic; rs3217810, intergenic rs10849432, intergenic rs11064437, splice donor-*SPSB2*	Jia et al. (2013), Zhang et al. (2014), Whiffin et al. (2014) and Zeng et al. (2016)	–
12q13.12	32	16	20	9 (33)	*LIMA1, COX14, CERS5, NCKAP5L, LETMD1, ATF1*	*DIP2B, LIMA1, SMARCD1, GALNT6, TFCP2, SCN8A, METTL7A, RACGAP1*	13 (59)	8 × 10^− 6^	3 × 10^− 4^	*DIP2B, ATF1*	rs11169552, intronic	Houlston et al. (2010)	–
12q24.12	24	12	18	9 (43)	*HECTD4, RAD9B, BRAP, TMEM116, FAM109A*	*TRAFD1, CUX2, BRAP, ATXN2, SH2B3*	9 (63)	2 × 10^− 3^	1 × 10^− 6^	*SH2B3*	rs3184504, missense	Schumacher et al. (2015)	–
12q24.22	14	6	11	4 (31)	*NOS1*	*FBXO21*	2 (86)	1 × 10^− 2^	9 × 10^− 3^	*NOS1*	rs7320812	Schumacher et al. (2015)	–
15q13.3	9	5	2	2 (40)	*GOLGA8N*	–	1 (88)	0.04	–	*GREM1*	rs16969681 intergenic rs11632715, intergenic	Tomlinson et al. (2007)	rs16969681;*GREM1*
16q22.1	41	23	35	19 (49)	–	*ESRP2, NFATC3*	2 (98)	–	8 × 10^− 3^	*CDH1*	rs9929218, intronic	COGENT Study et al. (2008)	rs5030625; *CDH1*
17p13.3	27	19	24	17 (65)	FAM57A, GEMIN4, BMLHA9	*FAM57A, GEMIN4*	3 (89)	1 × 10^− 3^	0.01	*NXN*	rs12603526, intronic	Zhang et al. (2014)	–
18q21.1	10	5	7	3 (33)	*MYO5B, LIPG*	*SMAD7*	3 (70)	8 × 10^− 3^	0.04	*SMAD7*	rs7229639 intronic rs4939827 intronic	Broderick et al. (2007) and Zhang et al. (2014)	rs6507874, rs6507875, rs8085824, and rs5892087, *SMAD7*
19q13.11	20	13	17	11 (58)	*PDCD5*	*PDCD5*	1 (95)	0.04	0.02	*RHPN2, GPATCH1*	rs10411210 intronic	COGENT Study et al. (2008)	–
19q13.2	59	24	37	15 (33)	*DEDD2, TGFB1*	*SNRPA, B3GNT8, CCDC97*	5 (92)	0.03	6 × 10^− 3^	*TGFB1, B9D2*	rs1800469 intronic (tags missense)	Zhang et al. (2014)	–
20q13.13	9	4	7	3 (38)	*PREX1*	*B4GALT5*	2 (78)	7 × 10^− 3^	7 × 10^− 3^	*PREX1*	rs6066825 intronic	Schumacher et al. (2015)	–
20q13.33	27	20	23	15 (54)	*MTG2*	*SS18L1, HRH3*	3 (89)	0.05	5 × 10^− 3^	*LAMA5, RPS21*	rs4925386 intronic	Houlston et al. (2010)	–

CCDS genes were counted, regardless of tissue relevance, 500 kb upstream or downstream of an index variant

*CT* colon transverse, *WB* whole blood, *No*. number*—* no genes meeting criteria. In known loci, genes with gene expression predictive *R*^2^ < 0.01 were included

^a^Genes with predicted expression in the corresponding tissue

^b^Number of genes with a *P* value ≤ 0.05. % Red. = (# of genes with *P* value ≤ 0.05/# CCDS genes) × 100

^c^Conditionally independent in statistical models containing both variants or LD *r*^2^ < 0.2

^d^The intersect of genes in CT and WB models

We further investigated the regions that did not show evidence of gene association and found that GWAS reported risk variants in 3/10 of these regions were a coding variant or were in LD with a coding variant (3q26-*MYNN* and *LRRC34*, 10q24.32-*WBP1L*, 14q22.2-*BMP4*). Additionally, 2/10 of the risk variants were originally discovered in East Asian populations and risk SNPs had weaker association in our study (10q22.3-rs704017 *P* = 1 × 10^− 4^ and 10q24.32-rs4919687 *P* = 1 × 10^− 2^). Another 2/10 GWAS risk variant did not replicate in our study (4q31.1-rs60745952 *P* = 0.8 and 16p13.2-rs79900961 *P* = 0.26). In the remaining 3/10 regions, we found that the index variants did not reach genome-wide significance, reflecting power limitations in our discovery dataset (4q32.2-rs35509282 *P* = 6 × 10^− 3^, 16q24.1-rs16941835 *P* = 4 × 10^− 3^, and 20p12.3-rs961253 *P* = 4 × 10^− 5^).

Among the 34 regions containing associated genes, we found that the most significant gene association in the PrediXcan analysis was often the strongest candidate based on either known CRC etiology and gene function or results from previous laboratory follow-up (e.g. *COLCA2, LAMC1, POLD3, SMAD7, TGFB1*). In addition to confirming suspected genes, new candidates were also identified. For example, *CXCR1* (*P* = 8 × 10^− 5^) and *CXCR2* (*P* = 9 × 10^− 5^) were among the strongest associations. Notably, these genes are biologically relevant targets given that they encode cytokine receptors known to be implicated in a variety of cancers.

## Discussion

In this study, we employed the PrediXcan in 12,186 cases and 14,718 controls. Genetic variant predictors of gene expression from both colon transverse and whole blood transcriptomes were used to test the association of CRC risk with imputed gene expression. We replicated novel associations of *TRIM4* and *PYGL* in a large independent study of over 70,000 participants. In addition, we identified strong gene targets in several known GWAS loci, including genes that were previously not reported as putative candidates.

The two novel gene associations discovered in colon transverse models implicate genes involved with hypoxia-induced metabolic reprogramming, which is a hallmark of tumorigenesis in solid tumors. *TRIM4* is a member of a superfamily of ubiquitin E3 ligases comprised of over 70 genes notably defined by a highly conserved N-terminal RING finger domain. This family of proteins has been implicated in a number of oncogenic or tumor suppressor activities that involve pathways related to CRC (Myc, Ras, etc.) (Sato et al. 2012; Chen et al. 2012; Zaman et al. 2013; Tocchini et al. 2014; Zhou et al. 2014; Zhan et al. 2015), and recently have been implicated in inflammatory and immune related activities (Eames et al. 2012; Versteeg et al. 2014). Somatic alterations in other *TRIM* genes have been associated with a large number of cancers including colon (Glebov et al. 2006; Noguchi et al. 2011; Hatakeyama 2011). While *TRIM4* has not previously been implicated in cancer risk, the strong homology across gene members of this family and their implications in cancer and immunity make this gene an interesting candidate. Moreover, a recent study suggests that expression of *TRIM4* plays a role in sensitizing cells to oxidative stress-induced death and regulation of reactive oxygen species (ROS) levels (H_2_O_2_) through ubiquitination of the redox regulator peroxide reductase (Tomar et al. 2015). Regulation of ROS levels and the cellular antioxidant system has previously been implicated in the pathophysiology of many diseases including inflammation and tumorigenesis (López-Lázaro 2007; Holmdahl et al. 2013). ROS are associated with cell cycle, proliferation, differentiation and migration and are elevated in colon as well as other cancers (Vaquero et al. 2004; Kumar et al. 2008; Afanas’ev 2011; Lin et al. 2017). Notably, many of the established environmental risk factors for colon cancer implicate oxidative stress pathways, including high alcohol consumption, smoking, increased consumption of red and processed meats (Stevens et al. 1988; Bird et al. 1996), or decreased consumption of fruits and vegetables (La Vecchia et al. 2013). In future laboratory analysis, it would be interesting to investigate whether the association of increased *TRIM4* expression with decreased CRC risk is mechanistically acting through the regulation of ROS and cell growth.

Under the hypoxic conditions of the tumor microenvironment, constant reprogramming of glycogen metabolism is essential for providing the energy requirements necessary for cell growth and proliferation. *PYGL* (the second novel finding) encodes the key enzyme involved in glycogen degradation, releases glucose-1-phosphate so that it can enter the pentose phosphate pathway, which is important for generating NADPH, nucleotides, amino acids, and lipids required for continued cell proliferation (Favaro et al. 2012). It has previously been shown that depletion of *PYGL* leads to oxidative stress (increased ROS levels), and subsequent P53-induced growth arrest in cancer cells (Favaro et al. 2012). Of note, small molecule inhibitors of *PYGL* are currently under investigation for the treatment of diabetes (Praly and Vidal 2010). However, while decreased expression of *PYGL* in the tumor may result in tumor senescence, our results suggest that decreased *PYGL* expression is associated with increased risk of CRC. Like the dynamic role of expression for genes involved in the TGF-beta pathway, these conflicting observations between cancer risk and effects of early versus late induction of *PYGL* on cancer survival are likely reflecting the importance of context and fluctuating nutrient and oxygen availability within the tumor microenvironment.

Importantly, we found that the PrediXcan analysis identified new candidate genes in known GWAS loci that had previously gone undetected. For instance, in the recently identified 2q35 locus (Orlando et al. 2016), the authors originally reported the two closest genes, *PNKD* and *TMBIM1*, as potential targets for the putative regulatory locus marked by the index variant, rs992157. The authors reported eQTL evidence showing that rs992157 was associated with expression of nearby genes *PNKD* and *TMBIM1* in lymphoblastoid cells, but not colorectal adenocarcinoma cells. In our PrediXcan analysis, expression of two other genes in this region, *CXCR1* and *CXCR2*, were among the most strongly associated genes in the entire analysis, while the associations for *PNKD* (*P* = 6 × 10^− 3^) and *TMBIM1* (*P* = 0.01) showed weaker associations. Our study added independent evidence for an association of the locus with CRC given that the index variant was only borderline significantly associated in previous analysis and identify two promising targets, *CXCR1* and *CXCR2*. These genes are of note due to their chemotherapeutic properties. Specifically, the CXCR inhibitor, Reparixin, is currently under investigation for progression free survival of metastatic triple negative breast cancer in a stage 2 clinical trial (NCT02370238). Interestingly, expression of *CXCR1* and *CXCR2* has been shown to be elevated in colon tumor epithelium relative to normal adjacent tissue (*P* < 0.001). While there is still much to be learned, it is possible that this drug could also be useful for the treatment of CRC (Dabkeviciene et al. 2015).

This study had many strengths, most notably the use of reference transcriptome data to perform gene-level association testing in several large GWAS studies to both uncover novel associations and identify likely functional gene targets in known loci. By integrating reference transcriptome data, this study focused on genes that are expressed in CRC-relevant tissues. Furthermore, this method provided biologically relevant sets to aggregate variants, thereby improving statistical power by reducing the burden of multiple comparisons. In addition, our study was quite large, being comprised of nearly 100,000 participants across the discovery and replication datasets.

Our study had several limitations. For many genes, the predictive *R*^2^ for genetic variant models was relatively low, indicating that a small proportion of the variance in gene expression was explained by these models. In a recent publication, Su et al. (2018) demonstrated through extensive simulations that while there is an attenuation of true signal as a results of this, the diminishment in power was less than anticipated and more importantly this does not increase type I error. Predictive performance values were relatively strong in the models used for *PYGL* (*R*^2^ = 0.26) *TRIM4*. (*R*^2^ = 0.51) corresponding to 51% and 71% correlation between predicted and observed expression, respectively. In general, larger sample sizes for the reference panel will be needed to achieve better prediction models, particularly for rarer variants. While *PYGL* and *TRIM4* were discovered using the colon tissue model, the whole blood model also showed evidence of association. This finding was not surprising in light of the recent GTEx paper demonstrating that many GWAS loci implicate shared eQTLs (GTEx Consortium et al. 2017). It should also be noted that variant predictors could implicate enhancers influencing the expression of multiple genes and because this study only evaluates genetically influenced expression levels, there is uncertainty that the associated gene is the causally related gene. As such, laboratory follow-up remains a critical extension of these findings; however, this laborious work can now be more targeted based on results from this analysis.

The loci identified using GWAS are most often located in non-coding regions and provide little biological insight. In contrast, the PrediXcan method directly tests putative target genes providing strong hypotheses for subsequent laboratory follow-up. The *CXCR1* and *CXCR2* findings are of interest given their therapeutic potential. As such, these findings provide preliminary support for new molecular targets that could potentially repurpose a putative cancer therapeutic agent and highlight the utility of integrating functional data for discovery of, and biological insight into risk loci.

Future analyses would be improved by increasing the number of transcriptomes. Similarly, larger GWAS sample sizes, or imputation of other molecular phenotypes (ChIP-seq, DNase-Seq, etc.) as data become available could be fruitful in the identification of important enhancer(s) or other regulatory elements that could influence the expression of one or more genes.

In conclusion, we identified two novel loci through the association of genetically predicted gene expression for *TRIM4* and *PYGL* with CRC risk and identified strong target genes in known loci. The *CXCR1* and *CXCR2* findings highlight the advantage of using gene-based methods to identify stronger candidate genes and potentially expedite clinically relevant discovery. Further functional studies are required to confirm our findings and understand their biologic implications. This, in turn, could provide further insight into CRC etiology and potentially new therapeutic targets.

## Materials and methods

### Description of study cohorts

The discovery phase was comprised of 26,904 participants (12,186 CRC cases and 14,718 controls) of European ancestral heritage across 16 studies (described in methods and materials of Online Resource 1). Details of genotyping, QC and single-variant GWAS have been previously reported (Peters et al. 2013; Schumacher et al. 2015). The replication phase included a total of 32,825 cases and 39,933 controls. In addition to previously published CRC GWAS studies from CORECT (Schumacher et al. 2015) we included UK Biobank (application number 8614) and new CRC GWAS from additional GWAS. A nested case–control dataset from the UK Biobank resource was constructed defining cases as subjects with primary invasive CRC diagnosed, or who died from CRC according to ICD9 (1530–1534, 1536–1541) or ICD10 (C180, C182–C189, C19, C20) codes. Control selection was done in a time-forward manner, selecting one control for each case, first from the risk set at the time of the case’s event, and then multiple passes were made to match second, third and fourth controls. For prevalent cases, each case was matched with four controls that exactly matched the following matching criteria: year at enrollment, race/ethnicity, and sex. In total, 5356 cases and 21,407 matched controls were included from UK Biobank in the replication analysis. For the site-stratified analysis, “proximal” colon cancer was defined as hepatic flexure, transverse colon, cecum and ascending colon (ICD9 1530,1531,1534,1536), “distal” colon cancer was defined as descending colon, sigmoid colon, and splenic flexure (ICD9 1532,1533,1537) and “rectal” was defined as rectosigmoid junction, and rectum (ICD9 1540,1541).

Studies, sample selection and matching are described in Online Resource 1, which provides details on sample numbers, and demographic characteristics of study participants. All participants provided written informed consent, and each study was approved by the relevant research ethics committee or institutional review board.

### Whole-genome sequencing reference genotype imputation panel

We performed low-pass whole-genome sequencing of 2192 samples (details in Online Resource 1) at the University of Washington Sequencing Center (Seattle, WA, USA). A detailed description is provided in the Online Resource 1. In brief, after sample QC and removal of samples with estimated DNA contamination > 3% (16), duplicated samples (5) or related individuals (1), sex discrepancies (0), and samples with low concordance with genome-wide variant array data (11), there were a total of 1439 CRC cases and 720 controls of European ancestry available for subsequent imputation. These data were used as a reference imputation panel for the discovery and replication GWAS datasets.

### GWAS genotype data and quality control

In brief, genotyped variants were excluded based on call rate (< 98%), lack of Hardy–Weinberg Equilibrium in controls (HWE, *P* < 1 × 10^− 4^), and low minor allele frequency (MAF < 0.05). We imputed the autosomal variants of all studies to an internal imputation reference panel derived from whole genome sequencing (described above). We employed a two-stage imputation strategy (Howie et al. 2012) where entire chromosomes were first pre-phased using SHAPEIT2 (Delaneau et al. 2013), followed by imputation using minimac3 (Das et al. 2016). Only variants with an imputation quality *R*^2^ > 0.3 were included for subsequent analyses.

### Imputation of genetically regulated gene expression in study cohort

Jointly measured genome variant data and transcriptome data sets were used by Gamazon et al. to develop additive models of gene expression levels. The weights for the estimation were downloaded from the publicly available database (http://hakyimlab.org/predictdb/). We used these models to estimate genetically regulated expression of genes in colon transverse, and whole blood. These estimates represent multi-variant prediction of tissue-specific gene expression levels.

In-depth details of the reference cohort, datasets, and model building have previously been described (Gamazon et al. 2015). To summarize, jointly measured genome-wide genotype data and RNA-seq data were obtained from two different projects: (1) the DGN cohort (Battle et al. 2014) (whole blood, *n* = 922) and (2) GTEx (GTEx Consortium 2015) (transverse colon, *n* = 169), predominantly of European ancestry. Gamazon et al. used approximately 650,000 variants with MAF > 0.05 to impute non-genotyped dosages using the 1000G Phase 1 v3 reference panel variants with MAF > 0.05 and imputation *R*^2^ > 0.8 was retained for subsequent model building. In each tissue, Gamazon et al. normalized gene expression by adjusting for sex, the top 3 principal components (derived from genotype data) and the top 15 PEER factors (to quantify hidden experimental confounders). These genomic and transcriptomic data sets were used to train additive models of gene expression levels with elastic net regularization (Gamazon et al. 2015). The model can be written as

1 $${Y_g}=\mathop \sum \limits_{k} {w_{k,g}}{X_k}~+~\varepsilon ,$$

where *Y*_*g*_ is the expression trait of gene *g, w*_*k,g*_ is the effect size of genetic marker *k* for *g, X*_*k*_ is the number of reference variant alleles of marker *k* and *ε* is the contribution of other factors influencing gene expression. The effect sizes (*w*_*k,g*_) in Eq. (1) were estimated using the elastic net penalized approach. The summation in Eq. 1 is referred to as the genetically determined component of gene expression. The variant models (weights, *w*_*k,g*) were downloaded from the publicly available database (http://hakyimlab.org/predictdb/).

The heritability of gene expression was used to estimate how well the variant models predict gene expression levels. The narrow-sense heritability for each gene was calculated by Gamazon et al. (2015), using a variance-component model with a genetic relationship matrix (GRM) estimated from genotype data, as implemented in GCTA (Yang et al. 2011). The proportion of the variance in gene expression explained by these local variants was calculated using a mixed-effects model (Torres et al. 2014; Gamazon et al. 2015). This heritability was highly correlated with the predictive *R*^2^ (The cross-validated *R*^2^ value found when training the model). Only genes with *R*^2^ ≥ 0.01(≥ 10% correlation between predicted and observed expression) were tested for association with CRC. Furthermore, this analysis focused on the component of heritability driven by variants in the vicinity (1 Mb) of each gene (*cis*-variants) because the component based on distal variants could not be estimated with enough accuracy to make meaningful inferences.

Genotypes were treated as continuous variables (dosages). Using the variant weights provided by Gamazon et al. we estimated the genetically regulated gene expression (GReX) of each gene *g*

2 $${\text{GReX}}=~\mathop \sum \limits_{k} {w_{k,g}}{x_k},$$

where *w*_*k*_ is the single-variant coefficient derived by regressing the gene expression trait *Y* on variant *X*_*k*_ using the reference transcriptome data. To address linkage disequilibrium among variant predictors, Gamazon et al. (2015) used the variable selection method to select a sparser set of (less correlated) of predictors. Specifically, variant weights (*w*_*k*_) were derived using elastic net with the R package glmnet with *α* = 0.5. These weights are available from http://hakyimlab.org/predictdb/. Using Eq. 2, and the reference variant predictor weights (*w*_*k,g*_), the (unobserved) genetically determined expression of each gene *g* (GReX) was estimated in our GWAS sample. For both transcriptome models, separate analyses were performed for genetically based expression of genes (up to 2 tests per gene). Genes with predictive *R*^2^ > 0.01 were tested for association with CRC in our cohort (colon transverse *n* = 4850 genes, and whole blood *n* = 8277 genes).

### Gene level tests of CRC association with imputed genetically regulated gene expression

#### Discovery phase

Statistical analyses of all data were conducted centrally at the GECCO coordinating center on individual-level data. Multivariate logistic regression models were adjusted for age, sex (when appropriate), center (when appropriate), and genotyping batch (ASTERISK) and the first four principal components to account for potential population substructure. Imputed genetically regulated gene expression (GREx), was treated as a continuous variable. All studies were analyzed together in a pooled dataset using logistic regression models to obtain odds ratios (ORs) and 95% confidence intervals (CIs). Quantile–quantile (*Q*–*Q*) plots were assessed to determine whether the distribution of the *P* was consistent with the null distribution (except for the extreme tail). All analyses were conducted using the R software (Version 3.0.1). Novelty of a gene finding was determined by taking all variant predictors of the gene and determining if they were in linkage disequilibrium (LD ≥ 0.2 in Phase 3 Thousand Genomes Europeans) with a previously reported GWAS index variant.

3 $$\begin{aligned} {\text{logit}}({p_{{\text{CRC}}}})&={\beta _0}+~{\beta _1}{\text{GReX}}~+{\beta _2}{\text{age}}+~{\beta _2}~{\text{sex}}+{\beta _3}~{\text{center}}\\&\quad +~{\beta _4}~{\text{batch}}~+{\text{PC}}1+{\text{PC}}2+{\text{PC}}3+{\text{PC}}4.\end{aligned}$$

We identified suggestive findings in the discovery stage to be replicated in a second independent dataset. In the discovery stage we employed a false-discovery rate (FDR) threshold of 0.2 separately for colon transverse and whole blood models. FDR for each gene was calculated using the R statistical package p.adjust, which uses the method of Benjamini and Hochberg to calculate the expected proportion of false discoveries amongst the rejected hypotheses (Hochberg and Benjamini 1990). Genes meeting this threshold were carried forward for replication.

#### Replication phase

To replicate novel PrediXcan findings (*n* = 3 genes from colon transverse models) that had a FDR ≤ 0.2, we used the same GTEx colon transverse, elastic net prediction models (as we had done in the discovery GECCO-CCFR data) to impute genetically regulated gene expression in replication samples from (1) CORECT (pooled across consortium studies), (2) UK Biobank and (3) a pooled dataset of 5 independent GWAS datasets. Multivariate logistic regression was used to test the association of imputed genetically regulated gene expression with colorectal cancer risk in these three datasets and then meta-analyzed effects using inverse variance weighting of *Z* scores (details provided in Online Resource 1). A two-sided *P* value less than 0.05/(number of genes to be replicated) was considered statistically significant.

### Definition of CRC risk regions and refinement of GWAS loci

The 56 previously reported CRC risk variants used in this analysis had an LD *r*^2^ ≤ 0.2 with other risk variants in our known list, or were otherwise previously reported to maintain statistical significance in regression models conditioning on other nearby risk variants (referred to hereon as ‘independent’ risk variants). For each of the 56 independent risk variants defined in Table S3, we further defined ‘risk regions’ as 1 megabase (Mb) upstream and 1 Mb downstream of each risk variant (2 Mb regions surrounding each risk variant). Overlapping 2 Mb risk regions were then combined into a single new risk region defined as the minimum and maximum chromosomal coordinates from one or more overlapping risk regions (the union of the overlapping regions). This resulted in a total of 44 regions harboring one or more risk variants (maximum of four independent risk variants). A list of transcription start sites (TSS) for genes that showed nominal association (*P* ≤ 0.05) between genetically regulated gene expression and CRC risk in colon transverse and whole blood models was then intersected with the list of 44 risk regions to identify a list a putative target genes regulated by non-coding GWAS risk variants.

### Bioinformatic follow-up

Bioinformatic follow-up was performed for the *TRIM4* and *PYGL* loci using the UCSC Genome Browser and publicly available functional data for CRC relevant tissues and cell-types from Roadmap, ENCODE, as well as previously published epigenomes (Akhtar-Zaidi et al. 2012). The *TRIM4* and *PYGL* loci were defined as the genomic region containing all variants in LD (*r*^2^ ≥ 0.2 from Phase 3 Thousand Genomes Project) with the variant predictor having the strongest marginal CRC association (*TRIM4-*rs2527886 and *PYGL*-rs12589665). We then aligned the locus with refseq protein coding genes, epigenetic signals in normal crypts and CRC cell lines to identify recurrently gained and lost variant enhancer loci (VEL), and ChIP-seq transcription factor binding sites.

## URLs

PrediXcan software, https://github.com/hakyimlab/PrediXcan; University of Michigan Imputation-Server, https://imputationserver.sph.umich.edu/start.html; GTEx Portal, http://www.gtexportal.org/; PredictDB, http://predictdb.org/.

## Electronic supplementary material

Below is the link to the electronic supplementary material.

Supplementary material 1 (DOCX 1347 KB)

Supplementary material 2 (PDF 258 KB)

Supplementary material 3 (TIFF 4218 KB)

Supplementary material 4 (TIFF 1875 KB)

Supplementary material 5 (TIFF 4218 KB)

Supplementary material 6 (TIFF 1875 KB)
