# Supplementary material for: Genetic variant predictors of gene expression provide new insight into risk of colorectal cancer
Source: Hum Genet. 2019 Feb 28;138(4):307–26. doi: 10.1007/s00439-019-01989-8 (PMC6483948; doi:10.1007/s00439-019-01989-8)
Supplement: Supplementary file 1 — Supplementary material 1 (DOCX 1347 KB) [file 439_2019_1989_MOESM1_ESM.docx]

**Supplemental Tables and Figures**

**Table S1. CRC association results and predictive R^2^ for imputed expression of all genes.** Genes within 1Mb of a known CRC GWAS variant are highlighted in red. Genes reaching False Discovery Rate (FDR) = 0.2 not in a known region are highlighted in blue. Genes are sorted by chromosome and then by *P*.

# **Table S2. Descriptive Characteristics of Study**

|  | **Case** | **Control** |
| --- | --- | --- |
| **DISCOVERY – GECCO Consortium** | | |
| n | 12,186 | 14,718 |
| Age (median [IQR]) | 64 [56, 70] | 65 [58, 70] |
| Female (%) | 6522 (53.5) | 7940 (53.9) |
| **GWAS platform (%)** |  |  |
| Affy Chips | 602 (4.9) | 522 (3.5) |
| CytoSNP | 5484 (45.0) | 5424 (36.9) |
| Human1M/1Mduo | 1569 (12.9) | 1393 (9.5) |
| Initial | 1712 (14.0) | 4212 (28.6) |
| OmniExpress | 2819 (23.1) | 3167 (21.5) |
| **REPLICATION – CORECT Consortium** | | |
| n | 22,974 | 14,392 |
| Age (mean (sd)) | 63 (11) | 64 (11) |
| Male (%) | 12,850 (56) | 7,098 (49) |
| **Ancestry** |  |  |
| Asian | 3026 (13.2) | 2268 (15.8) |
| European | 19,948 (86.8) | 12,124 (84.2) |
| **GWAS platform (%)** |  |  |
| Axiom | 4582 (19.9) | 4067 (28.3) |
| MECC_Omni | 483 (2.1) | 495 (3.4) |
| OncoArray | 17909 (78.0) | 9830 (68.3) |
| **REPLICATION – UK Biobank** |  |  |
| n | 5,356 | 21,407 |
| Age (mean (sd)) | 63.00 [58.00, 66.00] | 61.00 [56.00, 65.00] |
| Female (%) | 2,275 (42.5) | 9,084 (42.4) |
| **GWAS platform (%)** |  |  |
| Affymetrix UK Biobank Axiom | 5,356 (100) | 21,407 (100) |
| **REPLICATION – Additional GWAS** |  |  |
| n | 4,439 | 4,115 |
| Age (mean (sd)) | 62.00 [56.00, 71.00] | 61.00 [54.00, 67.00] |
| Female (%) | 2,115 (47.6) | 2,033 (49.4) |
| **GWAS platform (%)** |  |  |
| HumanOmniExpressExome-8v1-2 | 4,439 (100%) | 4,115 (100%) |

**Table S3. Definitions for Known CRC Regions**

| **Cytogenic band** | **Region coordinates**  **(Total = 45)*** | **Previously Purported Genes** | **Independent**  **(r^2^≤ 0.2) CRC Risk Variants (Total = 56)** | **Index First Author(s)** | **Risk Variants + LD Variants Correlated with Risk Variants (r^2^≥ 0.2)** | **Number of LD Variants used in a Prediction Model** | |
| --- | --- | --- | --- | --- | --- | --- | --- |
|  |  |  |  |  |  | **CT** | **WB** |
| 1p36.12 | 21587728- 23587728 | *WNT4, CDC42* | rs72647484 | Al-Tassan | 76 | 3 | 2 |
| 1q25.3 | 182081194-184081194 | *LAMC1* | rs10911251 | Whiffin | 548 | 0 | 25 |
| 1q41 | 221045446-223045446 | *DUSP10* | rs6691170 | Houlston | 170 | 2 | 7 |
| 2q32.3 | 191587204-193587204 | *NABP1* | rs11903757 | Peters | 65 | 0 | 0 |
| 2q35 | 218154781-220154781 | *PNKD, TMBIM1* | rs992157 | Orlando | 358 | 12 | 69 |
| 3p22.1 | 39924962-41924962 | *CTNNB1* | rs35360328 | Schumacher | 71 | 0 | 1 |
| 3p14.1 | 65442435-67442435 | *LRIG1* | rs812481 | Schumacher | 138 | 3 | 9 |
| 3q26.2 | 168492101- 170950156 | *TERC, MYNN, PRKC1* | rs10936599, rs185423955 | Houlston, Schmit | 277 | 5 | 0 |
| 4q31.1 | 148748994-150748994 | *NR3C2* | rs60745952 | Markowitz | 111 | 0 | 0 |
| 4q32.2 | 162333405-164333405 | *FSTL5* | rs35509282 | Schmit | 109 | 0 | 0 |
| 5p15.33 | 286516-2286516 | *TERT* | rs2736100 | Kinnersley | 26 | 1 | 2 |
| 5q22.2 | 111175211-113175211 | *APC* | rs1801155 | Niell, Peters, Boursi | 1 | 0 | 0 |
| 5q31.1 | 133499092-135499092 | *PITX1, H2AFY* | rs647161 | Jia | 114 | 1 | 2 |
| 6p21.2 | 35622900-37622900 | *CDKN1A* | rs1321311 | Dunlop | 75 | 1 | 8 |
| 6p21.1 | 40692812-42692812 | *TFEB* | rs4711689 | Zeng | 27 | 1 | 0 |
| 6q22.1 | 116822993-118822993 | *DCDBL2* | rs4946260 | Schumacher | 203 | 11 | 25 |
| 6q25.3 | 159840252-161840252 | *SCL22A3* | rs7758229 | Cui | 105 | 0 | 13 |
| 8q23.3 | 116624093- 118630683 | *EIF3H* | rs16892766, rs2450115 | Tomlinson, Zeng | 183 | 1 | 8 |
| 8q24.21 | 127413305-129413305 | *MYC* | rs6983267 | Tomlinson | 57 | 1 | 4 |
| 9q24 | 5365683-7365683 | *TPD52L3* | rs719725 | Zanke | 187 | 6 | 3 |
| 10p14 | 7701219-9701219 | *GATA3* | rs10795668 | Tomlinson | 116 | 0 | 2 |
| 10q22.3 | 79819132-81819132 | *ZMIZ1,AS1, POLR3A* | rs704017 | Zhang | 26 | 1 | 0 |
| 10q24.2 | 100345366-102345366 | *SnoU13,COX15,CNNM1,WNT8B* | rs1035209 | Whiffin | 102 | 3 | 7 |
| 10q24.32 | 103595248-105595248 | *CYP17A1* | rs4919687 | Zeng | 372 | 10 | 32 |
| 10q25.2 | 113280702- 115726843 | *TCF7L2,VTI1A* | rs11196172, rs12241008 | Zhang, Wang | 39 | 5 | 3 |
| 11q12.2 | 60552680- 62982418 | *MYRF, FEN1, FADS1* | rs174537, rs60892987 | Zhang, Schmit | 268 | 9 | 53 |
| 11q13.4 | 73345550-75345550 | *POLD3* | rs3824999 | Dunlop | 262 | 9 | 14 |
| 11q23.1 | 110171709-112171709 | *C11orf53, COLCA1, COLCA2* | rs3802842 | Tenesa | 113 | 24 | 5 |
| 12p13.32 | 3368352- 7982162 | *CCND2, SPSB2, CD9* | rs10774214, rs3217810, rs11064437, rs10849432 | Jia, Whiffin, Zeng, Zhang | 181 | 11 | 11 |
| 12q13.12 | 50155663-52155663 | *DIP2B,ATF1* | rs11169552 | Houlston | 376 | 19 | 26 |
| 12q24.12 | 110884608-112884608 | *SH2B3* | rs3184504 | Schumacher | 204 | 3 | 27 |
| 12q24.22 | 116747590-118747590 | *NOS1* | rs73208120 | Schumacher | 119 | 5 | 2 |
| 14q22.2 | 53410919- 55560018 | *BMP4* | rs4444235, rs1957636 | Houlston, Tomlinson | 422 | 2 | 13 |
| 15q13.3 | 31993111- 34004247 | *GREM1* | rs11632715, rs16969681 | Tomlinson | 13 | 0 | 2 |
| 16p13.2 | 8297812-10297812 | *C16orf72* | rs79900961 | Al-Tassan | 4 | 0 | 0 |
| 16q22.1 | 67820946-69820946 | *CDH1* | rs9929218 | Houlston | 589 | 6 | 73 |
| 16q24.1 | 85695720-87695720 | *FOXL1* | rs16941835 | Al-Tassan | 113 | 4 | 0 |
| 17p13.3 | 0-1800593 | *NXN* | rs12603526 | Zhang | 93 | 0 | 7 |
| 18q21.1 | 45450976- 47453463 | *SMAD7* | rs4939827, rs7229639 | Broderick,Zhang | 44 | 2 | 6 |
| 19q13.11 | 32532300-34532300 | *RHPN2, GPATCH1* | rs10411210 | Houlston | 234 | 1 | 14 |
| 19q13.2 | 40860296-42860296 | *TGFB1, B9D2* | rs1800469 | Zhang | 191 | 21 | 37 |
| 20p12.3 | 5404281- 8812350 | *BMP2, HAO1* | rs961253, rs4813802, rs2423279 | Houlston, Tomlinson, Jia | 668 | 3 | 37 |
| 20q13.13 | 46340117-48340117 | *PREX1* | rs6066825 | Schumacher | 166 | 1 | 1 |
| 20q13.33 | 59921044-61921044 | *LAMA5, RPS21* | rs4925386 | Houlston | 201 | 8 | 3 |

## ^A^ For all 56 independent CRC risk SNPs, regions defined as 1 megabase (Mb) upstream and 1Mb downstream (total of 2Mb for each SNP). Overlapping regions were combined into a new region defined as the minimum and maximum coordinate range (the union of the overlapping regions) for a total of 44 CRC risk regions harboring one or more CRC risk variants.

**
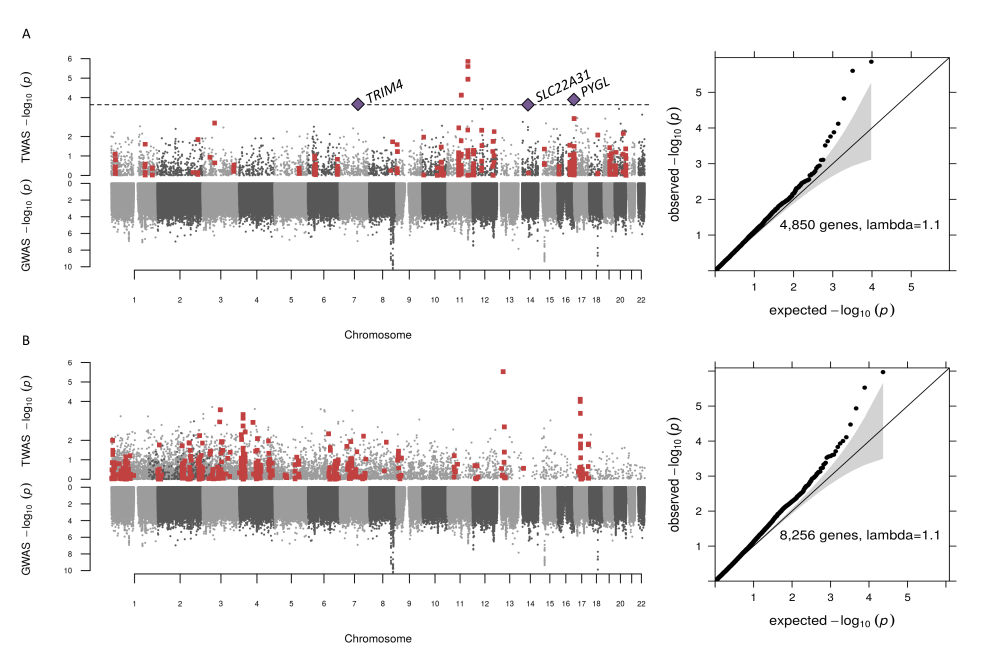
**

**Fig S1. Results for the association of predicted gene expression using colon transverse and whole blood models with CRC risk in the discovery phase.** CRC association results -log10(*p*), are shown for genes imputed using A) colon transverse and, B) whole blood models. On the left, Manhattan plots are shown for transcriptome-wide association (TWAS) results on the top and genome-wide association results for single variants reflected on the bottom (i.e. Miami plots). In the TWAS plots, gene-CRC association *P* (y-axis) are plotted against the chromosomal position for the transcription start site (TSS) of the gene. Genes with TSS that are within 1Mb of a known GWAS index variant for CRC are colored red, and novel significant genes are highlighted as blue diamonds. The remaining gene association *p-*values are colored by chromosomes alternating between light and darker shades of gray. A dashed line on the TWAS plot divides genes surpassing the FDR threshold (0.2). Quantile–quantile (QQ) plots of CRC-gene associations are shown on the right. Points represent the -log10 of expected versus observed *p-*values, gray shading highlights the 95% confidence interval.

**Online Resource 3-6 Fig S2: Marginal CRC association results for variants used to predict expression of the replicated genes** Coding allele frequency (CAF), single variant association *p*-value, and LD structure for each variant predictor of gene expression in colon transverse models are shown for *TRIM4* (S2a, S2b) and *PYGL* (S2c, S2d). S2a and S2c show CAF for each variant predictor presented as a horizontal bar chart on the left panel. On the right panel of S2a and S2c we show the CRC-variant association for the corresponding variant predictors with odds ratio (points), associated 95% confidence intervals (horizontal lines), and p-values written on left for variants with p<0.05. Non-significant variants are in light gray. Pairwise LD structure for the variant predictors are shown in S2b and S2d and colored by their Pearson correlation r.

**
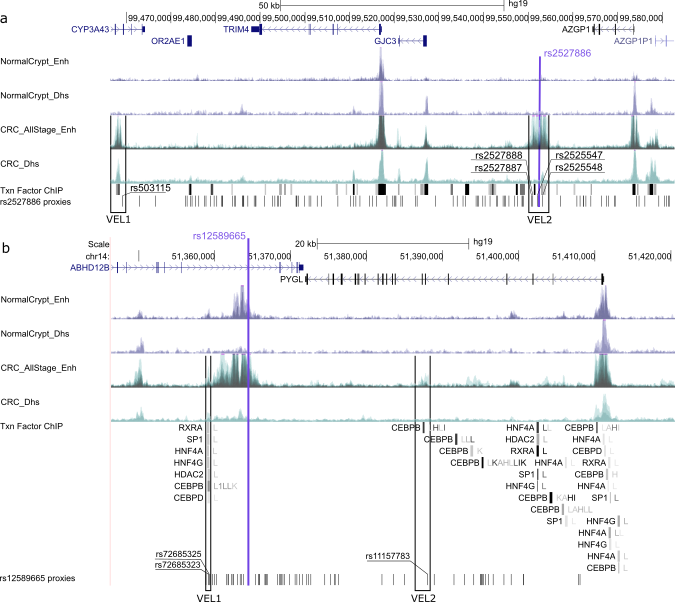
**

**Fig S3 Bioinformatic Functional Follow-up of *TRIM4* and *PYGL* loci** UCSC genome browser images are shown for the a) *TRIM4* (chr7:99,456,448-99,582,701) and b) *PYGL* loci. Browser images display 7 tracks: Refseq Protein coding genes, four transparent overlay tracks for epigenetic signals in normal crypts and cancer cell lines from Scacheri et al. (NormalCrypt_Enh, ChIP-seq enhancers in 4 normal crypts; NormalCrypt_Dhs, DNA accessibility in 3 normal crypts; CRC_AllStage_Enh, ChIP-seq enhancers in 31 CRC cell lines across different stages; and CRC_Dhs, DNA accessibility in 3 CRC cell lines; respectively). The transparent overlay tracks are followed by transcription factor ChIP-seq binding sites and the last track shows all variants in LD (r^2^≥0.5) with the most significantly CRC associated variant predictor for the corresponding gene. Regions span the LD variant proxies. Position of the most significant variant predictors are highlighted by the purple line. S3a) The variant predictor for *TRIM4* with strongest marginal significance was rs2527886, and falls within one of two gained variant enhancer loci (VEL), which are variant enhancer elements with gained or lost ChIP-seq enhancer activity in CRC cell lines compared to normal crypts. S3b) The most significant variant from univariate analysis was rs12589665 and three variants in LD with rs12589665 were positioned in VEL, of which rs72685325 and rs72685323 were in transcription factor binding sites.

*Exploration of potential inflated QQ plot in whole blood*


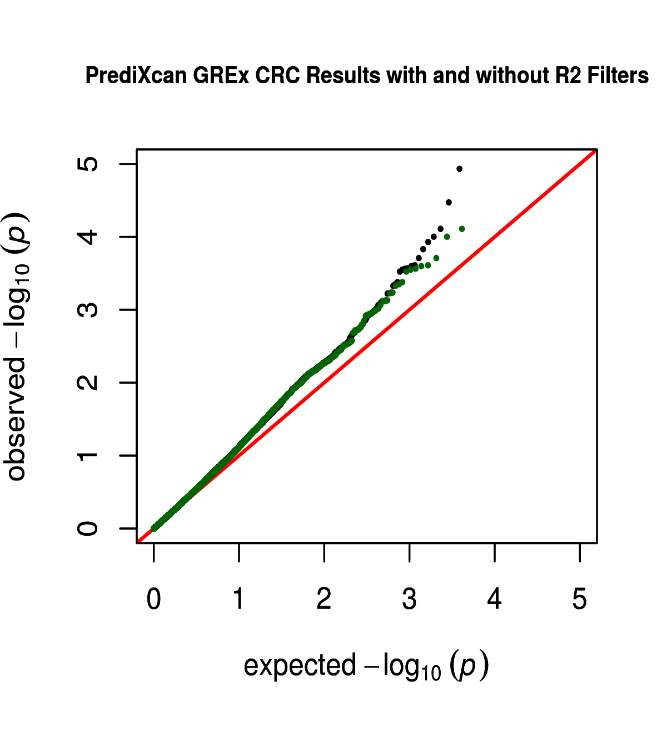
Before filtering on predictive R^2^>0.1 we observed an inflation factor of 1.11 for our PrediXcan analysis of DGN whole blood imputed gene expression (GREx) and CRC risk. The following work is a summary of the series of exploratory analyses conducted to assess whether this inflation was the result of potential type 1 error as a result of bias in our data or modeling error or likely to be true signals. First, Gamazon et al. [(42)](http://f1000.com/work/citation?ids=756354&pre=&suf=&sa=0) recommends that association analyses be restricted to genes with narrow sense heritability, *h^2^*, greater than 0.1 (congruent to predictive R^2^>0.1). Thus, as an initial QC step we filtered out genes with low heritability and this resulted in a slight reduction of inflation in the QQ-plot (**Fig S5**), although the lambda was unchanged.

**Fig S4. Quantile-quantile plot of the association *p*-values from the PrediXcan analysis of CRC using expression levels imputed from DGN whole blood and stratified by gene heritability.**

Quantile–quantile (QQ) plots of CRC results from the logistic regression model with covariates age, study, sex, and four principal components of genetic ancestry. The red line shows the expected distribution of *p*-values for GREx association. Predictive R*^2^*, was used to filter out genes with low predictive accuracy and those with R*^2^* ≤ 0.01 were excluded from further analysis. There were 11,578 genes before filtering and 8,277 genes with R *^2^ ≥* 0.1 used for this analysis (shown in green). The inflation factor was the same (λ=1.10) before and after filtering on predictive R*^2^*.

We then investigated the regression covariates and potential differences in genotyping phases between studies. To do so we added in an interaction term with each covariate (see model 1 below). The inflation factor for GREx results from the interaction model was slightly higher (λ=1.12) suggesting that the imputation of data in different genotyping phases was not a likely cause of bias (**Fig S6**). To further explore this, we ran 9 simulations using model 1 and permuted case/control status within each genotyping phase. All QQ-plots from the simulations followed a uniform distribution and no inflation was detected (lambdas ranging from 0.94-1.03, **Fig S7**). Additionally, we investigated whether the use of PCs calculated by GWAS variant data as opposed to PCs of GREx led to unaccounted batch effects between studies. After including the first four principal components of GREx for all tested genes we again observed a similar inflation pattern (**Fig S8**).

Model 1: $case \sim GReX+ {age}_{ref}+sex+study+PC1+PC2+PC3+PC4+ {age}_{ref}*genotype phase+sex*genotype phase+PC1* genotype phase+PC2*genotype phase+PC3*genotype phase+PC4*genotype phase$


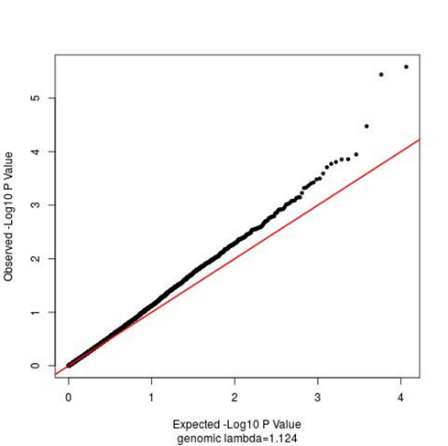
**Fig S5. Quantile-quantile plot of the association *p-*values from the PrediXcan analysis of CRC using expression levels imputed from DGN whole blood and models including genotyping phase interaction with all covariates.** Quantile–quantile (QQ) plots of CRC from the logistic regression model with covariates age, study, sex, four principal components of genetic ancestry, and interaction with genotyping phase for each covariate (model 1). Points represent the negative log10 of expected versus observed *p*-values. A genomic inflation factor of 1.12 was observed.


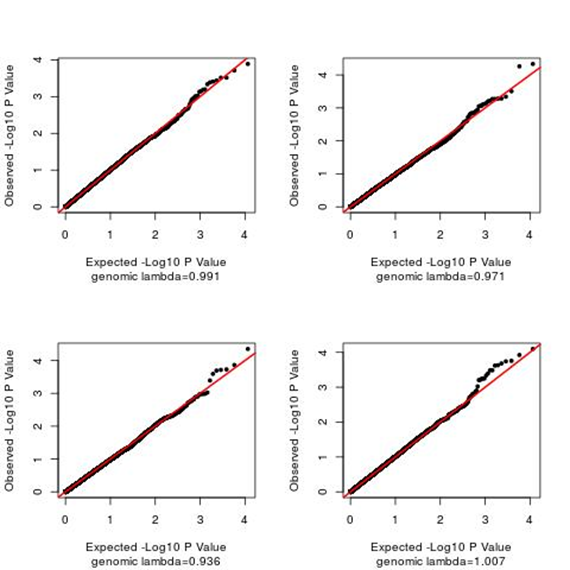

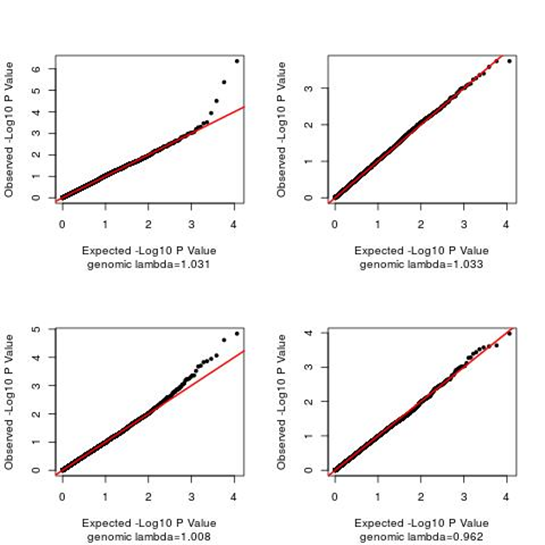


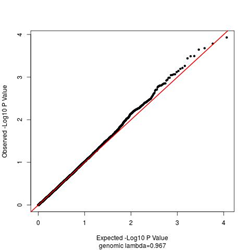
**Fig S6. Quantile-quantile plots of the association *p-*values from the PrediXcan analysis of CRC using expression levels imputed from DGN whole blood with permuted case status.**

Quantile–quantile (QQ) plots of CRC from the logistic regression model with covariates age, study, sex, four principal components of genetic ancestry, and interaction terms with genotyping phase for each covariate. In each simulation, case status was randomized within genotyping phase. Points represent the negative log10 of expected versus observed *p*-values. The inflation factor ranged from 0.94 to 1.01.


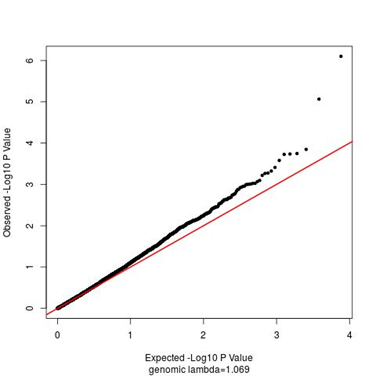
**Fig S7: Quantile-quantile plots of the association *p*-values from the PrediXcan analysis of CRC using expression levels imputed from DGN whole blood and adjusting for principal components of genetically regulated expression.** Quantile–quantile (QQ) plots of CRC from the logistic regression model with covariates age, study, sex, four principal components of GREx. Points represent the negative log10 of expected versus observed *p*-values. The inflation factor was similar to base model (λ=1.07).

Next, although the filter for predictive R^2^ did not reduce the inflation factor, we explored whether the variance of GREx for some genes may have been very small in our data, which in turn could have caused inflation in the z-statistics. To explore this, we filtered out genes with a low standard deviation (SD ≤ 0.03) in GREx and again we observed a similar inflation in the CRC association results for the remaining 6,892 genes (**Fig S9 and S10**). Furthermore, when GREx was divided into different variance strata no relationship between *p*-values and SD strata was observed (**Fig S11 and S12**).


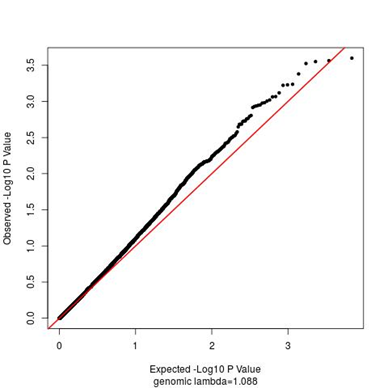
**Fig S8 : Quantile-quantile plots of the association *p-*values from the PrediXcan analysis of CRC using expression levels imputed from DGN whole blood filtering on variance of expression (n=6,892)** Quantile–quantile (QQ) plots of CRC from the logistic regression model with covariates age, study, sex, and four principal components of GREx. GREx with standard deviation less than 0.3 were removed. Points represent the negative log10 of expected versus observed *p*-values. The inflation factor was similar to base model (λ=1.088).


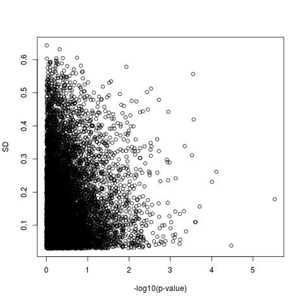

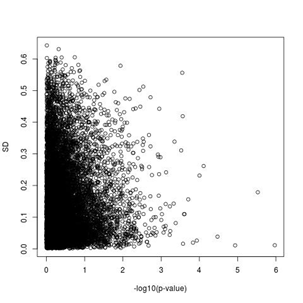
**Fig S9: Scatter plots of standard deviations of expression levels versus CRC association *p*-values imputed from DGN whole blood (GREx) before and after variance filtering.** Scatter plots of GREx scores versus CRC association *p*-values from the logistic regression model with covariates age, study, sex, and four principal components of GREx. The plot on the left shows all genes and the plot of the right shows results for genes with appreciable variance in imputed expression (GREx SD>0.03).


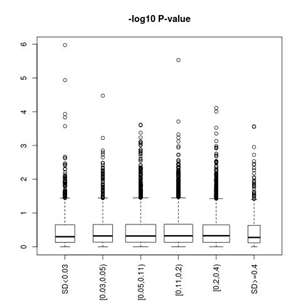

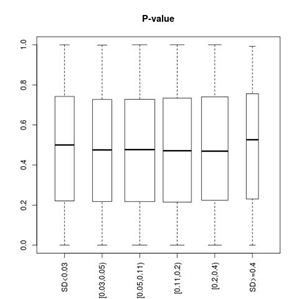


**Fig S10: Distribution of CRC association *p*-values for expression levels imputed from DGN whole blood in different variance strata.** Box plots for the distribution of CRC association *p*-values from the logistic regression model with covariates age, study, sex, and four principal components of GREx are shown for different strata of GREx standard deviation. The -log10 *p*-value is shown on the left and the untransformed *p-*values is shown on right.


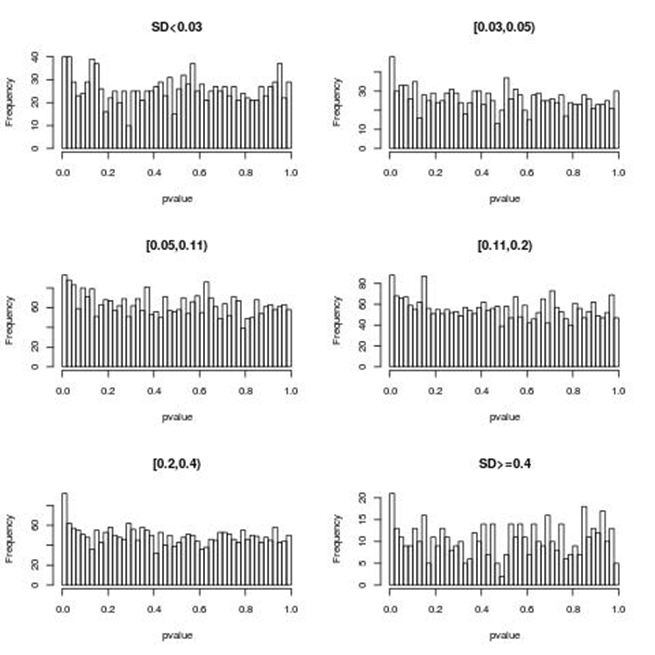
**Fig S11: Histograms of CRC association *p*-values for expression levels imputed from DGN whole blood in different variance strata.** Histograms for the distribution of CRC association *p*-values from the logistic regression model with covariates age, study, sex, and four principal components of GREx are shown for different strata of GREx standard deviation.

After confirming the association test model, we further explored the imputed expression data. Single variant marginal CRC association results for the variants used to derive GREx were examined through a QQ-plot and inflation for the 248,774 variants was observed (λ=1.07, **Fig 13**). The observation of inflation of association signal for the variant predictors used to derive the GREx further supports the likelihood that the inflation observed in our PrediXcan analysis is reflective to true associations with CRC.


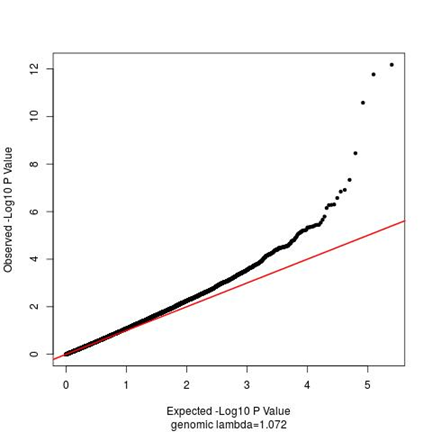
**Fig S12: Quantile-quantile plot for individual variant association results for DGN whole blood variant predictors used to impute gene expression levels (n=248,774 variants).** Quantile–quantile (QQ) plots of CRC from the logistic regression model with covariates age, study, sex, and four principal components of ancestry. Points represent the -log10 of expected versus observed *p*-values. The inflation factor was elevated in the variant predictors (λ=1.072) that were used to imputed gene expression used in the PrediXcan analysis.

Finally, to further investigate whether inflation was a result of confounding, we performed the following analysis. First we calculated pairwise correlation between genes of model 2 residuals. Then, among correlated gene pairs (|r|>0.7) we used a priority pruning method to remove the gene with less significant (larger) *p*-value. Next, residual PCs for Model 2 were recalculated and Eigenvalues were plotted. Based on the Eigenvalue plot, 10 residual PCs were chosen and the PrediXcan analysis was rerun using Model 3. In this analysis, genes found to be highly co-expressed were ‘pruned’ so that correlation would not be a likely cause of inflation. However, the inflation factor remained elevated (Fig S20, λ=1.05). In conclusion, after thorough investigation we cannot rule out that the inflation observed in our PrediXcan results is due to true signal and reflects the polygenicity of CRC.

Model 2: $GReX\sim{age}_{ref}+sex+study+genotype phase+PC1+PC2+PC3+PC4$

Model 3: $case\sim GReX+{age}_{ref}+sex+study+genotype phase+PC1+PC2+PC3+PC4+residual PC1+residual PC2+residual PC3+residual PC4$


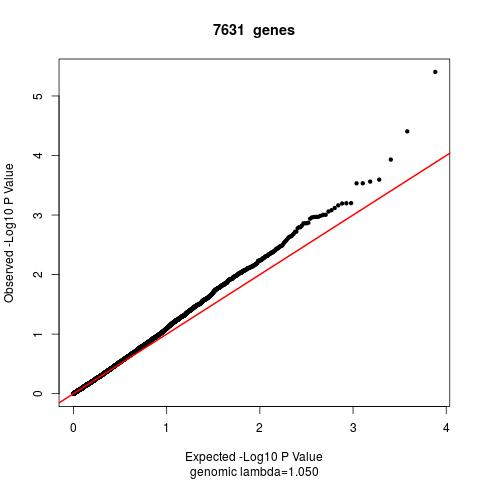


**Fig S13: Quantile-quantile plots of the association *p-*values from the PrediXcan analysis of CRC using expression levels imputed from DGN whole blood after pruning highly correlated genes.** Quantile–quantile (QQ) plots of CRC from the logistic regression model with covariates age, study, sex, and four principal components of ancestry and 10 principal components of model residuals. Highly coexpressed genes (|r|<0.7) were pruned to remove the gene with less significant CRC-association (large *p*-value). Points represent the -log10 of expected versus observed *p*-values. The inflation factor (λ=1.05) remained elevated.

# **Materials and Methods Description of Study cohort**

*Discovery Phase: Genetics and Epidemiology of Colorectal Cancer Consortium (GECCO) and Colon Cancer Family Registry (CCFR)*

***The french Association STudy Evaluating RISK for sporadic colorectal cancer (ASTERISK)*** (1). Participants were recruited from the Pays de la Loire region in France between December 2002 and March 2006. Eligibility criteria for cases included being of Caucasian origin, being greater than or 40 years of age at diagnosis, and having no family history of colorectal cancer or polyps. Cases were patients with first primary colorectal cancer diagnosed in one of the six public hospitals and five clinics located in the Pays de la Loire region which participated in the study. Cases were confirmed based on medical and pathology reports. Controls were recruited at two Health Examination Centers of the Pays de la Loire region, and the recruitment of controls greater than or 70 years was completed in the departments of internal medicine and hepatogastroenterology of the University Hospital Center of Nantes, located in the same region. Controls were eligible to participate if they were Caucasian, aged greater than or 40 years, and had no family history of colorectal cancer or polyps. In the presence of the physician, each participant filled out a standardized questionnaire on family information, medical history, lifestyle, and dietary intake. Cases and controls provided a blood sample.

***Darmkrebs: Chancen der Verhütung durch Screening (DACHS1, DACHS2)* (2,3)***.* This German study was initiated as a large population-based case-control study in 2003 in the Rhine-Neckar-Odenwald region (southwest region of Germany) to assess the potential of endoscopic screening for reduction of CRC risk and to investigate etiologic determinants of disease, particularly lifestyle/environmental factors and genetic factors. During an in-person interview, data were collected on demographics, medical history, family history of CRC, and various life-style factors, as were blood and mouthwash samples. Cases with a first diagnosis of invasive CRC (International Classification of Diseases 10 codes C18-C20) who were at least 30 years of age (no upper age limit), German speaking, a resident in the study region, and mentally and physically able to participate in a one-hour interview, were recruited by their treating physicians either in the hospital a few days after surgery, or by mail after discharge from the hospital. Cases were confirmed based on histologic reports and hospital discharge letters following diagnosis of CRC. All hospitals treating CRC cancer patients in the study region participated. Community-based controls were randomly selected from population registries, employing frequency matching with respect to age (5-year groups), sex, and county of residence. Controls with a history of CRC were excluded. Controls were contacted by mail and follow-up calls. The datasets analyzed in the discovery study consist of 2,376 cases and 2,205 controls genotyped using CytoSNP and OmniExpress.

***Diet, Activity, and Lifestyle Study (DALS)*** *(4)*. DALS is a population-based case-control study of colon cancer. Participants were recruited between 1991 and 1994 from three locations: the Kaiser Permanente Medical Care Program (KPMCP) of Northern California, an eight-county area in Utah, and the metropolitan Twin Cities area of Minnesota. Eligibility criteria for cases included age at diagnosis between 30 and 79 years, diagnosed with first primary colon cancer (International Classification of Diseases for Oncology-2 codes 18.0 and 18.2-18.9) between October 1st 1991 and September 30th 1994, English speaking, and competency to complete the interview. Individuals with cancer of the rectosigmoid junction or rectum were excluded, as were those with a pathology report noting familial adenomatous polyposis, Crohn’s disease, or ulcerative colitis. A rapid-reporting system was used to identify all incident cases of colon cancer resulting in the majority of cases being interviewed within four months of diagnosis. Controls from KPMCP were randomly selected from membership lists. In Utah, controls under 65 years of age were randomly selected through random-digit dialing and driver license lists. Controls, 65 years of age and older, were randomly selected from Health Care Financing Administration lists. In Minnesota, controls were identified from Minnesota driver’s license or state ID lists. Controls were matched to cases by 5-year age groups and sex. The Set I scan consisted of a subset of the study designed above, from Utah, Minnesota, and KPMCP, and was restricted to subjects who self-reported as White non-Hispanic. The Set 2 scan consisted of subjects from Utah and Minnesota that were not genotyped in Set 1. Set 2 was restricted to subjects who self-reported as White non-Hispanic and those that had appropriate consent to post data to dbGaP.
Hawai’i Colorectal Cancer Studies 2 & 3 (Colo2&3) (5). Patients with colorectal cancer were identified through the rapid reporting system of the Hawaii SEER registry and consisted of all Japanese, Caucasian, and Native Hawaiian residents of Oahu who were newly diagnosed with an adenocarcinoma of the colon or rectum between January 1994 and August 1998. Control subjects were selected from participants in an on-going population-based health survey conducted by the Hawaii State Department of Health and from Health Care Financing Administration participants. Controls were matched to cases by sex, ethnicity, and age (within two years). Personal interviews were obtained from 768 matched pairs, resulting in a participation rate of 58.2% for cases and 53.2% for controls. A questionnaire, administered during an in-person interview, included questions about demographics, lifetime history of tobacco, alcohol use, aspirin use, physical activity, personal medical history, family history of colorectal cancer, height and weight, diet (Food Frequency Questionnaire), and postmenopausal hormone use. A blood sample was obtained from 548 (71%) of interviewed cases and 662 (86%) of interviewed controls. SEER staging information was extracted from the Hawaii Tumor Registry. In GECCO, self-reported Caucasian subjects with DNA, and clinical and epidemiologic data were selected for genotyping.

***Health Professionals Follow-up Study (HPFS)*** (6). HPFS is a parallel prospective study to the NHS. The HPFS cohort comprised 51,529 men aged 40-75 who, in 1986, responded to a mailed questionnaire. Participants provided information on health-related exposures, including current and past smoking history, age, weight, height, diet, physical activity, aspirin use, and family history of colorectal cancer. Colorectal cancer and other outcomes were reported by participants or next-of-kin and were followed up through review of the medical and pathology record by physicians. Overall, more than 97% of self-reported colorectal cancers were confirmed by medical record review. Information was abstracted on histology and primary location. Follow-up evaluation has been excellent, with 94% of the men responding to date. In 1993-1995, 18,825 men in the HPFS mailed blood samples by overnight courier, which were aliquoted into buffy coat and stored in liquid nitrogen. In 2001-2004, 13,956 men in the HPFS who had not provided a blood sample previously, mailed in a swish-and-spit sample of buccal cells. Incident cases were defined as those occurring after the subject provided a blood or buccal sample. Prevalent cases were defined as those occurring after enrollment in the study in 1986, but before the subject provided either a blood or buccal sample. Sample selection of the discovery stage case-control sets has been described in detail previously [1,2]. For the replication set (HPFS3), colorectal cancer cases were ascertained through January 1, 2010 and excluded cases included in the discovery stage [2]. Participants with histories of cancer (except nonmelanoma skin cancer), ulcerative colitis, or familial polyposis were excluded. CRC cases matched to randomly selected controls who provided a blood or buccal sample and were free of colorectal cancer at the same time the colorectal cancer was diagnosed in the cases. Matching criteria included year of birth (within 1 year) and month/year of blood or buccal cell sampling (within 1 year). If no control could be matched for a case using the initial stringent criteria, age criteria were relaxed to <5 years to find an eligible control.

***Multiethnic Cohort Study (MEC)*** *(7)*. MEC was initiated in 1993 to investigate the impact of dietary and environmental factors on major chronic diseases, particularly cancer, in ethnically diverse populations in Hawai’i and California. The study recruited 96,810 men and 118,441 women aged 45 to 75 years between 1993 and 1996. Incident colorectal cancer cases occurring since January 1995, and controls were contacted for blood or saliva samples. The median interval between diagnosis and blood draw was 14 months (interquartile range, 10-19) among cases and the participation rate 74%. A sample of cohort participants was randomly selected to serve as controls at the onset of the nested case-control study (participation rate 66%). The selection was stratified by sex, age, and race/ethnicity. Colorectal cancer cases are identified through the Rapid Reporting System of the Hawai’i Tumor Registry and through quarterly linkage to the Los Angeles County Cancer Surveillance Program. Both registries are members of SEER. In GECCO, self-reported White subjects from the nested case-control study described above with DNA, and clinical and epidemiologic data were selected for genotyping.

***Nurses’ Health Study (NHS)*** *(8)*. The NHS cohort began in 1976 when 121,700 married female registered nurses aged 30 to 55 years returned the initial questionnaire that ascertained a variety of important health-related exposures. Since 1976, follow-up questionnaires have been mailed every two years. Colorectal cancer and other outcomes were reported by participants or next-of-kin and followed up through review of the medical and pathology record by physicians. Overall, more than 97% of self-reported colorectal cancers were confirmed by medical-record review. Information was abstracted on histology and primary location. Follow-up has been high: as a proportion of the total possible follow-up time, follow-up has been over 92%. Colorectal cancer cases were ascertained through June 1, 2008. In 1989-90, 32,826 women in NHS I, mailed in blood samples by overnight courier which were aliquoted into buffy coat and stored in liquid nitrogen. In 2001-04, 29,684 women in NHS I who did not previously provide a blood sample mailed in a "swish-and-spit" sample of buccal cells. Incident cases are defined as those occurring after the subject provided a blood or buccal sample. Prevalent cases are defined as those occurring after enrollment in the study in 1976, but prior to the subject providing either a blood or buccal sample. After excluding participants with histories of cancer (except non-melanoma skin), ulcerative colitis, or familial polyposis, two case-control sets were constructed from which DNA was isolated from either buffy coat or buccal cells for genotyping: 1) a case-control set with cases of colorectal cancer matched to randomly selected controls who provided a blood sample and were free of colorectal cancer at the same time the colorectal cancer was diagnosed in the case; 2) a case-control set with cases of colorectal cancer matched to randomly selected controls who provided a buccal sample and were free of colorectal cancer at the same time the colorectal cancer was diagnosed in the cases. For both case-control sets, matching criteria included year of birth (within one year) and month / year of blood or buccal cell sampling (within six months). Cases were pair matched 1:1, 1:2, or 1:3 with a control participant(s).
In addition to colorectal cancer cases and controls, a set of adenoma cases and matched controls with available DNA from buffy coat were selected for genotyping. Over follow-up, data were collected on endoscopic screening practices and, if individuals have been diagnosed with polyp, the polyps confirmed to be adenomatous by medical record review. Adenoma cases were ascertained through June 1, 2008. A separate case-control set was constructed of participants diagnosed with advanced adenoma matched to control participants who underwent a lower endoscopy in the same time period and did not have an adenoma. Advanced adenoma was defined as an adenoma >1 cm in diameter and / or with tubulovillous, villous, or high-grade dysplasia / carcinoma-in-situ histology. Matching criteria included year of birth (within one year) and month/year of blood sampling (within six months), the reason for their lower endoscopy (screening, family history, or symptoms) and the time period of any prior endoscopy (within two years). Controls matched to cases with a distal adenoma either had a negative sigmoidoscopy or colonoscopy exam and controls matched to cases with proximal adenoma all had a negative colonoscopy.

***Ontario Familial Colorectal Cancer Registry (OFCCR)*.** For this GECCO analysis, a subset of the Assessment of Risk in Colorectal Tumours in Canada (**ARCTIC**) from the Ontario Registry for Studies of Familial Colorectal Cancer (OFCCR) was used. Details on the case-control study (9) and the OFCCR (10), as well as the GWAS results (11) have previously been reported. In brief, cases were confirmed incident colorectal cancer (CRC) cases aged 20 to 74 years, residents of Ontario identified through comprehensive registry and diagnosed between July 1997 and June 2000. Population-based controls were randomly selected among Ontario residents (random-digit-dialing and listing of all Ontario residents), and matched by sex and 5-year age groups. A total of 1,236 CRC cases and 1,223 controls were successfully genotyped on at least one of the Illumina 1536 GoldenGate assay (Illumina, Inc, San Diego, CA), the Affymetrix GeneChip® Human Mapping 100K and 500K Array Set (Affymetrix, Inc, Santa Clara, CA), and a 10K non-synonymous SNP chip. Analysis was based on a set of unrelated subjects who were non-Hispanic, White by self-report or by investigation of genetic ancestry. Further exclusions were made for sample swaps, missing epidemiologic questionnaire data, appendix tumor, or if a subject overlapped with the Colon Cancer Family Registry. Additionally, only samples genotyped on the Affymetrix GeneChip® 500K Array were utilized in order to avoid convergence issues in imputation.

***Physician’s Health Study (PHS)*** (12,13). The PHS was established as a randomized, double-blind, placebo-controlled trial of aspirin and ß-carotene among 22,071 healthy U.S. male physicians, between 40 and 84 years of age in 1982. Participants completed two mailed questionnaires before being randomly assigned, additional questionnaires at six and 12 months, and questionnaires annually thereafter. In addition, participants were sent postcards at six months to ascertain status. From August 1982 to December 1984, 14,916 baseline blood samples were collected from the physicians during the run-in phase before randomization. When participants report a diagnosis of cancer, medical records and pathology reports are reviewed by study physicians who are blinded to exposure data. Among those who provided baseline blood samples, colorectal cases were ascertained through March 31, 2008, and controls were matched on age (within one year for younger participants, up to five years for older participants) and smoking status (never, past, current). Cases were “pair” matched 1:1, 1:2 or 1:3 with a control participant(s). Due to DNA availability samples were genotyped in two batches on the same platform at the same genotyping center at different time points.

***Prostate, Lung, Colorectal, and Ovarian Cancer Screening Trial (PLCO)*.** PLCO enrolled 154,934 participants (men and women, aged between 55 and 74 years) at ten centers into a large, randomized, two-arm trial to determine the effectiveness of screening to reduce cancer mortality. Sequential blood samples were collected from participants assigned to the screening arm. Participation was 93% at the baseline blood draw. In the observational (control) arm, buccal cells were collected via mail using the “swish-and-spit” protocol and participation rate was 65%. Details of this study have been previously described (14,15) and are available online (http://dcp.cancer.gov/plco).
The Set 1 scan included a subset of 577 colon cancer cases self-reported as being non-Hispanic White with available DNA samples, questionnaire data, and appropriate consent for ancillary epidemiologic studies. Cases were excluded if they had a history of inflammatory bowel disease, polyps, polyposis syndrome or cancer (excluding basal or squamous cell skin cancer). Controls come from the Cancer Genetic Markers of Susceptibility (CGEMS) prostate cancer scan (all male) and the GWAS of Lung Cancer and Smoking [(18)](http://f1000.com/work/citation?ids=413036&pre=&suf=&sa=0) (enriched for smokers) along with an additional 92 non-Hispanic White female controls. For the Set 2 scan, cases were colorectal cancers from both arms of the trial, which were not already included in Set 1. Samples were excluded if participants did not sign appropriate consents, if DNA was unavailable, if baseline questionnaire data with follow-up were unavailable, if they had a history of colon cancer prior to the trial, if they were a rare cancer, and if they were already in colon GWAS, or if they were a control in the prostate or lung populations. Controls were frequency matched 1:1 to cases without replacement, and cases were not eligible to be controls. Matching criteria were age at enrollment (two year blocks), enrollment date (two year blocks), sex, race / ethnicity, trial arm, and study year of diagnosis (i.e. controls must be cancer free into the case's year of diagnosis).

***Postmenopausal Hormones Supplementary Study to the Colon Cancer Family Registry (PMH-CCFR)*** [(19)](http://f1000.com/work/citation?ids=2602579&pre=&suf=&sa=0). Eligible case patients included all female residents, ages 50 to 74 years, residing in the 13 counties in Washington State reporting to the Cancer Surveillance SEER program, who were newly diagnosed with invasive colorectal adenocarcinoma (ICD-O C18.0, C18.2-.9, C19.9, C20.0-.9) between October 1998 and February 2002. Eligibility for all individuals was limited to those who were English-speaking with available telephone numbers, in which they could be contacted. On average, cases were identified within four months of diagnosis. The overall response proportion of eligible cases identified was 73%. Community-based controls were randomly selected according to age distribution (in 5-year age intervals) of the eligible cases by using lists of licensed drivers from the Washington State Department of Licensing for individuals, ages 50 to 64 years, and rosters from the Health Care Financing Administration (now the Centers for Medicare and Medicaid) for individuals older than 64 years. The overall response proportion of eligible controls was 66%. In GECCO, samples with sufficient DNA extracted from blood were genotyped. Only participants that were not part of the CCFR Seattle site were included in the sample set.

***VITamins And Lifestyle (VITAL)*.** The VITamins And Lifestyle (VITAL) cohort comprises of 77,721 Washington State men and women aged 50 to 76 years, recruited from 2000 to 2002 to investigate the association of supplement use and lifestyle factors with cancer risk. Subjects were recruited by mail, from October 2000 to December 2002, using names purchased from a commercial mailing list. All subjects completed a 24 page questionnaire and buccal-cell specimens for DNA were self-collected by 70% of the participants. Subjects are followed for cancer by linkage to the western Washington SEER cancer registry and are censored when they move out of the area covered by the registry or at time of death. Details of this study have been previously described [(20)](http://f1000.com/work/citation?ids=2602639&pre=&suf=&sa=0). In GECCO, a nested case-control set was genotyped. Samples included, colorectal cancer cases with DNA, excluding subject with colorectal cancer before baseline, in situ cases, (large cell) neuroendocrine carcinoma, squamous cell carcinoma, carcinoid tumor, Goblet cell carcinoid, any type of lymphoma, including non-Hodgkin, Mantle cell, large B-cell, or follicular lymphoma. Controls were matched on age at enrollment (within one year), enrollment date (within one year), sex, and race / ethnicity. One control was randomly selected per case among all controls that matched on the four factors above and where the control follow-up time was greater than follow-up time of the case until diagnosis.

***Women’s Health Initiative (WHI).*** WHI is a long-term health study of 161,808 postmenopausal women aged 50 to 79 years at 40 clinical centers throughout the U.S. WHI comprises a Clinical Trial (CT) arm, an Observational Study (OS) arm, and several extension studies. The details of WHI have been previously described [(21, 22)](http://f1000.com/work/citation?ids=1209240,906653&pre=&pre=&suf=&suf=&sa=0,0) and are available online (https://cleo.whi.org/SitePages/Home.aspx). In GECCO, Set 1 cases were selected from the September 12, 2005 database and were comprised of centrally adjudicated colon cancer cases from the Observational Study (OS) who self-reported as White. Controls were first selected among controls previously genotyped as part of a Hip Fracture GWAS conducted within the WHI OS and matched to cases on age (within three years) enrollment date (within 365 days), hysterectomy status, and prevalent conditions at baseline. For 37 cases, there was not a control match in the Hip Fracture GWAS. For these participants, we identified a matched control in the WHI OS based on same criteria. In the Set 2 scan, cases were selected from the August 2009 database and were comprised of centrally adjudicated colon and colorectal cancer cases from the OS and CT who were not genotyped in Set 1. In addition, case and control participants were subject to the following exclusion criteria: a prior history of colorectal cancer at baseline, IRB approval not available for data submission into dbGaP, and not sufficient DNA available. Matching criteria included age (within years), race/ethnicity, WHI date (within three years), WHI Calcium and Vitamin D study date (within three years), and randomization arms (OS flag, hormone therapy assignments, dietary modification assignments, calcium/vitamin D assignments). In addition, they were matched on the four regions of randomization centers. Each case was matched with one control (1:1) that exactly met the matching criteria. Control selection was done in a time-forward manner, selecting one control for each case first from the risk set at the time of the case’s event. The matching algorithm was allowed to select the closest match based on a criterion to minimize an overall distance measure [(23)](http://f1000.com/work/citation?ids=2830662&pre=&suf=&sa=0). Each matching factor was given the same weight. Additional available controls that were genotyped as part of the Hip Fracture GWAS were included to improve power.

***Colon Cancer Family Registry (CCFR)*.** CCFR is a National Cancer Institute–supported consortium consisting of 6 centers dedicated to the establishment of a comprehensive collaborative infrastructure for interdisciplinary studies in the genetic
epidemiology of colorectal cancer [(24)](http://f1000.com/work/citation?ids=2301924&pre=&suf=&sa=0). The CCFR includes data from approximately 30,500 total subjects (10,500 probands and 20,000 unaffected and affected relatives and unrelated controls). Cases and controls, age 20 –74 years, were recruited at the 6 participating centers beginning in 1998. All participants completed a standardized questionnaire that asked about established and suspected risk factors for colorectal cancer, which included questions on medical history and medication use, reproductive history (for female participants), family history, physical activity, demographics, alcohol and tobacco use, and dietary factors. The set 1 scan, which has been described previously, [(25)](http://f1000.com/work/citation?ids=1636420&pre=&suf=&sa=0) included population-based cases and age-matched controls from the 3 population-based centers: Seattle, Toronto,and Australia. Cases were genetically enriched by oversampling those with a young age at onset or positive family history of colorectal cancer. Controls were matched to cases on age and sex. All cases and controls were self-reported as white, which was confirmed with genotype data. The set 2 scan included population-based cases and matched controls from all 6 colon CFR centers including the Mayo Clinic, Hawaii Cancer Registry, University of Southern California, Fred Hutchinson Cancer Research Center, Ontario Cancer Care, and University of Melbourne. As with set 1, cases were enriched genetically by oversampling those with a young age at onset or positive family history. Controls were same-generation family controls. In the discovery, 2151 participants were included from CCFR1 across all three sites and 811 participants were included from CCFR2 across all 6 sites.

*Replication Phase: Colorectal Transdisciplinary Study (CORECT)*

***Alpha-Tocopherol, Beta-Carotene Cancer Prevention (ATBC)*.** The ATBC Study was conducted in Finland as a joint project between the National Institute for Health and Welfare of Finland and the US National Cancer Institute (NCI). The overall design, rationale, objectives, and initial results of this intervention study have been published [(26, 27)](http://f1000.com/work/citation?ids=2830244,2836693&pre=&pre=&suf=&suf=&sa=0,0). Briefly, this was a randomized, double-blind, placebo-controlled, primary prevention trial to determine whether daily supplementation with alpha-tocopherol, beta-carotene, or both would reduce the incidence of lung or other cancers among male smokers. A total of 29,133 men between the ages of 50 and 69 years, who smoked at least five cigarettes per day, were recruited from southwestern Finland between 1985 and 1988, and randomly assigned to one of four groups based on a 2×2 factorial design. Men who had prior cancer or serious illness or who reported current use of vitamins E (>20mg/day), A (>20,000 IU/day), or beta-carotene (>6 mg/day) were ineligible. Participants received either alpha-tocopherol (50 mg/day) as dl-alpha-tocopheryl acetate, beta-carotene (20 mg/day) as all-trans-beta-carotene, both supplements, or placebo capsules for 5-8 years (median 6.1 years) until death or trial closure (April 30, 1993). Data Collection: At baseline, study subjects completed a general risk factor, smoking, and medical history questionnaire, along with a food frequency (use) questionnaire, which consisted of a modified diet history, including both portion size and frequency of consumption for 203 food items and 73 mixed dishes (1,2). This instrument was intended to measure usual consumption over the previous 12 months. Nutrient intake was estimated using food composition data available from the National Institute for Health and Welfare of Finland. Height, weight, blood pressure, heart rate, and visual acuity were measured. Cancer diagnoses are identified through linkage with the Finnish Cancer Registry

***Colocare Consortium.*** The ColoCare Study (clinical trials # NCT02328677) is a prospective cohort study of newly-diagnosed colorectal cancer (CRC) patients. The ColoCare Consortium is a multicenter initiative establishing an international cohort of colorectal cancer (CRC) patients for interdisciplinary studies of CRC prognosis and outcomes with sites at the Fred Hutchinson Cancer Research Center, Seattle (Washington, USA), H. Lee Moffitt Cancer Center and Research Institute, Tampa (Florida, USA), the University Hospital Heidelberg (Germany), and the Huntsman Cancer Institute (Utah, USA). The ColoCare Study investigates clinical outcomes, including disease-free and overall survival, predictors of cancer recurrence, health-related quality-of-life, and treatment toxicities. In addition, cross-sectional analyses of biomarkers and/or health behaviors are undertaken. Patients are recruited at baseline (time of first diagnosis) and followed for up to 5 years at regular timepoints (3 months (m), 6m, 12m, 24m, 36m, 48m, 60m). The cohort includes a comprehensive collection of specimens and data.
Patients included in the CORECT project were recruited at the following ColoCare sites: Fred Hutchinson Cancer Research Center (FHCRC) and the German Cancer Research Center (DKFZ, Heidelberg, HBG). CRC patients were recruited at the ColoCare Consortium sites when consulting with a colorectal surgeon or their staff as soon as possible after their diagnosis. Inclusion criteria for the ColoCare cohort are: (1) age 18-89 years, (2) newly-diagnosed CC (stages I-III), (3) English (FHCRC, Moffitt) or German (DKFZ) speaking, and (4) mentally/physically able to consent and participate. Pregnant women and prisoners are excluded. All activities including patient identification and recruitment, administration of health behavior questionnaires, specimen collection, medical record abstraction, biospecimen and data analysis are conducted according to IRB-approved protocols. Procedures and protocols for ColoCare FHCRC are currently approved under FHCRC IRB File 6407 and ColoCare Heidelberg (HBG) IRB approval has also been obtained (University of Heidelberg, 3/10/2010).

***Colon Cancer Family Registry (CCFR)*.** Cohort description is provided above under discovery. Replication included additional participants from the CCFR consortium that were not part of the discovery phase.

***American Cancer Society Cancer Prevention Study II (CPS-II).*** As described previously, CPS-II is a cohort study started by the American Cancer Society in 1982 to investigate the relationship between dietary, lifestyle and other etiologic factors and cancer mortality [(28)](http://f1000.com/work/citation?ids=2831054&pre=&suf=&sa=0). Approximately 1.2 million men and women were enrolled in the study from 50 states in the U.S. In 1992, a subset of these participants (N~184,000) were enrolled in the CPS-II Nutrition Cohort to examine the relationship between dietary and other exposures and cancer incidence. Blood samples were drawn from approximately 39,376 members of the Nutrition Cohort from 1998 to 2001, and buccal cells were collected from 69,467 additional members from 2001 to 2002. Cancer cases are identified by self-report through biennial follow-up questionnaires or through linkage with the National Death Index, followed by verification through medical records or linkage to state cancer registries. A total of 548 men and women diagnosed with colon or rectal cancer after providing a blood or buccal cell sample were genotyped for this study. Population-based control participants genotyped for this study included 538 men and women from the CPS-II Nutrition Cohort, individually matched to a case on sex, race/ethnicity, date of birth, date of sample collection, and DNA source (blood or buccal cell).

***Esther II/VERDI*** [(29, 30)](http://f1000.com/work/citation?ids=3812714,3812716&pre=&pre=&suf=&suf=&sa=0,0)***.*** In the ESTHER/VERDI study, patients diagnosed with various forms of cancer at ages 50-75, including patients with colorectal cancer (n=420), were recruited statewide in Saarland, Germany between 1996-1998 and 2001-2003. Controls, who were frequency matched by sex and age, were randomly drawn from women and men who were recruited for a statewide cohort study in Saarland, Germany when undergoing a health check-up with their general practitioners in 20002002 (n=437). Blood samples were drawn by the treating physicians who also provided medical data from their records. Risk factor information was collected by self-administered standardized questionnaires. The analytic dataset from the ESTHER/VERDI study included in the CORECT PHASE 2 GWAS consisted of 420 CRC cases and 437 controls.

***Kentucky.*** The Kentucky Case-Control study was initiated in July 2003 through the University of Kentucky Cancer Center. A web-based reporting system implemented by the Kentucky Cancer Registry in 2003 has facilitated rapid report of cases statewide, with approximately 76.8% of all cases reported to the registry within 6 months of diagnosis. Cases (>21 years) diagnosed with histologically confirmed colon cancer and entered into the registry within 6 months of their diagnoses are invited to join the study. Population-based unrelated controls are recruited through random digit dialing and are frequency matched to the cases by age (±5 years), gender, and race. Excluded from the study are those individuals who have been diagnosed with colon cancer because of known hereditary forms of colon cancer or polyposis such as familial adenomatous polyposis (FAP), hereditary non-polyposis colorectal cancer (HNPCC), Peutz-Jeghers, and Cowden disease. Currently there are more than 1,040 incident population-based cases of colorectal cancer and 1,750 population-based controls fully recruited, with comprehensive epidemiologic data, pathology data, and DNA from cases and controls

***Kiel (PopGen Biobank)*** [(31)](http://f1000.com/work/citation?ids=2818119&pre=&suf=&sa=0). All samples used in the present study were collected through the PopGen biobank (15). The CRC cases were members of a patient cohort from the Kiel area, described in detail elsewhere (16). Briefly, CRC patients who had been diagnosed or operated between 2002 and 2005 were identified through the cancer registry of Schleswig-Holstein or one of 25 surgical departments in Northern Germany, and were contacted by mail between August 2004 and December 2006. A total of 2,715 patients agreed to participate (response rate: 40%). All cases eventually included in the study had histologically proven CRC, a primary CRC diagnosis, and no previous cancer. The PopGen control samples [(32)](http://f1000.com/work/citation?ids=2833272&pre=&suf=&sa=0) were ascertained through the local population registry of Kiel and were contacted by mail between June 2005 and February 2006. Additional control individuals were recruited by way of convenience sampling from local blood donors. Blood donors were deemed eligible for inclusion if they were healthy non-first time donors and C18 years of age. In total, data on 1,317 control individuals were available for study, including 747 PopGen population controls and 570 blood donors. Venous EDTA blood samples were collected at baseline from both cases and controls, either at the PopGen facility or by local general practitioners. Genomic DNA (600–1,000 lg) was extracted by standard methods, using the Blood Gigakit (Invitek, Berlin, Germany), and stored under quality-controlled conditions at -20°C. At the time of enrollment, all study participants completed a baseline questionnaire or interview on their personal and family history of disease (including cancer) as well as on their height, weight and lifestyle factors (such as smoking and alcohol consumption). In addition, 2,067 of the CRC cases (76%) completed a follow-up questionnaire between August 2009 and April 2010 requesting information on their body weight 1 year before CRC was diagnosed. If the patients indicated presence of a first-degree relative with CRC, they were classified as having a ‘positive family history of CRC’. Patients without a family history of CRC were classified as ‘sporadic.’

***Korea: The Hwasun Cancer Epidemiology Study-Colon and Rectum Cancer (HCES-CRC)*** was a hospital-based case-control study conducted in South Korea. Cases were newly diagnosed CRC patients at Chonnam National University Hwasun Hospital, Jeollanam-do, South Korea from April 2004 to February 2013. Cancer-free controls were randomly selected from participants in the Korean Community Health Survey, an annual nationwide health interview survey, conducted from 2010 to 2012 in the Jindo and Bosung counties, Jeollanam-do, South Korea. The analytic dataset from the Korea study included in the CORECT Phase 2 GWAS consisted of 3,130 CRC cases and 2,854 controls.

***Melbourne Collaborative Cohort Study (MCCS).*** The MCCS includes both men and women volunteers, aged 40-69 and recruited from the Melbourne metropolitan area in the early 1990s. In order to recruit a sample with an increased range of dietary exposures, it was decided to deliberately enrich the cohort with migrants to Melbourne from Italy and Greece. The baseline questionnaire included questions on personal medical history and family history of common diseases. Other important environmental variables were also accounted for. Blood samples were collected from all subjects in 15ml lithium heparin vacutainers. Total plasma cholesterol and glucose were measured immediately using Kodak Ektachem DT60 desktop analysers. A total of 16962 men and 24286 women aged between 40 and 69 years were recruited into the cohort between 1990 and 1994 [(33)](http://f1000.com/work/citation?ids=2485675&pre=&suf=&sa=0).

***Multiethnic Cohort Study (MEC).*** Cohort description is provided above under discovery. Replication included additional participants from MEC that were not part of the discovery phase.

***Molecular Epidemiology of Colorectal Cancer (MECC) Study*** [(34)](http://f1000.com/work/citation?ids=2834663&pre=&suf=&sa=0). The Molecular Epidemiology of Colorectal Cancer Study (MECC) is a population-based case-control study of colorectal cancer (CRC). Incident, pathologically-confirmed CRC cases and controls were recruited from a specific region of northern Israel. Newly-diagnosed CRC cases beginning March 31, 1998, who agreed to participate were interviewed, gave a venous blood sample, and provided permission for tumor tissue retrieval. Written, informed consent was obtained according to Institutional Review Board-approved protocols at Carmel Medical Center in Haifa and the University of Southern California (HS-12-00324, HS-12-00672, and HS-08-00378). Germline DNA was extracted from whole blood for genotyping. The analytic dataset from the MECC study genotyped on the OncoArray and included in the CORECT Phase 2 European GWAS consisted of 3,591 cases of pathologically-confirmed adenocarcinoma and 2,848 controls. In addition, previously genotyped cases and controls were included in the Phase 1 GWAS: these consisted of 484 cases and 498 controls genotyped on the Illumina Omni 2.5 array, and 1,120 cases and 820 controls were genotyped on the Affymetrix Axiom CORECT Set array. Thus, the total number of cases and controls from the MECC study included in Phases 1 and 2 (after quality control for genotyping) was 5,195 cases and 4,166 controls.

***Memorial Sloan Kettering*** *(****MSKCC)*.** The Memorial Sloan Kettering (MSK) cohort consisted of 126 individuals of Ashkenazi Jewish descent with a diagnosis of colorectal cancer and no known germline mutations in colon cancer predisposition genes. Eligible patients were ascertained between 2001–2013 under three existing MSK IRB-approved protocols allowing for tumor/germline biospecimen collection and germline analysis for cancer susceptibility. Two of the protocols specifically focused on ascertainment of patients with either early-onset (age ≤ 50 at diagnosis) colorectal cancer or familial colorectal cancer with no identifiable germline mutations, while the third study included colorectal cancer patients irrespective of age or family cancer history. Patient data extracted from medical records included information on stage, tumor location, chemotherapy regimen received, history of medication use (HRT NSAIDs), endoscopy results, and metachronous or synchronous colorectal or other primary cancer diagnoses.

***Newfoundland Familial Colon Cancer Registry (NFCCR)*** [(35)](http://f1000.com/work/citation?ids=2836380&pre=&suf=&sa=0)**.** The NFCCR is a case-control study, which includes pathology confirmed CRC cases, less than 75 years of age, diagnosed between January 1, 1999 and December 31, 2003, identified from the Newfoundland Cancer Registry. The Newfoundland Cancer Registry registers all cases of invasive cancer diagnosed among residents of the province of Newfoundland and Labrador. Consenting patients received a family history questionnaire and were asked to provide a blood sample and to permit access to tumor tissue and medical records. If a patient was deceased, we sought the participation of a close relative for the purposes of obtaining the family history and for permission to access
tissue blocks and medical records. Use of proxies in this way removes the bias of excluding advanced-stage cancer patients who die before they can give consent. Controls were identified by random digit dialing from the residents of the province, and matched to the cases on sex and five year age group. Controls provided a blood sample and filled out a risk factor questionnaire.

***Nurses' Health Study II*** *(****NHSII)*.** The Nurses' Health Study II (NHSII) is an ongoing cohort of 116,430 female registered nurses in the US, aged 25-42 years at baseline in 1989. Demographic, lifestyle and health-related information were obtained from participants at baseline and updated every 2 years using self-administered questionnaires. The follow-up rate in each cycle has been over 90% to date. Study participants who had not previously reported a diagnosis of cancer and had responded to the 1995 NHSII study questionnaire were invited to provide blood samples between 1996 and 1999. Blood samples were collected from 29,611 NHSII participants, aged 32 to 54 years at the time of blood draw. 19 Similarly, between 2004 and 2006, active study participants who had not previously provided a blood sample were invited to provide buccal samples. Swish-and-spit sample of buccal cells were received from 29,859 participants. Cases and controls selected for genotyping were nested within the subcohort of participants who provided a blood or a buccal sample. Participants with a prior history of any cancer (except non-melanoma skin cancer), ulcerative colitis, or familial polyposis syndromes were excluded. Incident cases of colorectal adenocarcinoma were ascertained first by self-report and later confirmed by reviewing medical records and pathology reports within each follow up cycle. Deaths due to colorectal cancer were identified through family or next of kin or by querying the National Death Index. 20 Controls were randomly selected among participants in the subcohort provided they were free of colorectal cancer, and matched to a corresponding case by both age (within 1 year) and sample collection date (month/year of blood or buccal sampling). Overall, 133 cases and 132 matched controls were selected for OncoArray genotyping, and 109 cases and 102 controls with ≥80% estimated European ancestry based on STRUCTURE were included in the meta-analyses.

***Spain.*** The Spanish study combines data of three case-control studies. The first one, performed in University Hospital of Bellvitge, L'Hospitalet, Barcelona, recruited 304 incident, pathology confirmed, CRC cases and 293 age and sex frequency-matched hospital controls during the period 1996-1998. The control group consisted of patients without previous colorectal cancer who had been randomly selected among those admitted to the same hospital during the same period. To avoid selection bias, the criterion of inclusion in the control group was a new diagnosis. The second study, performed in the same hospital during the period 2007-2015, included a total of 324 cases and 376 population controls. The control group was recruited by inviting to participate subjects selected from the primary health care lists of the hospital’s referral area, frequency matched by age and sex. The third study was conducted in Hospital of Leon, Leon, during 2008-2013. A total of 325 incident CRC cases and 407 population controls were included. The control population was recruited by inviting to participate subjects selected from the primary health care lists, frequency matched by age and sex. Written informed consent was required from all participants. Each Hospital's ethics committees (Bellvitge and Leon) approved the protocols of the study.

***The Swedish Low-Risk Colorectal Cancer Study.*** During the years 2004-2009 more than 3300 consecutive patients operated on for colorectal cancer (CRC) in 14 hospitals in and around Stockholm and Uppsala were included in the Swedish Colorectal Cancer Low-risk study, and gave informed consent and blood for genetic studies. All cases were interviewed by the same person about their family history of colorectal cancer and other malignancies. Cancer in first- and second-degree relatives and cousins was recorded, and pedigrees for the families of the index-person (the patient) were constructed. All diagnoses in family members which could have been CRC were verified using medical records or death certificates. Other diagnoses were coded as stated by the index case. All haematological malignancies were coded as one entity as well as all gynaecological cancers because of difficulties in defining the exact diagnosis. Cases with no relative diagnosed with CRC were considered sporadic. Familial CRC was defined as cases with at least one relative with CRC in the family as defined above. All patients where relatives were at increased risk because of the family history were offered genetic counselling. Sex, age and tumor location of the index-patients were recorded based on the medical records. Tumors were assigned locations in caecum, ascending colon, hepatic flexure, transverse colon, splenic flexure, descending colon, the sigmoid or rectum. All tumors underwent evaluation directly after surgery by a local pathologist. The tumours were staged both according to the AJCC classification and the TNM-system. Some cases had two or more tumours and when tumours were located within the same segment they could be classified. As controls were used samples from 2,300 blood donors from the same region and 700 spouses to CRC patients, who did not have cancer and no family history of cancer. No information except gender were available for blood donors. For the spouse’s information on gender, age, height, weight were obtained. All patients gave written informed consents in accordance with Swedish legislation and the study was approved by the Regional research ethics committee, Dnr: 02-489.

***Sweden Wolk.*** The Swedish Mammography Cohort (SMC) and the Cohort of Swedish Men (COSM) are two large population-based prospective cohorts from central Sweden. The SMC was initiated between 1987 and 1990 when all women born in 1914-1948 and residing in Uppsala and Västmanland counties were invited; response rate 74% (n=66,651). The COSM started in late 1997, with the invitation of all men born in 1918-1952 and residing in Västmanland and Örebro county; response rate 49% (n=48,850). Questionnaire data on diet and other lifestyle factors was collected at the start of the studies, and has been updated repeatedly during follow-up. Further, biological samples (saliva, blood) have been collected together with signed informed consent and are available for DNA extraction. The cohorts are annually matched to the Swedish Cancer Register for ascertainment of incident cancer cases. For the CORECT study, follow-up through 2011 was available. The Regional Ethical Review Board at Karolinska Institutet in Stockholm approved genetic studies of CRC based on the cohorts. The analytic dataset from this study included in the CORECT Phase 2 GWAS consisted of 580 CRC cases and 859 controls.

***Studies of Epidemiology and Risk Factors in Cancer Heredity (SEARCH).*** The study started recruitment on March 1, 2001 and all CRC cases diagnosed between the ages of 18 and 69 since January 1, 1996 in the regions served by the Eastern Cancer Registration and Information Centre were eligible for inclusion. Recruitment continued until the end 2010. Sex and age (in 5-year age bands) frequency matched controls were identified from the registration lists of ten representative general practices across East Anglia (England). Controls were matched to cases participating in SEARCH breast, colorectal, prostate, ovarian and endometrial cancer studies. All participants completed an epidemiological questionnaire, provided a blood sample for DNA and provided written informed consent. Genotyping was carried out on all SEARCH colorectal cancer cases and controls that had provided a blood sample and returned a completed consent form. SEARCH is approved by the Cambridgeshire 4 Research Ethics Committee

***USC Norris Comprehensive Cancer Center Genetics Registry.*** The USC Norris Cancer Genetics Registry is a multicenter registry established to improve clinical care and to facilitate research to elucidate the genetic basis of hereditary cancers. Patients and families at risk of cancer are recruited from the University of Southern California Norris Comprehensive Cancer Center and LAC+USC Medical Center, where risk assessment, genetic counseling and clinical management are provided to individuals and families at risk for a variety of hereditary forms of cancer. Families at risk for hereditary forms of cancer are enrolled for continual follow-up to facilitate adherence to screening and management recommendations and to promote communication about risk to other relatives. Established in January, 2013, the USC Norris Cancer Genetics Registry provides the research structure for the development of a biorepository, clinical annotation, family expansion, longitudinal follow-up for research, germline and somatic DNA and RNA analysis, and analysis for the development of biostatistical and genetic models. For the OncoArray study, DNA samples and data from consented individuals from the USC Norris Cancer Genetics Registry with a pathologically confirmed diagnosis of adenocarcinoma of the colon or rectum were included for analysis, representing 234 colorectal cancer cases, and 221 were retained after QC. No controls were included from the registry for Oncoarray analyses.

*Replication Phase: UK Biobank*

***UK Biobank***

We constructed a CRC and advanced adenoma nested case-control dataset from the UK Biobank resource (application number 8614). CRC cases were defined as subjects with primary invasive CRC diagnosed, or who died from CRC according to ICD9 (1530-1534, 1536-1541) or ICD10 (C180, C182-C189, C19, C20) codes. Appendix cases, non-invasive (in situ) CRC cases, cases with histology of tumor as carcinoid, and related tumors and lymphomas (ICD-O-3 tumor histology codes 8240-8249, 9590-9729) were excluded. Advanced adenoma cases were defined as primary in situ CRC cases according to ICD9 (2303, 2304) or ICD10 (D010-D012) codes, or benign neoplasms according to ICD10 codes (D120, D122, D123, D124-D128, D374, D375) with ICD-O-3 tumor histology codes 8210, 8211, 8220, 8221, or 8261-8263. Incident and prevalent CRC or advanced adenoma cases were defined based on date of diagnosis and date of enrollment. Eligible control participants were required to be free of invasive colorectal cancer, non-invasive (*in situ*) CRC, appendix, anus, anal canal, and overlapping lesion of rectum, anus and anal canal cancer, or advanced adenoma. For incident cases, each case was matched with 4 controls that exactly matched the following matching criteria: age at enrollment, year at enrollment, race/ethnicity, and sex. Control selection was done in a time-forward manner, selecting one control for each case, first from the risk set at the time of the case’s event, and then multiple passes were made to match second, third and fourth controls. For prevalent cases, each case was matched with 4 controls that exactly matched the following matching criteria: year at enrollment, race/ethnicity, and sex. The risk set was then defined as controls who were at risk at the age when the cases were diagnosed. For matching of both incident and prevalent cases, the matching algorithm selected the closest match based on criteria to minimize an overall distance measure [50]. In total, 5,356 CRC (5,004) or advanced adenoma (352) cases and 21,407 matched controls were included in the replication analysis. All participants were genotyped using the Affymetrix UK Biobank Axiom Array.

*Replication Phase: Additional GWAS*

***Colorectal Cancer: Longitudinal Observational study on Nutritional and lifestyle factors that influence colorectal tumor recurrence, survival and quality of life (COLON).*** The COLON study is a multi-center prospective cohort study to assess the role of diet and other lifestyle factors in cancer recurrence and survival among incident colorectal cancer patients in the Netherlands. Patients with colorectal cancer from 11 hospitals were invited upon diagnosis. Patients with a history of colorectal cancer or (partial) bowel resection, chronic inflammatory bowel disease, hereditary colorectal cancer syndromes, or dementia were excluded from the study. At diagnosis and at several time points during follow-up, patients donated a blood sample and filled out questionnaires about diet and other lifestyle factors. Blood samples are stored in a biobank to facilitate future analyses. Information on vital status is retrieved by linkage with national registries. Information on clinical characteristics is gathered from linkage with the Netherlands Cancer Registry and with hospital databases. A total of 643 CRC cases were included in the replication analysis. Matching controls were selected from the Nutrition Questionnaires plus (NQplus) study. NQplus is a longitudinal observational study on diet and health in the general Dutch population. A total of 2,048 participants were recruited by inviting randomly selected inhabitants of the neighboring cities Wageningen, Ede, Renkum and Arnhem. In Veenendaal, another neighboring city, one individual of each household was invited to participate in the NQplus study. Baseline measurements consisted of a fasting venipuncture, dietary assessment, a physical examination, 24-h urine collection and general and lifestyle questionnaires. After excluding subjects with a history of colorectal cancer, chronic inflammatory bowel disease, or dementia, 692 controls were included in this study that were selected from the remaining participants and matched to the 643 CRC cases of the COLON study by age and gender. All participants were genotyped using the HumanOmniExpressExome-8v1-2 array.

***Darmkrebs: Chancen der Verhütung durch Screening (DACHS3)* (2,3)***.* This German study was initiated as a large population-based case-control study in 2003 in the Rhine-Neckar-Odenwald region (southwest region of Germany) to assess the potential of endoscopic screening for reduction of CRC risk and to investigate etiologic determinants of disease, particularly lifestyle/environmental factors and genetic factors. During an in-person interview, data were collected on demographics, medical history, family history of CRC, and various life-style factors, as were blood and mouthwash samples. Cases with a first diagnosis of invasive CRC (International Classification of Diseases 10 codes C18-C20) who were at least 30 years of age (no upper age limit), German speaking, a resident in the study region, and mentally and physically able to participate in a one-hour interview, were recruited by their treating physicians either in the hospital a few days after surgery, or by mail after discharge from the hospital. Cases were confirmed based on histologic reports and hospital discharge letters following diagnosis of CRC. All hospitals treating CRC cancer patients in the study region participated. Community-based controls were randomly selected from population registries, employing frequency matching with respect to age (5-year groups), sex, and county of residence. Controls with a history of CRC were excluded. Controls were contacted by mail and follow-up calls. The datasets analyzed in the replication study consist of 1,210 cases and 617 matched controls genotyped using the HumanOmniExpressExome-8v1-2 array (referred to as DACHS3).

***European Prospective Investigation into Cancer (EPIC).*** EPIC is an on-going multicenter prospective cohort study designed to investigate the associations between diet, lifestyle, genetic and environmental factors and various types of cancer. In summary, 521,448 participants (~70% women) mostly aged 35 years or above were recruited between 1992 and 2000. Participants were recruited from 23 study centers in ten European countries. The current study included participants from France, Germany, Greece, Italy, the Netherlands, Spain, Sweden, and United Kingdom (UK). Blood samples were collected at baseline according to standardized procedures, and stored at the International Agency for Research on Cancer (IARC; -196°C, liquid nitrogen) for all countries except Sweden (-80°C freezers). All study participants provided written informed consent. Ethical approval for the EPIC study was obtained from the review boards of IARC and local participating centers. Incident cancer cases were identified using population cancer registries in Italy, the Netherlands, Spain, and the United Kingdom. In Sweden (only the Umeå site was included), cases were identified by linkage with the essentially complete Cancer Registry of Northern Sweden and were verified by a gastrointestinal pathologist. In France, Germany and Greece, cancer cases were identified during follow-up by a combination of methods including: health insurance records, cancer and pathology registries, and by active follow-up directly through study participants or through next-of-kin. Controls were selected from the full cohort of individuals who were alive and free of cancer (except non-melanoma skin cancer) at the time of diagnoses of the cases, using incidence density sampling and matched by: age (±6 months at recruitment), sex, study center, follow-up time since blood collection, time of day at blood collection (±4 hours), fasting status, menopausal status, and phase of menstrual cycle at blood collection. In total, 2,095 incident colorectal cancer cases, and 2,306 matched controls were included in the replication analysis. All participants were genotyped using the HumanOmniExpressExome-8v1-2 array.

***Health Professionals Follow-up Study (HPFS3).*** HPFS is a parallel prospective study to the NHS. The HPFS cohort comprised 51,529 men aged 40-75 who, in 1986, responded to a mailed questionnaire. Participants provided information on health-related exposures, including current and past smoking history, age, weight, height, diet, physical activity, aspirin use, and family history of colorectal cancer. Colorectal cancer and other outcomes were reported by participants or next-of-kin and were followed up through review of the medical and pathology record by physicians. Overall, more than 97% of self-reported colorectal cancers were confirmed by medical record review. Information was abstracted on histology and primary location. Follow-up evaluation has been excellent, with 94% of the men responding to date. In 1993-1995, 18,825 men in the HPFS mailed blood samples by overnight courier, which were aliquoted into buffy coat and stored in liquid nitrogen. In 2001-2004, 13,956 men in the HPFS who had not provided a blood sample previously, mailed in a swish-and-spit sample of buccal cells. Incident cases were defined as those occurring after the subject provided a blood or buccal sample. Prevalent cases were defined as those occurring after enrollment in the study in 1986, but before the subject provided either a blood or buccal sample. Sample selection of the discovery stage case-control sets has been described in detail previously [1,2]. For the replication set (HPFS3), colorectal cancer cases were ascertained through January 1, 2010 and excluded cases included in the discovery stage [2]. Participants with histories of cancer (except nonmelanoma skin cancer), ulcerative colitis, or familial polyposis were excluded. CRC cases matched to randomly selected controls who provided a blood or buccal sample and were free of colorectal cancer at the same time the colorectal cancer was diagnosed in the cases. Matching criteria included year of birth (within 1 year) and month/year of blood or buccal cell sampling (within 1 year). If no control could be matched for a case using the initial stringent criteria, age criteria were relaxed to <5 years to find an eligible control. A total of 183 CRC cases and 197 controls were included in the replication analysis. All participants were genotyped using the HumanOmniExpressExome-8v1-2 array.

***Nurses’ Health Study (NHS3).*** The NHS cohort began in 1976 when 121,700 married female registered nurses age 30-55 years returned the initial questionnaire that ascertained a variety of important health-related exposures [52]. Since 1976, follow-up questionnaires have been mailed every 2 years. Colorectal cancer and other outcomes were reported by participants or next-of-kin and followed up through review of the medical and pathology record by physicians. Overall, more than 97% of self-reported colorectal cancers were confirmed by medical-record review. Information was abstracted on histology and primary location. The rate of follow-up evaluation has been high: as a proportion of the total possible follow-up time, follow-up evaluation has been more than 92%. In 1989-1990, 32,826 women in NHS I mailed blood samples by overnight courier, which were aliquoted into buffy coat and stored in liquid nitrogen. In 2001-2004, 29,684 women in NHS I who did not previously provide a blood sample mailed a swish-and-spit sample of buccal cells. Incident cases were defined as those occurring after the subject provided a blood or buccal sample. Prevalent cases were defined as those occurring after enrollment in the study in 1976 but before the subject provided either a blood or buccal sample. Sample selection of the discovery stage case-control sets has been described in detail previously [1,2]. For the replication set (NHS3), colorectal cancer cases were ascertained through June 1, 2012 and excluded cases included in the discovery GWAS [2]. Participants with histories of cancer (except nonmelanoma skin cancer), ulcerative colitis, or familial polyposis were excluded. CRC cases matched to randomly selected controls who provided a blood or buccal sample and were free of colorectal cancer at the same time the colorectal cancer was diagnosed in the cases. Matching criteria included year of birth (within 1 year) and month/year of blood or buccal cell sampling (within 1 year). If no control could be matched for a case using the initial stringent criteria, age criteria were relaxed to <5 years to find an eligible control. A total of 308 CRC cases and 303 controls were included in the replication analysis. All participants were genotyped using the HumanOmniExpressExome-8v1-2 array.

*Whole Genome Sequence Data*
Cases and controls were processed and sequenced together. Libraries were prepared with ThruPLEX DNA-seq kits (Rubicon Genomics) and paired-end sequencing performed using Illumina HiSeq 2500 sequencers. Reads were mapped to human reference genome (GRCh37 assembly) using the Burrows-Wheeler aligner BWA v0.6.2[45]. Fold genomic coverage averaged 5.3 and ranged from 3.8 to 8.6. We used the GotCloud population-based multi-sample variant calling pipeline[1] for post-processing of BAM files with initial alignments, and to detect and call SNPs. After removing duplicated reads and recalculation of base quality scores, sample QC prior to genotype calling included investigation of sample contamination. Variants were jointly called across all 2192 samples. To identify high-quality sites, the GotCloud pipeline performs a two-step filtering process. First, lower quality variants are identified by applying individual variant quality statistic hard filters. Next, variants failing multiple filters are used as negative examples to train a support vector machine (SVM) classifier. Finally, we performed a haplotype-aware genotype refinement step via Beagle[1] and ThunderVCF[1] on the SVM-filtered VCF files. After further sample QC, we excluded samples with estimated DNA contamination >3% (16), duplicated samples (5) or related individuals (1), sex discrepancies (0), and samples with low concordance with genome-wide SNP array data (11). We checked for ancestry outliers by performing principal components analysis (PCA) after merging in data for shared, linkage disequilibrium (LD)-pruned SNPs for 1092 individuals from the 1000 Genomes Project [1]. After QC, sequences were available for 1439 CRC cases and 720 controls of European ancestry. The average sequencing depth was 6.29. A total of 32,342,248 autosomal variants remained after QC. This internal sequencing panel was used for imputation into genotyped samples for both discovery and replication datasets as described in the main text.

**Transcriptome prediction in study cohort**

Variant weights used to impute gene expression were downloaded from the PredictDB databases on 11/1/2016 from (<http://predictdb.org/>). The following databases were used in this analysis 1) TW_Colon-Transverse_elasticNet0_0.5.db; 2) TW_Colon-Sigmoid_elasticNet0_0.5.db, and 3) DGN-WB_0.5.db.

**References**

[1. Küry S, et al. (2007) Combinations of cytochrome P450 gene polymorphisms enhancing the risk for sporadic colorectal cancer related to red meat consumption. *Cancer Epidemiol Biomarkers Prev* 16(7):1460–1467.](http://f1000.com/work/bibliography/2301922)

[2. Brenner H, Chang-Claude J, Seiler CM, Rickert A, Hoffmeister M (2011) Protection from colorectal cancer after colonoscopy: a population-based, case-control study. *Ann Intern Med* 154(1):22–30.](http://f1000.com/work/bibliography/2284876)

[3. Lilla C, et al. (2006) Effect of NAT1 and NAT2 genetic polymorphisms on colorectal cancer risk associated with exposure to tobacco smoke and meat consumption. *Cancer Epidemiol Biomarkers Prev* 15(1):99–107.](http://f1000.com/work/bibliography/2602353)

[4. Slattery ML, et al. (1997) Energy balance and colon cancer--beyond physical activity. *Cancer Res* 57(1):75–80.](http://f1000.com/work/bibliography/2301925)

[5. Le Marchand L, et al. (2001) Combined effects of well-done red meat, smoking, and rapid N-acetyltransferase 2 and CYP1A2 phenotypes in increasing colorectal cancer risk. *Cancer Epidemiol Biomarkers Prev* 10(12):1259–1266.](http://f1000.com/work/bibliography/2602355)

[6. Rimm EB, et al. (1990) Validity of self-reported waist and hip circumferences in men and women. *Epidemiology* 1(6):466–473.](http://f1000.com/work/bibliography/2602356)

[7. Kolonel LN, et al. (2000) A multiethnic cohort in Hawaii and Los Angeles: baseline characteristics. *Am J Epidemiol* 151(4):346–357.](http://f1000.com/work/bibliography/1986090)

[8. Belanger CF, Hennekens CH, Rosner B, Speizer FE (1978) The nurses’ health study. *Am J Nurs* 78(6):1039–1040.](http://f1000.com/work/bibliography/2602360)

[9. Cotterchio M, Manno M, Klar N, McLaughlin J, Gallinger S (2005) Colorectal screening is associated with reduced colorectal cancer risk: a case-control study within the population-based Ontario Familial Colorectal Cancer Registry. *Cancer Causes Control* 16(7):865–875.](http://f1000.com/work/bibliography/2601913)

[10. Cotterchio M, et al. (2000) Ontario familial colon cancer registry: methods and first-year response rates. *Chronic Dis Can* 21(2):81–86.](http://f1000.com/work/bibliography/2284858)

[11. Zanke BW, et al. (2007) Genome-wide association scan identifies a colorectal cancer susceptibility locus on chromosome 8q24. *Nat Genet* 39(8):989–994.](http://f1000.com/work/bibliography/1804202)

[12. Christen WG, Gaziano JM, Hennekens CH (2000) Design of Physicians’ Health Study II--a randomized trial of beta-carotene, vitamins E and C, and multivitamins, in prevention of cancer, cardiovascular disease, and eye disease, and review of results of completed trials. *Ann Epidemiol* 10(2):125–134.](http://f1000.com/work/bibliography/2301923)

[13. Hennekens CH, Eberlein K (1985) A randomized trial of aspirin and beta-carotene among U.S. physicians. *Prev Med* 14(2):165–168.](http://f1000.com/work/bibliography/2602363)

[14. Gohagan JK, Prorok PC, Hayes RB, Kramer BS, Prostate, Lung, Colorectal and Ovarian Cancer Screening Trial Project Team (2000) The Prostate, Lung, Colorectal and Ovarian (PLCO) Cancer Screening Trial of the National Cancer Institute: history, organization, and status. *Control Clin Trials* 21(6 Suppl):251S–272S.](http://f1000.com/work/bibliography/1513866)

[15. Prorok PC, et al. (2000) Design of the Prostate, Lung, Colorectal and Ovarian (PLCO) Cancer Screening Trial. *Control Clin Trials* 21(6 Suppl):273S–309S.](http://f1000.com/work/bibliography/1515357)

[16. Yeager M, et al. (2009) Identification of a new prostate cancer susceptibility locus on chromosome 8q24. *Nat Genet* 41(10):1055–1057.](http://f1000.com/work/bibliography/2602367)

[17. Cancer Genetic Markers of Susceptibility (CGEMS) Data Website Available at: http://cgems.cancer.gov/data/.](http://f1000.com/work/bibliography/2830275)

[18. Landi MT, et al. (2009) A genome-wide association study of lung cancer identifies a region of chromosome 5p15 associated with risk for adenocarcinoma. *Am J Hum Genet* 85(5):679–691.](http://f1000.com/work/bibliography/413036)

[19. Newcomb PA, et al. (2007) Estrogen plus progestin use, microsatellite instability, and the risk of colorectal cancer in women. *Cancer Res* 67(15):7534–7539.](http://f1000.com/work/bibliography/2602579)

[20. White E, et al. (2004) VITamins And Lifestyle cohort study: study design and characteristics of supplement users. *Am J Epidemiol* 159(1):83–93.](http://f1000.com/work/bibliography/2602639)

[21. Design of the Women’s Health Initiative clinical trial and observational study. The Women’s Health Initiative Study Group. (1998) *Control Clin Trials* 19(1):61–109.](http://f1000.com/work/bibliography/1209240)

[22. Hays J, et al. (2003) The Women’s Health Initiative recruitment methods and results. *Ann Epidemiol* 13(9 Suppl):S18-77.](http://f1000.com/work/bibliography/906653)

[23. Bergstralh EJ, Kosanke JL (1995) Computerized matching of cases to controls. 56.](http://f1000.com/work/bibliography/2830662)

[24. Newcomb PA, et al. (2007) Colon Cancer Family Registry: an international resource for studies of the genetic epidemiology of colon cancer. *Cancer Epidemiol Biomarkers Prev* 16(11):2331–2343.](http://f1000.com/work/bibliography/2301924)

[25. Figueiredo JC, et al. (2011) Genotype-environment interactions in microsatellite stable/microsatellite instability-low colorectal cancer: results from a genome-wide association study. *Cancer Epidemiol Biomarkers Prev* 20(5):758–766.](http://f1000.com/work/bibliography/1636420)

[26. The alpha-tocopherol, beta-carotene lung cancer prevention study: design, methods, participant characteristics, and compliance. The ATBC Cancer Prevention Study Group. (1994) *Ann Epidemiol* 4(1):1–10.](http://f1000.com/work/bibliography/2830244)

[27. The effect of vitamin E and beta carotene on the incidence of lung cancer and other cancers in male smokers. The Alpha-Tocopherol, Beta Carotene Cancer Prevention Study Group. (1994) *N Engl J Med* 330(15):1029–1035.](http://f1000.com/work/bibliography/2836693)

[28. Calle EE, et al. (2002) The American Cancer Society Cancer Prevention Study II Nutrition Cohort: rationale, study design, and baseline characteristics. *Cancer* 94(9):2490–2501.](http://f1000.com/work/bibliography/2831054)

[29. Jansen L, et al. (2011) Health-related quality of life during the 10 years after diagnosis of colorectal cancer: a population-based study. *J Clin Oncol* 29(24):3263–3269.](http://f1000.com/work/bibliography/3812714)

[30. Breitling LP, Raum E, Müller H, Rothenbacher D, Brenner H (2009) Synergism between smoking and alcohol consumption with respect to serum gamma-glutamyltransferase. *Hepatology* 49(3):802–808.](http://f1000.com/work/bibliography/3812716)

[31. Siegert S, et al. (2013) Genome-wide investigation of gene-environment interactions in colorectal cancer. *Hum Genet* 132(2):219–231.](http://f1000.com/work/bibliography/2818119)

[32. Krawczak M, et al. (2006) PopGen: population-based recruitment of patients and controls for the analysis of complex genotype-phenotype relationships. *Community Genet* 9(1):55–61.](http://f1000.com/work/bibliography/2833272)

[33. Giles GG, English DR (2002) The Melbourne Collaborative Cohort Study. *IARC Sci Publ* 156:69–70.](http://f1000.com/work/bibliography/2485675)

[34. Poynter JN, et al. (2005) Statins and the risk of colorectal cancer. *N Engl J Med* 352(21):2184–2192.](http://f1000.com/work/bibliography/2834663)

[35. Woods MO, et al. (2010) The genetic basis of colorectal cancer in a population-based incident cohort with a high rate of familial disease. *Gut* 59(10):1369–1377.](http://f1000.com/work/bibliography/2836380)

[36. Schumacher FR, et al. (2015) Genome-wide association study of colorectal cancer identifies six new susceptibility loci. *Nat Commun* 6:7138.](http://f1000.com/work/bibliography/1322474)

[37. Peters U, et al. (2013) Identification of Genetic Susceptibility Loci for Colorectal Tumors in a Genome-Wide Meta-analysis. *Gastroenterology* 144(4):799–807.e24.](http://f1000.com/work/bibliography/764284)

[38. Pritchard JK, Stephens M, Donnelly P (2000) Inference of population structure using multilocus genotype data. *Genetics* 155(2):945–959.](http://f1000.com/work/bibliography/704289)

[39. Howie B, Fuchsberger C, Stephens M, Marchini J, Abecasis GR (2012) Fast and accurate genotype imputation in genome-wide association studies through pre-phasing. *Nat Genet* 44(8):955–959.](http://f1000.com/work/bibliography/435556)

[40. Delaneau O, Howie B, Cox AJ, Zagury J-F, Marchini J (2013) Haplotype estimation using sequencing reads. *Am J Hum Genet* 93(4):687–696.](http://f1000.com/work/bibliography/148244)

[41. Das S, et al. (2016) Next-generation genotype imputation service and methods. *Nat Genet* 48(10):1284–1287.](http://f1000.com/work/bibliography/2094306)

[42. Gamazon ER, et al. (2015) A gene-based association method for mapping traits using reference transcriptome data. *Nat Genet* 47(9):1091–1098.](http://f1000.com/work/bibliography/756354)
