# Supplementary material for: Genetic variant predictors of gene expression provide new insight into risk of colorectal cancer
Source: Hum Genet. 2019 Feb 28;138(4):307–26. doi: 10.1007/s00439-019-01989-8 (PMC6483948; doi:10.1007/s00439-019-01989-8)
Supplement: Supplementary file 2 — Supplementary material 2 (PDF 258 KB) [file 439_2019_1989_MOESM2_ESM.pdf]

Table S2

Gene	Chr	R^2	Z	*P* value	Tissue
A4GALT	22	0.01	−2.41	0.02	GTEx_ColonTransverse
AAMP	2	0.01	3.14	1.72×10^{-3}	DGN_WholeBlood
AARD	8	0.01	2.31	0.02	GTEx_ColonTransverse
AATK	17	0.50	2.15	0.03	DGN_WholeBlood
ABCA2	9	0.21	2.01	0.04	DGN_WholeBlood
ABCB8	7	0.04	−2.10	0.04	DGN_WholeBlood
ABCC10	6	0.08	−2.13	0.03	GTEx_ColonTransverse
ABCD3	1	0.06	1.98	0.05	DGN_WholeBlood
ABCD4	14	0.02	2.27	0.02	DGN_WholeBlood
ABHD11	7	0.51	2.66	7.80×10^{-3}	DGN_WholeBlood
ABHD12B	14	0.05	−2.94	3.27×10^{-3}	GTEx_ColonTransverse
ABHD12B	14	0.08	−2.52	0.01	DGN_WholeBlood
ABTB2	11	0.20	−2.03	0.04	DGN_WholeBlood
AC011239.1	2	0.01	2.33	0.02	GTEx_ColonTransverse
AC011500.1	19	0.06	−1.96	0.05	GTEx_ColonTransverse
AC016757.3	2	0.01	2.50	0.01	GTEx_ColonTransverse
AC019294.1	15	0.01	−2.10	0.04	GTEx_ColonTransverse
AC078925.1	12	0.06	−1.98	0.05	GTEx_ColonTransverse
AC092675.3	2	0.03	−2.92	3.48×10^{-3}	GTEx_ColonTransverse
AC135178.1	17	0.05	−2.35	0.02	GTEx_ColonTransverse
ACAA1	3	0.02	2.17	0.03	DGN_WholeBlood

ACACB	12	0.29	−2.79	5.31×10^{-3}	DGN_WholeBlood
ACE	17	0.15	2.08	0.04	GTEx_ColonTransverse
ACOT7	1	0.01	3.56	3.74×10^{-4}	GTEx_ColonTransverse
ACSF3	16	0.24	3.08	2.06×10^{-3}	DGN_WholeBlood
ACSS3	12	0.12	−2.23	0.03	GTEx_ColonTransverse
ACTG2	2	0.05	2.31	0.02	DGN_WholeBlood
ACTR6	12	0.01	1.96	0.05	GTEx_ColonTransverse
ACTR8	3	0.05	1.98	0.05	GTEx_ColonTransverse
ADAM19	5	0.12	−2.02	0.04	DGN_WholeBlood
ADAMDEC1	8	0.16	2.06	0.04	DGN_WholeBlood
ADAMTS10	19	0.08	2.24	0.03	DGN_WholeBlood
ADAMTS19	5	0.01	2.39	0.02	GTEx_ColonTransverse
ADCY9	16	0.39	2.56	0.01	DGN_WholeBlood
ADI1	2	0.01	2.38	0.02	GTEx_ColonTransverse
ADIPOR2	12	0.04	−2.09	0.04	DGN_WholeBlood
ADPGK	15	0.09	2.38	0.02	DGN_WholeBlood
AGAP8	10	0.37	−2.11	0.03	DGN_WholeBlood
AGPAT3	21	0.01	2.42	0.02	GTEx_ColonTransverse
AGTPBP1	9	0.02	2.50	0.01	DGN_WholeBlood
AHI1	6	0.35	−2.09	0.04	GTEx_ColonTransverse
AHRR	5	0.08	−2.42	0.02	DGN_WholeBlood
AHSA2	2	0.28	2.20	0.03	GTEx_ColonTransverse
AIDA	1	0.02	−2.39	0.02	DGN_WholeBlood

AIG1	6	0.12	−2.13	0.03	DGN_WholeBlood
AIMP2	7	0.01	−2.49	0.01	GTEx_ColonTransverse
AK5	1	0.02	−2.31	0.02	GTEx_ColonTransverse
AK9	6	0.22	2.10	0.04	GTEx_ColonTransverse
AKR7A2	1	0.10	−2.00	0.05	DGN_WholeBlood
AL133216.1	10	0.04	2.10	0.04	GTEx_ColonTransverse
AL359091.2	9	0.06	−2.09	0.04	GTEx_ColonTransverse
ALDH1A1	9	0.10	2.50	0.01	DGN_WholeBlood
ALDH1L2	12	0.01	−2.01	0.04	DGN_WholeBlood
ALKBH5	17	0.10	−2.71	6.67×10^{-3}	DGN_WholeBlood
ALKBH8	11	0.04	−2.51	0.01	DGN_WholeBlood
ALMS1	2	0.21	−2.05	0.04	DGN_WholeBlood
ALOX12	17	0.06	−2.46	0.01	DGN_WholeBlood
ALOX15B	17	0.08	2.39	0.02	DGN_WholeBlood
AMIGO1	1	0.19	2.02	0.04	DGN_WholeBlood
AMY1B	1	0.01	−2.58	9.95×10^{-3}	GTEx_ColonTransverse
ANAPC5	12	0.02	2.35	0.02	GTEx_ColonTransverse
ANKLE1	19	0.36	2.87	4.05×10^{-3}	DGN_WholeBlood
ANKRD33	12	0.01	−2.39	0.02	GTEx_ColonTransverse
ANKRD36	2	0.06	−3.66	2.52×10^{-4}	DGN_WholeBlood
ANKRD54	22	0.16	2.26	0.02	DGN_WholeBlood
ANKS1A	6	0.04	−2.07	0.04	DGN_WholeBlood
ANXA1	9	0.10	2.76	5.74×10^{-3}	DGN_WholeBlood

AOC2	17	0.01	−2.13	0.03	DGN_WholeBlood
AP006621.5	11	0.43	−2.25	0.02	GTEx_ColonTransverse
AP1G1	16	0.01	−2.13	0.03	GTEx_ColonTransverse
AP2A2	11	0.02	2.46	0.01	DGN_WholeBlood
AP4B1	1	0.04	2.41	0.02	GTEx_ColonTransverse
APOF	12	0.03	−2.11	0.04	GTEx_ColonTransverse
APP	21	0.02	2.09	0.04	DGN_WholeBlood
ARF3	12	0.20	2.57	0.01	DGN_WholeBlood
ARFGAP3	22	0.01	2.02	0.04	DGN_WholeBlood
ARHGAP1	11	0.02	2.19	0.03	DGN_WholeBlood
ARHGAP32	11	0.10	2.21	0.03	DGN_WholeBlood
ARHGEF15	17	0.05	2.14	0.03	GTEx_ColonTransverse
ARHGEF19	1	0.63	−2.92	3.50×10^{-3}	DGN_WholeBlood
ARHGEF19	1	0.48	−2.73	6.32×10^{-3}	GTEx_ColonTransverse
ARID5A	2	0.17	−2.27	0.02	DGN_WholeBlood
ARL8B	3	0.05	2.05	0.04	DGN_WholeBlood
ARMC3	10	0.19	−2.29	0.02	DGN_WholeBlood
ARPC1B	7	0.02	−2.30	0.02	DGN_WholeBlood
ARPC2	2	0.03	3.53	4.16×10^{-4}	DGN_WholeBlood
ARPC5	1	0.13	−2.24	0.02	GTEx_ColonTransverse
ARRDC5	19	0.02	2.47	0.01	GTEx_ColonTransverse
ART5	11	0.06	−2.00	0.05	GTEx_ColonTransverse
ASAH2B	10	0.26	−2.46	0.01	GTEx_ColonTransverse

ASB8	12	0.21	3.13	1.74×10^{-3}	DGN_WholeBlood
ASNSD1	2	0.10	1.97	0.05	GTEx_ColonTransverse
ATF1	12	0.02	2.09	0.04	GTEx_ColonTransverse
ATG4B	2	0.04	2.06	0.04	GTEx_ColonTransverse
ATP1A2	1	0.06	−2.00	0.04	GTEx_ColonTransverse
ATP5G1	17	0.12	2.73	6.31×10^{-3}	DGN_WholeBlood
ATP5G2	12	0.20	−3.61	3.08×10^{-4}	GTEx_ColonTransverse
ATP5G2	12	0.04	−3.16	1.57×10^{-3}	DGN_WholeBlood
ATP5O	21	0.02	2.72	6.58×10^{-3}	DGN_WholeBlood
ATP6V0A2	12	0.18	−2.62	8.83×10^{-3}	DGN_WholeBlood
ATP6V0A2	12	0.12	−2.45	0.01	GTEx_ColonTransverse
ATP8B4	15	0.15	2.00	0.05	DGN_WholeBlood
ATP9B	18	0.33	−2.15	0.03	DGN_WholeBlood
ATXN3	14	0.11	−2.03	0.04	GTEx_ColonTransverse
ATXN7L1	7	0.03	−2.44	0.01	GTEx_ColonTransverse
ATXN7L2	1	0.02	−2.27	0.02	DGN_WholeBlood
AVIL	12	0.04	2.58	9.98×10^{-3}	DGN_WholeBlood
B3GNT8	19	0.01	−2.25	0.02	DGN_WholeBlood
B3GNTL1	17	0.01	3.10	1.94×10^{-3}	GTEx_ColonTransverse
B4GALT5	20	0.01	−2.67	7.49×10^{-3}	DGN_WholeBlood
B4GALT7	5	0.40	−2.64	8.22×10^{-3}	DGN_WholeBlood
BAG6	6	0.01	2.23	0.03	DGN_WholeBlood
BAHD1	15	0.03	2.33	0.02	DGN_WholeBlood

BAIAP2L2	22	0.06	2.75	5.95×10^{-3}	DGN_WholeBlood
BAZ1A	14	0.02	−2.22	0.03	DGN_WholeBlood
BAZ2A	12	0.03	−2.29	0.02	GTEx_ColonTransverse
BCAR3	1	0.02	2.13	0.03	DGN_WholeBlood
BCKDK	16	0.09	−2.54	0.01	DGN_WholeBlood
BDH1	3	0.05	−1.97	0.05	DGN_WholeBlood
BHLHA9	17	0.01	2.07	0.04	GTEx_ColonTransverse
BICD2	9	0.05	2.03	0.04	GTEx_ColonTransverse
BLMH	17	0.14	2.82	4.77×10^{-3}	DGN_WholeBlood
BMF	15	0.02	−2.02	0.04	GTEx_ColonTransverse
BMP10	2	0.04	2.20	0.03	GTEx_ColonTransverse
BMP5	6	0.01	2.00	0.05	GTEx_ColonTransverse
BMP7	20	0.02	2.82	4.84×10^{-3}	GTEx_ColonTransverse
BOLA1	1	0.03	−2.59	9.57×10^{-3}	GTEx_ColonTransverse
BPIFC	22	0.03	2.19	0.03	GTEx_ColonTransverse
BRP44L	6	0.23	−2.00	0.05	DGN_WholeBlood
BTN2A2	6	0.13	2.57	0.01	GTEx_ColonTransverse
BTN2A2	6	0.11	2.45	0.01	DGN_WholeBlood
BTNL8	5	0.38	2.12	0.03	GTEx_ColonTransverse
BTRC	10	0.04	1.97	0.05	DGN_WholeBlood
BZRAP1	17	0.02	−2.75	5.96×10^{-3}	DGN_WholeBlood
C10orf128	10	0.29	2.12	0.03	DGN_WholeBlood
C10orf88	10	0.13	2.41	0.02	DGN_WholeBlood

C11orf10	11	0.08	−3.50	4.69×10^{-4}	DGN_WholeBlood
C11orf45	11	0.02	2.04	0.04	DGN_WholeBlood
C11orf53	11	0.29	−4.33	1.50×10^{-5}	GTEx_ColonTransverse
C11orf9	11	0.25	−2.23	0.03	DGN_WholeBlood
C11orf92	11	0.26	−4.71	2.50×10^{-6}	GTEx_ColonTransverse
C11orf93	11	0.40	−4.83	1.39×10^{-6}	GTEx_ColonTransverse
C12orf79	12	0.01	−2.34	0.02	GTEx_ColonTransverse
C14orf182	14	0.15	2.44	0.01	DGN_WholeBlood
C14orf80	14	0.04	−3.20	1.39×10^{-3}	DGN_WholeBlood
C14orf93	14	0.02	3.15	1.61×10^{-3}	DGN_WholeBlood
C14orf93	14	0.01	−2.15	0.03	GTEx_ColonTransverse
C15orf40	15	0.12	−1.98	0.05	GTEx_ColonTransverse
C15orf57	15	0.06	−2.89	3.83×10^{-3}	DGN_WholeBlood
C15orf57	15	0.50	−2.08	0.04	GTEx_ColonTransverse
C16orf58	16	0.01	−2.35	0.02	GTEx_ColonTransverse
C16orf93	16	0.07	−2.45	0.01	DGN_WholeBlood
C17orf80	17	0.34	−2.55	0.01	DGN_WholeBlood
C17orf89	17	0.21	−2.06	0.04	DGN_WholeBlood
C1orf220	1	0.25	−2.04	0.04	DGN_WholeBlood
C1orf27	1	0.01	2.36	0.02	DGN_WholeBlood
C1orf38	1	0.22	−2.54	0.01	DGN_WholeBlood
C1QL3	10	0.68	2.21	0.03	DGN_WholeBlood
C1QL3	10	0.49	2.19	0.03	GTEx_ColonTransverse

C1QTNF6	22	0.50	−2.60	9.29×10^{-3}	DGN_WholeBlood
C2	6	0.09	1.99	0.05	DGN_WholeBlood
C21orf128	21	0.68	2.00	0.05	DGN_WholeBlood
C22orf43	22	0.69	−2.35	0.02	DGN_WholeBlood
C22orf43	22	0.50	−2.30	0.02	GTEx_ColonTransverse
C2CD4C	19	0.07	−2.16	0.03	GTEx_ColonTransverse
C2orf74	2	0.55	−2.16	0.03	DGN_WholeBlood
C2orf84	2	0.06	−1.99	0.05	DGN_WholeBlood
C2orf89	2	0.11	−2.61	9.18×10^{-3}	DGN_WholeBlood
C3orf18	3	0.02	−2.27	0.02	DGN_WholeBlood
C4BPB	1	0.23	−2.02	0.04	DGN_WholeBlood
C4orf47	4	0.01	2.48	0.01	GTEx_ColonTransverse
C5orf42	5	0.01	−2.07	0.04	GTEx_ColonTransverse
C6orf225	6	0.08	−2.65	8.08×10^{-3}	DGN_WholeBlood
C6orf57	6	0.08	−2.36	0.02	GTEx_ColonTransverse
C6orf62	6	0.22	−2.49	0.01	GTEx_ColonTransverse
C6orf62	6	0.17	−2.19	0.03	DGN_WholeBlood
C6orf89	6	0.02	−2.14	0.03	DGN_WholeBlood
C7orf25	7	0.59	−3.51	4.48×10^{-4}	DGN_WholeBlood
C7orf34	7	0.41	2.78	5.48×10^{-3}	DGN_WholeBlood
C7orf49	7	0.01	2.15	0.03	GTEx_ColonTransverse
C7orf61	7	0.02	2.38	0.02	DGN_WholeBlood
C9orf3	9	0.27	−2.10	0.04	DGN_WholeBlood

C9orf41	9	0.04	2.45	0.01	DGN_WholeBlood
C9orf69	9	0.04	2.51	0.01	DGN_WholeBlood
C9orf93	9	0.13	2.57	0.01	DGN_WholeBlood
CABP1	12	0.36	2.28	0.02	DGN_WholeBlood
CACNA2D4	12	0.04	−2.15	0.03	GTEx_ColonTransverse
CACTIN	19	0.01	−2.11	0.03	GTEx_ColonTransverse
CALCOCO1	12	0.02	2.13	0.03	GTEx_ColonTransverse
CALCOCO2	17	0.07	2.29	0.02	GTEx_ColonTransverse
CALCRL	2	0.04	−2.05	0.04	DGN_WholeBlood
CAMLG	5	0.18	2.10	0.04	DGN_WholeBlood
CAPN11	6	0.34	2.37	0.02	DGN_WholeBlood
CAPN2	1	0.05	−2.26	0.02	DGN_WholeBlood
CAPN8	1	0.01	3.37	7.65×10^{-4}	GTEx_ColonTransverse
CAPS	19	0.19	−1.99	0.05	DGN_WholeBlood
CARD6	5	0.01	2.84	4.55×10^{-3}	DGN_WholeBlood
CARS	11	0.15	2.77	5.57×10^{-3}	DGN_WholeBlood
CARS	11	0.05	2.74	6.11×10^{-3}	GTEx_ColonTransverse
CASP10	2	0.06	2.73	6.31×10^{-3}	DGN_WholeBlood
CASP8	2	0.20	2.40	0.02	GTEx_ColonTransverse
CASP8	2	0.33	2.12	0.03	DGN_WholeBlood
CBX3	7	0.05	2.19	0.03	DGN_WholeBlood
CC2D1B	1	0.10	−2.12	0.03	GTEx_ColonTransverse
CCDC137	17	0.04	2.41	0.02	DGN_WholeBlood

CCDC17	1	0.02	1.99	0.05	GTEx_ColonTransverse
CCDC64	12	0.06	2.45	0.01	DGN_WholeBlood
CCDC81	11	0.01	2.51	0.01	GTEx_ColonTransverse
CCDC85C	14	0.01	2.49	0.01	GTEx_ColonTransverse
CCDC97	19	0.01	−2.11	0.04	DGN_WholeBlood
CCHCR1	6	0.38	2.45	0.01	GTEx_ColonTransverse
CCM2	7	0.39	−2.03	0.04	DGN_WholeBlood
CCND2	12	0.01	−2.76	5.83×10^{-3}	DGN_WholeBlood
CCR8	3	0.04	−1.97	0.05	DGN_WholeBlood
CCT7	2	0.01	2.10	0.04	DGN_WholeBlood
CCZ1	7	0.29	2.13	0.03	GTEx_ColonTransverse
CD200	3	0.01	2.21	0.03	GTEx_ColonTransverse
CD36	7	0.01	−2.18	0.03	GTEx_ColonTransverse
CD3G	11	0.03	−2.05	0.04	DGN_WholeBlood
CD59	11	0.21	−2.59	9.61×10^{-3}	DGN_WholeBlood
CDC37L1	9	0.03	−2.17	0.03	GTEx_ColonTransverse
CDC42	1	0.09	−2.40	0.02	DGN_WholeBlood
CDCA2	8	0.07	2.01	0.04	GTEx_ColonTransverse
CDCA7	2	0.14	2.30	0.02	GTEx_ColonTransverse
CDH15	16	0.06	2.51	0.01	GTEx_ColonTransverse
CDHR1	10	0.16	2.59	9.54×10^{-3}	DGN_WholeBlood
CDHR2	5	0.02	2.51	0.01	DGN_WholeBlood
CDIP1	16	0.13	−2.10	0.04	GTEx_ColonTransverse

CDK11B	1	0.02	−2.14	0.03	GTEx_ColonTransverse
CDK18	1	0.04	2.09	0.04	DGN_WholeBlood
CDK2AP1	12	0.26	1.97	0.05	GTEx_ColonTransverse
CEACAM18	19	0.24	2.16	0.03	GTEx_ColonTransverse
CECR6	22	0.48	−2.00	0.05	DGN_WholeBlood
CEL	9	0.02	2.22	0.03	GTEx_ColonTransverse
CELSR1	22	0.04	2.15	0.03	GTEx_ColonTransverse
CEND1	11	0.09	−2.49	0.01	DGN_WholeBlood
CENPC	4	0.04	−2.11	0.03	GTEx_ColonTransverse
CENPF	1	0.02	−2.12	0.03	GTEx_ColonTransverse
CEP57L1	6	0.04	2.05	0.04	GTEx_ColonTransverse
CERS5	12	0.06	−2.98	2.91×10^{-3}	GTEx_ColonTransverse
CFHR4	1	0.02	2.14	0.03	GTEx_ColonTransverse
CGNL1	15	0.04	−2.29	0.02	GTEx_ColonTransverse
CHCHD6	3	0.02	2.89	3.87×10^{-3}	DGN_WholeBlood
CHML	1	0.02	1.99	0.05	DGN_WholeBlood
CHMP2A	19	0.01	2.09	0.04	DGN_WholeBlood
CHRNB1	17	0.08	2.02	0.04	DGN_WholeBlood
CHRNE	17	0.33	−2.01	0.04	DGN_WholeBlood
CHST10	2	0.01	2.34	0.02	GTEx_ColonTransverse
CHST14	15	0.02	2.69	7.16×10^{-3}	DGN_WholeBlood
CIITA	16	0.09	−2.58	9.85×10^{-3}	DGN_WholeBlood
CKAP2L	2	0.01	−2.52	0.01	DGN_WholeBlood

CKAP5	11	0.01	−2.56	0.01	GTEx_ColonTransverse
CKS2	9	0.19	−2.56	0.01	DGN_WholeBlood
CLCN1	7	0.66	2.39	0.02	DGN_WholeBlood
CLDN9	16	0.01	1.96	0.05	DGN_WholeBlood
CLIC6	21	0.13	2.35	0.02	GTEx_ColonTransverse
CLIC6	21	0.07	−2.22	0.03	DGN_WholeBlood
CLLU1OS	12	0.01	−3.17	1.50×10^{-3}	GTEx_ColonTransverse
CMYA5	5	0.03	2.13	0.03	DGN_WholeBlood
CNGA3	2	0.04	−3.07	2.12×10^{-3}	GTEx_ColonTransverse
CNNM3	2	0.03	−2.27	0.02	GTEx_ColonTransverse
CNPY4	7	0.04	−2.69	7.22×10^{-3}	GTEx_ColonTransverse
CNTROB	17	0.22	2.35	0.02	DGN_WholeBlood
COG1	17	0.05	2.47	0.01	DGN_WholeBlood
COL23A1	5	0.27	−2.17	0.03	DGN_WholeBlood
COL5A3	19	0.60	−2.12	0.03	DGN_WholeBlood
COPS3	17	0.31	−2.97	3.02×10^{-3}	DGN_WholeBlood
COPS6	7	0.05	−3.37	7.60×10^{-4}	DGN_WholeBlood
COQ10A	12	0.00	−2.48	0.01	DGN_WholeBlood
CORO6	17	0.08	−2.37	0.02	DGN_WholeBlood
CORO7	16	0.02	−2.67	7.50×10^{-3}	DGN_WholeBlood
COX11	17	0.19	−2.56	0.01	DGN_WholeBlood
COX14	12	0.01	3.47	5.19×10^{-4}	GTEx_ColonTransverse
COX15	10	0.11	−2.83	4.59×10^{-3}	DGN_WholeBlood

COX20	1	0.03	1.99	0.05	GTEx_ColonTransverse
CPSF2	14	0.11	2.28	0.02	GTEx_ColonTransverse
CRB3	19	0.13	2.20	0.03	DGN_WholeBlood
CREG1	1	0.06	−2.69	7.11×10^{-3}	GTEx_ColonTransverse
CRYL1	13	0.16	1.97	0.05	DGN_WholeBlood
CRYM	16	0.12	−2.53	0.01	DGN_WholeBlood
CSF1	1	0.02	2.59	9.60×10^{-3}	DGN_WholeBlood
CSF1R	5	0.04	−2.47	0.01	DGN_WholeBlood
CSNK1G1	15	0.08	−2.43	0.02	DGN_WholeBlood
CSTF3	11	0.02	−2.85	4.41×10^{-3}	DGN_WholeBlood
CTNNAL1	9	0.00	2.57	0.01	GTEx_ColonTransverse
CTNNBIP1	1	0.06	2.00	0.05	DGN_WholeBlood
CUL2	10	0.01	−2.10	0.04	DGN_WholeBlood
CUL4A	13	0.02	−2.29	0.02	DGN_WholeBlood
CUL9	6	0.06	2.16	0.03	DGN_WholeBlood
CUTC	10	0.01	−2.79	5.35×10^{-3}	GTEx_ColonTransverse
CWF19L2	11	0.03	−2.70	6.90×10^{-3}	DGN_WholeBlood
CXCL2	4	0.01	−2.23	0.03	GTEx_ColonTransverse
CXCR1	2	0.17	3.95	7.75×10^{-5}	DGN_WholeBlood
CXCR2	2	0.15	3.89	9.94×10^{-5}	DGN_WholeBlood
CXXC1	18	0.01	2.00	0.05	GTEx_ColonTransverse
CXXC5	5	0.01	−3.08	2.06×10^{-3}	DGN_WholeBlood
CYB5R4	6	0.01	−1.97	0.05	DGN_WholeBlood

CYP21A2	6	0.23	2.33	0.02	DGN_WholeBlood
CYP24A1	20	0.14	−1.99	0.05	DGN_WholeBlood
CYP27B1	12	0.01	2.94	3.32×10^{-3}	DGN_WholeBlood
CYP4V2	4	0.44	−2.53	0.01	DGN_WholeBlood
DAD1	14	0.19	2.28	0.02	DGN_WholeBlood
DAGLA	11	0.04	2.16	0.03	DGN_WholeBlood
DAPK1	9	0.50	−2.25	0.02	DGN_WholeBlood
DBNDD2	20	0.11	−2.33	0.02	GTEx_ColonTransverse
DCBLD1	6	0.05	2.61	9.10×10^{-3}	GTEx_ColonTransverse
DCBLD1	6	0.35	−2.56	0.01	DGN_WholeBlood
DDAH2	6	0.02	2.35	0.02	GTEx_ColonTransverse
DDRGK1	20	0.04	2.09	0.04	DGN_WholeBlood
DDX18	2	0.13	−2.17	0.03	DGN_WholeBlood
DDX20	1	0.01	2.32	0.02	DGN_WholeBlood
DDX47	12	0.02	−2.00	0.05	DGN_WholeBlood
DENND2D	1	0.08	2.12	0.03	DGN_WholeBlood
DEPDC7	11	0.23	−2.47	0.01	DGN_WholeBlood
DEPDC7	11	0.02	−2.16	0.03	GTEx_ColonTransverse
DEXI	16	0.09	2.86	4.29×10^{-3}	DGN_WholeBlood
DGAT2	11	0.04	2.30	0.02	DGN_WholeBlood
DHDDS	1	0.12	−2.00	0.05	DGN_WholeBlood
DHTKD1	10	0.12	2.04	0.04	DGN_WholeBlood
DICER1	14	0.01	−2.13	0.03	DGN_WholeBlood

DIP2B	12	0.55	3.64	2.73×10^{-4}	DGN_WholeBlood
DLAT	11	0.01	−2.27	0.02	GTEx_ColonTransverse
DLST	14	0.01	−2.78	5.35×10^{-3}	DGN_WholeBlood
DMC1	22	0.01	−2.06	0.04	GTEx_ColonTransverse
DNAJA3	16	0.02	2.44	0.01	DGN_WholeBlood
DNAJB7	22	0.61	2.02	0.04	DGN_WholeBlood
DNAJC1	10	0.01	−3.36	7.89×10^{-4}	DGN_WholeBlood
DNAJC17	15	0.01	−3.19	1.41×10^{-3}	DGN_WholeBlood
DNASE1	16	0.12	2.32	0.02	DGN_WholeBlood
DNTTIP1	20	0.28	2.53	0.01	DGN_WholeBlood
DPEP1	16	0.03	−2.17	0.03	GTEx_ColonTransverse
DPP7	9	0.11	−2.07	0.04	DGN_WholeBlood
DSC2	18	0.05	2.28	0.02	GTEx_ColonTransverse
DSP	6	0.67	−2.63	8.64×10^{-3}	DGN_WholeBlood
DST	6	0.01	1.98	0.05	DGN_WholeBlood
DSTYK	1	0.05	−3.30	9.54×10^{-4}	DGN_WholeBlood
DTNB	2	0.08	2.09	0.04	DGN_WholeBlood
DTWD1	15	0.03	−3.29	9.90×10^{-4}	DGN_WholeBlood
DTX3	12	0.02	−2.07	0.04	DGN_WholeBlood
DTYMK	2	0.04	2.49	0.01	GTEx_ColonTransverse
DUSP16	12	0.22	2.21	0.03	DGN_WholeBlood
DUSP23	1	0.11	−1.96	0.05	DGN_WholeBlood
EBAG9	8	0.02	−2.16	0.03	DGN_WholeBlood

ECHDC2	1	0.33	−2.10	0.04	DGN_WholeBlood
EDEM2	20	0.16	2.18	0.03	DGN_WholeBlood
EFCAB5	17	0.11	2.45	0.01	DGN_WholeBlood
EFCAB5	17	0.14	2.32	0.02	GTEx_ColonTransverse
EFR3B	2	0.09	2.52	0.01	DGN_WholeBlood
EFTUD2	17	0.01	−2.00	0.05	GTEx_ColonTransverse
EIF2B1	12	0.10	−1.99	0.05	DGN_WholeBlood
ELOVL5	6	0.02	−2.33	0.02	DGN_WholeBlood
EMILIN2	18	0.07	1.98	0.05	DGN_WholeBlood
ENTPD7	10	0.01	−2.14	0.03	DGN_WholeBlood
EPS8L2	11	0.36	−2.06	0.04	DGN_WholeBlood
ERGIC3	20	0.05	−2.01	0.04	DGN_WholeBlood
ESD	13	0.01	−2.33	0.02	GTEx_ColonTransverse
ESRP2	16	0.05	2.65	8.15×10^{-3}	DGN_WholeBlood
ETV2	19	0.04	−2.79	5.23×10^{-3}	DGN_WholeBlood
ETV7	6	0.52	−2.48	0.01	DGN_WholeBlood
EVC2	4	0.10	2.07	0.04	DGN_WholeBlood
EVI2A	17	0.06	−2.07	0.04	DGN_WholeBlood
EVI5	1	0.15	2.54	0.01	DGN_WholeBlood
EVI5	1	0.08	2.46	0.01	GTEx_ColonTransverse
F13A1	6	0.15	−2.18	0.03	DGN_WholeBlood
FABP2	4	0.15	2.09	0.04	GTEx_ColonTransverse
FADS1	11	0.24	−3.37	7.42×10^{-4}	DGN_WholeBlood

FADS2	11	0.88	−3.11	1.90×10^{-3}	DGN_WholeBlood
FADS2	11	0.20	−2.91	3.64×10^{-3}	GTEx_ColonTransverse
FADS3	11	0.09	1.97	0.05	DGN_WholeBlood
FAM104A	17	0.03	−2.24	0.02	DGN_WholeBlood
FAM116B	22	0.12	2.23	0.03	DGN_WholeBlood
FAM131B	7	0.16	2.05	0.04	DGN_WholeBlood
FAM149B1	10	0.08	−2.07	0.04	DGN_WholeBlood
FAM153A	5	0.08	−2.13	0.03	DGN_WholeBlood
FAM154B	15	0.41	2.36	0.02	GTEx_ColonTransverse
FAM154B	15	0.60	2.17	0.03	DGN_WholeBlood
FAM170B	10	0.24	3.24	1.18×10^{-3}	DGN_WholeBlood
FAM177A1	14	0.40	−2.11	0.04	DGN_WholeBlood
FAM177B	1	0.30	−2.43	0.02	DGN_WholeBlood
FAM178B	2	0.04	2.37	0.02	GTEx_ColonTransverse
FAM178B	2	0.02	−2.20	0.03	DGN_WholeBlood
FAM184B	4	0.01	2.27	0.02	DGN_WholeBlood
FAM184B	4	0.06	−2.09	0.04	GTEx_ColonTransverse
FAM190B	10	0.47	−2.27	0.02	DGN_WholeBlood
FAM24B	10	0.01	1.99	0.05	DGN_WholeBlood
FAM50B	6	0.11	−2.05	0.04	DGN_WholeBlood
FAM57A	17	0.18	3.24	1.19×10^{-3}	GTEx_ColonTransverse
FAM57A	17	0.07	2.57	0.01	DGN_WholeBlood
FAM64A	17	0.04	2.33	0.02	DGN_WholeBlood

FAM65C	20	0.13	−2.36	0.02	DGN_WholeBlood
FAM69A	1	0.08	−2.29	0.02	DGN_WholeBlood
FAM81B	5	0.07	2.05	0.04	GTEx_ColonTransverse
FAM84B	8	0.05	−2.35	0.02	GTEx_ColonTransverse
FANCI	15	0.00	2.35	0.02	DGN_WholeBlood
FARP1	13	0.32	2.29	0.02	DGN_WholeBlood
FBLN2	3	0.02	2.05	0.04	GTEx_ColonTransverse
FBN1	15	0.21	−2.16	0.03	DGN_WholeBlood
FBXL13	7	0.08	−2.23	0.03	DGN_WholeBlood
FBXL20	17	0.10	−2.77	5.62×10^{-3}	GTEx_ColonTransverse
FCGR1A	1	0.07	2.33	0.02	DGN_WholeBlood
FEM1B	15	0.01	−2.64	8.41×10^{-3}	GTEx_ColonTransverse
FGD3	9	0.05	2.39	0.02	GTEx_ColonTransverse
FHOD1	16	0.13	−2.02	0.04	DGN_WholeBlood
FIBIN	11	0.01	−2.15	0.03	GTEx_ColonTransverse
FIZ1	19	0.04	−2.17	0.03	DGN_WholeBlood
FKBP1A	20	0.05	2.10	0.04	DGN_WholeBlood
FLCN	17	0.07	−3.44	5.89×10^{-4}	DGN_WholeBlood
FLCN	17	0.56	−2.98	2.89×10^{-3}	GTEx_ColonTransverse
FN3KRP	17	0.50	2.07	0.04	GTEx_ColonTransverse
FNIP2	4	0.08	−2.99	2.77×10^{-3}	DGN_WholeBlood
FOXK2	17	0.05	−2.06	0.04	DGN_WholeBlood
FRAS1	4	0.04	−2.00	0.04	GTEx_ColonTransverse

FRG1B	20	0.02	1.97	0.05	GTEx_ColonTransverse
FSCN1	7	0.01	−2.00	0.05	DGN_WholeBlood
FUT7	9	0.36	−2.31	0.02	DGN_WholeBlood
FXR1	3	0.01	3.43	5.99×10^{-4}	DGN_WholeBlood
FYCO1	3	0.02	−2.39	0.02	DGN_WholeBlood
FZD7	2	0.02	−2.28	0.02	DGN_WholeBlood
G3BP1	5	0.02	−2.04	0.04	DGN_WholeBlood
GAL3ST4	7	0.07	−2.13	0.03	DGN_WholeBlood
GALNT6	12	0.30	2.52	0.01	DGN_WholeBlood
GATS	7	0.31	−2.97	2.96×10^{-3}	DGN_WholeBlood
GATSL3	22	0.25	2.06	0.04	DGN_WholeBlood
GCOM1	15	0.01	−2.04	0.04	GTEx_ColonTransverse
GDF10	10	0.03	−2.02	0.04	DGN_WholeBlood
GDF5	20	0.01	−2.17	0.03	GTEx_ColonTransverse
GEMIN4	17	0.06	2.14	0.03	GTEx_ColonTransverse
GEMIN4	17	0.08	2.13	0.03	DGN_WholeBlood
GHRH	20	0.03	2.51	0.01	GTEx_ColonTransverse
GIT1	17	0.40	−2.57	0.01	DGN_WholeBlood
GJB4	1	0.03	2.11	0.04	GTEx_ColonTransverse
GLP2R	17	0.07	−1.98	0.05	GTEx_ColonTransverse
GLRX	5	0.07	2.00	0.05	DGN_WholeBlood
GLTSCR1	19	0.03	−2.32	0.02	DGN_WholeBlood
GLYATL1	11	0.01	2.15	0.03	GTEx_ColonTransverse

GM2A	5	0.79	−2.06	0.04	DGN_WholeBlood
GMCL1	2	0.07	−2.33	0.02	DGN_WholeBlood
GMEB2	20	0.08	2.25	0.02	DGN_WholeBlood
GNA12	7	0.10	−2.52	0.01	GTEx_ColonTransverse
GNA15	19	0.02	−2.16	0.03	GTEx_ColonTransverse
GNG8	19	0.42	−2.05	0.04	DGN_WholeBlood
GNS	12	0.03	2.57	0.01	DGN_WholeBlood
GOLGA6L9	15	0.04	−2.85	4.31×10^{-3}	GTEx_ColonTransverse
GOLGA8N	15	0.11	2.02	0.04	GTEx_ColonTransverse
GPAM	10	0.00	−1.99	0.05	DGN_WholeBlood
GPATCH3	1	0.02	−1.96	0.05	DGN_WholeBlood
GPBAR1	2	0.01	2.95	3.17×10^{-3}	GTEx_ColonTransverse
GPBAR1	2	0.04	−2.42	0.02	DGN_WholeBlood
GPD1L	3	0.34	2.50	0.01	DGN_WholeBlood
GPLD1	6	0.01	−2.05	0.04	GTEx_ColonTransverse
GPNMB	7	0.30	−2.68	7.37×10^{-3}	DGN_WholeBlood
GPR18	13	0.02	−2.12	0.03	DGN_WholeBlood
GRID2IP	7	0.08	−2.02	0.04	DGN_WholeBlood
GTF2H2	5	0.13	−2.35	0.02	DGN_WholeBlood
GTF2H4	6	0.10	−2.38	0.02	DGN_WholeBlood
GTPBP10	7	0.16	2.23	0.03	DGN_WholeBlood
GUCY2D	17	0.07	2.70	6.93×10^{-3}	DGN_WholeBlood
GYPC	2	0.07	1.99	0.05	DGN_WholeBlood

H1F0	22	0.01	2.49	0.01	GTEx_ColonTransverse
H2AFX	11	0.02	2.03	0.04	GTEx_ColonTransverse
H6PD	1	0.10	2.56	0.01	GTEx_ColonTransverse
H6PD	1	0.38	2.40	0.02	DGN_WholeBlood
HAUS4	14	0.20	3.12	1.78×10^{-3}	GTEx_ColonTransverse
HDDC2	6	0.44	2.41	0.02	DGN_WholeBlood
HDGF	1	0.01	2.07	0.04	DGN_WholeBlood
HDLBP	2	0.00	−1.99	0.05	GTEx_ColonTransverse
HEATR3	16	0.30	2.20	0.03	GTEx_ColonTransverse
HECTD4	12	0.01	−4.50	6.69×10^{-6}	GTEx_ColonTransverse
HELB	12	0.02	2.15	0.03	GTEx_ColonTransverse
HERC4	10	0.01	−2.22	0.03	DGN_WholeBlood
HES4	1	0.04	−2.44	0.01	GTEx_ColonTransverse
HEXB	5	0.01	2.63	8.42×10^{-3}	GTEx_ColonTransverse
HIF1AN	10	0.22	2.14	0.03	DGN_WholeBlood
HIP1	7	0.03	2.13	0.03	GTEx_ColonTransverse
HIST1H2BK	6	0.13	−2.12	0.03	DGN_WholeBlood
HIST1H2BO	6	0.01	−2.10	0.04	DGN_WholeBlood
HIVEP1	6	0.01	−2.23	0.03	DGN_WholeBlood
HK1	10	0.01	2.84	4.56×10^{-3}	GTEx_ColonTransverse
HLA−DMA	6	0.05	2.25	0.02	GTEx_ColonTransverse
HLA−DRB1	6	0.63	−2.65	7.96×10^{-3}	GTEx_ColonTransverse
HLA−DRB5	6	0.66	−2.94	3.24×10^{-3}	GTEx_ColonTransverse

HLA−F	6	0.27	2.43	0.02	GTEx_ColonTransverse
HLA.A	6	0.26	−1.99	0.05	DGN_WholeBlood
HLA.DRB5	6	0.85	−2.51	0.01	DGN_WholeBlood
HLA.F	6	0.48	2.08	0.04	DGN_WholeBlood
HM13	20	0.28	2.94	3.31×10^{-3}	DGN_WholeBlood
HNMT	2	0.07	2.37	0.02	DGN_WholeBlood
HPSE2	10	0.02	2.30	0.02	GTEx_ColonTransverse
HRH3	20	0.03	−2.61	9.07×10^{-3}	DGN_WholeBlood
HSD17B12	11	0.57	−2.83	4.65×10^{-3}	DGN_WholeBlood
HSD17B12	11	0.58	−2.59	9.71×10^{-3}	GTEx_ColonTransverse
HSD17B13	4	0.14	−2.50	0.01	GTEx_ColonTransverse
HSD3B7	16	0.18	−2.95	3.16×10^{-3}	DGN_WholeBlood
HSF2	6	0.17	−2.58	1.00×10^{-2}	DGN_WholeBlood
HSF2	6	0.01	−2.42	0.02	GTEx_ColonTransverse
ICAM4	19	0.02	2.16	0.03	GTEx_ColonTransverse
ID1	20	0.02	−2.61	9.03×10^{-3}	DGN_WholeBlood
ID3	1	0.02	2.75	5.92×10^{-3}	DGN_WholeBlood
IER5L	9	0.04	−2.14	0.03	GTEx_ColonTransverse
IFI35	17	0.01	2.42	0.02	DGN_WholeBlood
IFLTD1	12	0.01	2.33	0.02	GTEx_ColonTransverse
IGF2BP3	7	0.03	−2.00	0.05	GTEx_ColonTransverse
IGFALS	16	0.22	−2.49	0.01	GTEx_ColonTransverse
IGHMBP2	11	0.24	−2.96	3.06×10^{-3}	GTEx_ColonTransverse

IGHMBP2	11	0.87	−2.52	0.01	DGN_WholeBlood
IGSF6	16	0.06	−2.33	0.02	DGN_WholeBlood
IKBKAP	9	0.08	−2.32	0.02	DGN_WholeBlood
IL10RA	11	0.08	−2.43	0.02	DGN_WholeBlood
IL17A	6	0.03	−2.48	0.01	GTEx_ColonTransverse
IL1RAP	3	0.03	2.66	7.89×10^{-3}	GTEx_ColonTransverse
IL23R	1	0.04	3.11	1.86×10^{-3}	DGN_WholeBlood
IL2RA	10	0.03	2.03	0.04	DGN_WholeBlood
IMP3	15	0.01	−2.75	5.98×10^{-3}	DGN_WholeBlood
INF2	14	0.08	−2.38	0.02	GTEx_ColonTransverse
IP6K1	3	0.01	−1.97	0.05	DGN_WholeBlood
IQCE	7	0.01	−2.34	0.02	GTEx_ColonTransverse
IQGAP1	15	0.13	2.05	0.04	DGN_WholeBlood
ITGA9	3	0.06	2.16	0.03	DGN_WholeBlood
ITGAD	16	0.23	−2.71	6.80×10^{-3}	DGN_WholeBlood
ITGAX	16	0.37	−2.41	0.02	DGN_WholeBlood
ITGB7	12	0.18	−2.13	0.03	DGN_WholeBlood
ITIH1	3	0.01	2.45	0.01	DGN_WholeBlood
ITIH2	10	0.06	−2.54	0.01	GTEx_ColonTransverse
JAG1	20	0.04	2.15	0.03	DGN_WholeBlood
JAM3	11	0.07	−3.15	1.62×10^{-3}	GTEx_ColonTransverse
JDP2	14	0.01	−2.40	0.02	DGN_WholeBlood
JKAMP	14	0.02	2.31	0.02	GTEx_ColonTransverse

KCNA3	1	0.16	2.26	0.02	DGN_WholeBlood
KCNAB3	17	0.09	2.04	0.04	DGN_WholeBlood
KCNIP3	2	0.02	2.01	0.04	GTEx_ColonTransverse
KCNJ15	21	0.29	2.79	5.31×10^{-3}	DGN_WholeBlood
KCNQ4	1	0.13	−2.30	0.02	DGN_WholeBlood
KCTD20	6	0.04	−2.46	0.01	DGN_WholeBlood
KDELC2	11	0.39	3.28	1.04×10^{-3}	DGN_WholeBlood
KDELC2	11	0.20	2.97	2.98×10^{-3}	GTEx_ColonTransverse
KIAA0391	14	0.48	−2.47	0.01	DGN_WholeBlood
KIAA0556	16	0.01	2.00	0.05	DGN_WholeBlood
KIAA1024	15	0.20	2.36	0.02	DGN_WholeBlood
KIAA1377	11	0.08	−2.16	0.03	DGN_WholeBlood
KIAA1430	4	0.05	2.43	0.02	GTEx_ColonTransverse
KIAA1432	9	0.02	2.19	0.03	GTEx_ColonTransverse
KIAA2018	3	0.01	−2.46	0.01	DGN_WholeBlood
KIAA2018	3	0.03	1.98	0.05	GTEx_ColonTransverse
KIF26B	1	0.01	−2.41	0.02	GTEx_ColonTransverse
KIF3C	2	0.03	2.50	0.01	DGN_WholeBlood
KLF5	13	0.01	−2.75	5.94×10^{-3}	GTEx_ColonTransverse
KLHDC8A	1	0.00	2.04	0.04	DGN_WholeBlood
KLHL20	1	0.05	−2.01	0.04	DGN_WholeBlood
KLHL22	22	0.02	−2.08	0.04	DGN_WholeBlood
KLK12	19	0.08	1.98	0.05	GTEx_ColonTransverse

KLK14	19	0.07	−2.39	0.02	DGN_WholeBlood
KRT13	17	0.02	−2.67	7.63×10^{-3}	DGN_WholeBlood
KRTAP5.1	11	0.03	−2.12	0.03	DGN_WholeBlood
LAMC1	1	0.23	4.67	2.97×10^{-6}	DGN_WholeBlood
LAMTOR2	1	0.07	−3.11	1.90×10^{-3}	GTEx_ColonTransverse
LARGE	22	0.07	−1.98	0.05	DGN_WholeBlood
LATS1	6	0.02	2.34	0.02	DGN_WholeBlood
LETMD1	12	0.01	−2.55	0.01	GTEx_ColonTransverse
LGALS3	14	0.03	2.04	0.04	DGN_WholeBlood
LGALS9	17	0.60	−2.45	0.01	DGN_WholeBlood
LGR4	11	0.07	−2.18	0.03	DGN_WholeBlood
LGR6	1	0.17	2.85	4.43×10^{-3}	DGN_WholeBlood
LILRA4	19	0.07	2.04	0.04	GTEx_ColonTransverse
LIMA1	12	0.01	−4.46	8.23×10^{-6}	GTEx_ColonTransverse
LIMA1	12	0.15	3.26	1.13×10^{-3}	DGN_WholeBlood
LIPM	10	0.01	−2.02	0.04	GTEx_ColonTransverse
LMCD1	3	0.19	−2.08	0.04	DGN_WholeBlood
LMO7	13	0.01	−3.21	1.31×10^{-3}	DGN_WholeBlood
LRCH1	13	0.09	−3.73	1.95×10^{-4}	DGN_WholeBlood
LRFN2	6	0.02	2.30	0.02	GTEx_ColonTransverse
LRIT3	4	0.10	−1.98	0.05	DGN_WholeBlood
LRRC28	15	0.06	−2.87	4.09×10^{-3}	GTEx_ColonTransverse
LRRC28	15	0.16	−2.64	8.37×10^{-3}	DGN_WholeBlood

LRRC29	16	0.13	−2.33	0.02	DGN_WholeBlood
LRRC3DN	21	0.15	1.98	0.05	GTEx_ColonTransverse
LRRC8B	1	0.15	−2.50	0.01	GTEx_ColonTransverse
LRRFIP1	2	0.08	2.22	0.03	DGN_WholeBlood
LRRFIP2	3	0.20	2.45	0.01	DGN_WholeBlood
LSM2	6	0.01	3.11	1.86×10^{-3}	GTEx_ColonTransverse
LSM2	6	0.03	3.01	2.64×10^{-3}	DGN_WholeBlood
LTA	6	0.10	−2.77	5.52×10^{-3}	DGN_WholeBlood
LZTR1	22	0.07	−2.31	0.02	GTEx_ColonTransverse
MAD1L1	7	0.22	−2.72	6.62×10^{-3}	DGN_WholeBlood
MAFF	22	0.06	−3.28	1.05×10^{-3}	DGN_WholeBlood
MAFK	7	0.05	1.99	0.05	DGN_WholeBlood
MAG	19	0.16	2.10	0.04	GTEx_ColonTransverse
MAGI2	7	0.02	2.44	0.01	DGN_WholeBlood
MAN1B1	9	0.23	−2.46	0.01	GTEx_ColonTransverse
MAP1A	15	0.03	−2.02	0.04	DGN_WholeBlood
MAP1LC3A	20	0.08	−2.94	3.33×10^{-3}	GTEx_ColonTransverse
MAP2K5	15	0.34	2.98	2.86×10^{-3}	DGN_WholeBlood
MAP3K12	12	0.06	2.86	4.29×10^{-3}	DGN_WholeBlood
MAP3K4	6	0.01	2.65	7.95×10^{-3}	GTEx_ColonTransverse
MAP7D1	1	0.22	−1.99	0.05	DGN_WholeBlood
MAPK11	22	0.03	2.46	0.01	GTEx_ColonTransverse
MAPK3	16	0.31	−1.98	0.05	DGN_WholeBlood

MARCH9	12	0.04	2.65	8.13×10^{-3}	DGN_WholeBlood
MAST2	1	0.03	−2.26	0.02	GTEx_ColonTransverse
MBD2	18	0.04	−2.05	0.04	DGN_WholeBlood
MC1R	16	0.44	2.22	0.03	DGN_WholeBlood
MCM6	2	0.14	2.18	0.03	DGN_WholeBlood
MCM7	7	0.01	−2.00	0.05	DGN_WholeBlood
MEPE	4	0.01	2.14	0.03	GTEx_ColonTransverse
MERTK	2	0.36	3.62	2.99×10^{-4}	DGN_WholeBlood
MESDC2	15	0.01	3.06	2.24×10^{-3}	GTEx_ColonTransverse
MESDC2	15	0.03	2.06	0.04	DGN_WholeBlood
METTL1	12	0.02	2.80	5.15×10^{-3}	DGN_WholeBlood
METTL14	4	0.02	−2.55	0.01	GTEx_ColonTransverse
METTL21A	2	0.04	2.21	0.03	GTEx_ColonTransverse
METTL21B	12	0.12	2.43	0.02	GTEx_ColonTransverse
METTL7A	12	0.04	−2.00	0.05	DGN_WholeBlood
MEX3A	1	0.02	−2.57	0.01	GTEx_ColonTransverse
MFSD6	2	0.34	2.01	0.04	DGN_WholeBlood
MICALCL	11	0.08	2.00	0.05	DGN_WholeBlood
MKKS	20	0.20	2.38	0.02	DGN_WholeBlood
MLEC	12	0.09	2.02	0.04	DGN_WholeBlood
MLH1	3	0.14	3.25	1.15×10^{-3}	DGN_WholeBlood
MLLT1	19	0.01	2.10	0.04	GTEx_ColonTransverse
MLLT11	1	0.09	−2.53	0.01	DGN_WholeBlood

MLLT4	6	0.18	−2.49	0.01	DGN_WholeBlood
MLLT4	6	0.08	−2.25	0.02	GTEx_ColonTransverse
MLYCD	16	0.03	−2.11	0.04	GTEx_ColonTransverse
MMEL1	1	0.32	−1.97	0.05	DGN_WholeBlood
MMP14	14	0.04	−2.22	0.03	DGN_WholeBlood
MMRN1	4	0.06	2.21	0.03	DGN_WholeBlood
MMRN2	10	0.20	2.22	0.03	DGN_WholeBlood
MON1B	16	0.02	2.46	0.01	GTEx_ColonTransverse
MRPL13	8	0.02	−2.03	0.04	DGN_WholeBlood
MRPL21	11	0.62	2.71	6.66×10^{-3}	DGN_WholeBlood
MRPL21	11	0.52	2.49	0.01	GTEx_ColonTransverse
MRPL32	7	0.05	−3.37	7.60×10^{-4}	DGN_WholeBlood
MRPL48	11	0.40	−1.98	0.05	DGN_WholeBlood
MRPL50	9	0.09	−2.37	0.02	DGN_WholeBlood
MRPL52	14	0.14	−2.17	0.03	DGN_WholeBlood
MRPS15	1	0.02	−2.43	0.01	DGN_WholeBlood
MRPS23	17	0.02	2.81	5.00×10^{-3}	DGN_WholeBlood
MS4A6A	11	0.02	2.11	0.03	GTEx_ColonTransverse
MTCH2	11	0.01	−2.05	0.04	GTEx_ColonTransverse
MTERFD1	8	0.02	−2.41	0.02	DGN_WholeBlood
MTF2	1	0.04	2.84	4.47×10^{-3}	DGN_WholeBlood
MTFP1	22	0.01	2.85	4.31×10^{-3}	GTEx_ColonTransverse
MTG2	20	0.04	−1.99	0.05	GTEx_ColonTransverse

MTUS1	8	0.62	2.27	0.02	DGN_WholeBlood
MXRA7	17	0.72	−2.69	7.16×10^{-3}	DGN_WholeBlood
MXRA7	17	0.26	−2.37	0.02	GTEx_ColonTransverse
MYCL1	1	0.02	−2.01	0.04	DGN_WholeBlood
MYH7B	20	0.05	−2.43	0.02	GTEx_ColonTransverse
MYO15A	17	0.08	3.26	1.13×10^{-3}	GTEx_ColonTransverse
MYO15A	17	0.09	2.19	0.03	DGN_WholeBlood
MYO1G	7	0.22	2.69	7.25×10^{-3}	DGN_WholeBlood
MYO5B	18	0.04	−2.63	8.45×10^{-3}	GTEx_ColonTransverse
MZF1	19	0.09	2.05	0.04	DGN_WholeBlood
N4BP2	4	0.02	1.99	0.05	DGN_WholeBlood
NAA20	20	0.02	2.07	0.04	DGN_WholeBlood
NAA40	11	0.05	2.31	0.02	DGN_WholeBlood
NAA50	3	0.01	−2.20	0.03	DGN_WholeBlood
NABP2	12	0.01	−2.24	0.03	GTEx_ColonTransverse
NAP1L4	11	0.15	2.16	0.03	DGN_WholeBlood
NAT14	19	0.09	2.66	7.71×10^{-3}	DGN_WholeBlood
NBEAL2	3	0.04	2.62	8.73×10^{-3}	DGN_WholeBlood
NBN	8	0.01	2.00	0.05	DGN_WholeBlood
NDRG1	8	0.05	−2.03	0.04	DGN_WholeBlood
NDUFA12	12	0.39	−2.02	0.04	DGN_WholeBlood
NDUFB2	7	0.04	−2.02	0.04	GTEx_ColonTransverse
NDUFB3	2	0.02	−2.68	7.37×10^{-3}	DGN_WholeBlood

NECAP2	1	0.01	2.34	0.02	GTEx_ColonTransverse
NEMF	14	0.00	−2.02	0.04	GTEx_ColonTransverse
NEO1	15	0.34	−3.11	1.90×10^{-3}	DGN_WholeBlood
NEU1	6	0.02	−2.02	0.04	DGN_WholeBlood
NFATC3	16	0.19	2.13	0.03	DGN_WholeBlood
NFE2L3	7	0.40	1.98	0.05	DGN_WholeBlood
NFKB1	4	0.05	2.51	0.01	DGN_WholeBlood
NFKBIL1	6	0.03	−2.14	0.03	DGN_WholeBlood
NFU1	2	0.06	−2.08	0.04	DGN_WholeBlood
NID2	14	0.08	2.03	0.04	GTEx_ColonTransverse
NIN	14	0.02	−2.84	4.53×10^{-3}	GTEx_ColonTransverse
NIT2	3	0.18	2.55	0.01	DGN_WholeBlood
NKAIN1	1	0.01	2.07	0.04	GTEx_ColonTransverse
NLRP7	19	0.01	−2.01	0.04	DGN_WholeBlood
NNT	5	0.03	2.13	0.03	GTEx_ColonTransverse
NOP2	12	0.01	−2.05	0.04	GTEx_ColonTransverse
NOS1	12	0.03	−2.59	9.63×10^{-3}	GTEx_ColonTransverse
NOX3	6	0.03	−2.08	0.04	GTEx_ColonTransverse
NPAS4	11	0.03	2.08	0.04	GTEx_ColonTransverse
NPIPA2	16	0.18	−2.63	8.54×10^{-3}	GTEx_ColonTransverse
NPIPA3	16	0.04	−2.23	0.03	GTEx_ColonTransverse
NPLOC4	17	0.03	−2.37	0.02	GTEx_ColonTransverse
NPTX1	17	0.07	−2.40	0.02	DGN_WholeBlood

NRN1	6	0.44	2.18	0.03	DGN_WholeBlood
NTN5	19	0.09	1.96	0.05	GTEx_ColonTransverse
NUDT13	10	0.31	2.52	0.01	GTEx_ColonTransverse
NUDT13	10	0.40	2.48	0.01	DGN_WholeBlood
NUFIP2	17	0.00	−2.14	0.03	DGN_WholeBlood
NUP133	1	0.02	2.08	0.04	GTEx_ColonTransverse
NUP133	1	0.08	2.05	0.04	DGN_WholeBlood
NUPL2	7	0.08	−2.63	8.46×10^{-3}	DGN_WholeBlood
NUPL2	7	0.15	−2.55	0.01	GTEx_ColonTransverse
NXPE2	11	0.01	−2.08	0.04	GTEx_ColonTransverse
NXPH3	17	0.01	−2.07	0.04	GTEx_ColonTransverse
NXT1	20	0.05	−2.06	0.04	GTEx_ColonTransverse
OCEL1	19	0.23	−2.71	6.78×10^{-3}	GTEx_ColonTransverse
OCEL1	19	0.31	−2.67	7.69×10^{-3}	DGN_WholeBlood
OCLN	5	0.08	2.13	0.03	GTEx_ColonTransverse
OLFM1	9	0.48	2.54	0.01	DGN_WholeBlood
OLFML1	11	0.01	2.02	0.04	GTEx_ColonTransverse
OMA1	1	0.01	−3.03	2.45×10^{-3}	GTEx_ColonTransverse
OMA1	1	0.12	−2.73	6.42×10^{-3}	DGN_WholeBlood
OR10A7	12	0.02	−2.00	0.05	GTEx_ColonTransverse
OR2AT4	11	0.02	3.96	7.59×10^{-5}	GTEx_ColonTransverse
OR2Z1	19	0.02	2.65	8.01×10^{-3}	GTEx_ColonTransverse
ORMDL2	12	0.02	2.14	0.03	GTEx_ColonTransverse

OSBP2	22	0.09	2.06	0.04	GTEx_ColonTransverse
OSBPL5	11	0.31	−2.10	0.04	DGN_WholeBlood
OSBPL6	2	0.20	2.10	0.04	DGN_WholeBlood
OTUB2	14	0.02	−2.36	0.02	DGN_WholeBlood
OTUD7B	1	0.01	2.82	4.82×10^{-3}	DGN_WholeBlood
OTX1	2	0.32	−2.41	0.02	DGN_WholeBlood
OXNAD1	3	0.05	−2.50	0.01	GTEx_ColonTransverse
OXSR1	3	0.05	−2.03	0.04	GTEx_ColonTransverse
PACSIN3	11	0.01	2.71	6.73×10^{-3}	DGN_WholeBlood
PAK6	15	0.01	1.98	0.05	DGN_WholeBlood
PALM	19	0.01	−2.02	0.04	GTEx_ColonTransverse
PALM	19	0.15	−1.98	0.05	DGN_WholeBlood
PAM16	16	0.19	−2.47	0.01	GTEx_ColonTransverse
PCNA	20	0.02	−2.63	8.43×10^{-3}	DGN_WholeBlood
PDCD5	19	0.19	2.35	0.02	DGN_WholeBlood
PDCD5	19	0.21	2.04	0.04	GTEx_ColonTransverse
PDE5A	4	0.01	−3.18	1.49×10^{-3}	DGN_WholeBlood
PDGFRB	5	0.01	2.85	4.44×10^{-3}	GTEx_ColonTransverse
PDK2	17	0.17	2.15	0.03	DGN_WholeBlood
PDLIM2	8	0.01	2.08	0.04	GTEx_ColonTransverse
PER2	2	0.02	1.96	0.05	GTEx_ColonTransverse
PFKM	12	0.03	2.47	0.01	DGN_WholeBlood
PGAP3	17	0.16	2.82	4.87×10^{-3}	GTEx_ColonTransverse

PGAP3	17	0.23	2.30	0.02	DGN_WholeBlood
PHACTR2	6	0.02	3.33	8.56×10^{-4}	DGN_WholeBlood
PHF20L1	8	0.04	−2.81	4.98×10^{-3}	DGN_WholeBlood
PHKG2	16	0.01	−2.15	0.03	DGN_WholeBlood
PICK1	22	0.51	2.78	5.36×10^{-3}	DGN_WholeBlood
PIK3R4	3	0.01	−2.30	0.02	DGN_WholeBlood
PILRB	7	0.61	2.22	0.03	DGN_WholeBlood
PINK1	1	0.04	2.56	0.01	DGN_WholeBlood
PIP4K2C	12	0.03	−2.30	0.02	DGN_WholeBlood
PLA2G6	22	0.03	−3.36	7.93×10^{-4}	GTEx_ColonTransverse
PLD6	17	0.25	2.92	3.50×10^{-3}	DGN_WholeBlood
PLEKHA1	10	0.15	2.01	0.04	DGN_WholeBlood
PLEKHG3	14	0.08	2.06	0.04	DGN_WholeBlood
PLEKHH1	14	0.06	2.22	0.03	GTEx_ColonTransverse
PLEKHM1	17	0.02	−2.17	0.03	DGN_WholeBlood
PLIN1	15	0.03	−2.23	0.03	GTEx_ColonTransverse
PLXNA1	3	0.02	2.30	0.02	DGN_WholeBlood
PNKD	2	0.28	2.74	6.07×10^{-3}	DGN_WholeBlood
PNPLA6	19	0.01	−1.97	0.05	DGN_WholeBlood
PNRC2	1	0.02	−2.28	0.02	GTEx_ColonTransverse
POLD2	7	0.01	2.31	0.02	DGN_WholeBlood
POLD3	11	0.01	−2.64	8.26×10^{-3}	DGN_WholeBlood
POLI	18	0.36	−2.43	0.02	GTEx_ColonTransverse

POLI	18	0.56	−2.42	0.02	DGN_WholeBlood
POLR1D	13	0.14	2.52	0.01	GTEx_ColonTransverse
POMGNT1	1	0.12	2.53	0.01	DGN_WholeBlood
POMT1	9	0.06	2.08	0.04	GTEx_ColonTransverse
POU5F1	6	0.18	−2.09	0.04	GTEx_ColonTransverse
POU5F1B	8	0.01	−6.20	5.62E−10	GTEx_ColonTransverse
POU5F1B	8	0.08	−2.50	0.01	DGN_WholeBlood
PPIL2	22	0.16	2.32	0.02	DGN_WholeBlood
PPM1M	3	0.01	−2.78	5.52×10^{-3}	GTEx_ColonTransverse
PPP1R1B	17	0.32	2.37	0.02	DGN_WholeBlood
PPP1R3B	8	0.01	−2.31	0.02	DGN_WholeBlood
PPP1R3E	14	0.01	2.13	0.03	GTEx_ColonTransverse
PPP2R3C	14	0.62	−2.09	0.04	DGN_WholeBlood
PPP6R2	22	0.02	2.23	0.03	DGN_WholeBlood
PPT1	1	0.78	2.51	0.01	DGN_WholeBlood
PRB2	12	0.01	1.97	0.05	GTEx_ColonTransverse
PRCD	17	0.01	1.97	0.05	GTEx_ColonTransverse
PREX1	20	0.03	2.70	6.91×10^{-3}	GTEx_ColonTransverse
PRG2	11	0.01	−2.60	9.19×10^{-3}	DGN_WholeBlood
PRKAR1B	7	0.08	1.97	0.05	DGN_WholeBlood
PRMT5	14	0.42	−2.63	8.44×10^{-3}	DGN_WholeBlood
PRMT5	14	0.02	2.41	0.02	GTEx_ColonTransverse
PROCR	20	0.03	2.68	7.36×10^{-3}	DGN_WholeBlood

PROKR1	2	0.01	2.02	0.04	GTEx_ColonTransverse
PRPF38A	1	0.05	−2.21	0.03	DGN_WholeBlood
PRSS50	3	0.01	−2.29	0.02	GTEx_ColonTransverse
PRUNE2	9	0.81	2.17	0.03	DGN_WholeBlood
PSMA6	14	0.01	−2.51	0.01	DGN_WholeBlood
PSMB11	14	0.46	2.41	0.02	DGN_WholeBlood
PSMF1	20	0.05	−2.01	0.04	DGN_WholeBlood
PSRC1	1	0.03	−2.32	0.02	GTEx_ColonTransverse
PSTPIP2	18	0.08	2.28	0.02	DGN_WholeBlood
PTK2	8	0.04	2.56	0.01	DGN_WholeBlood
PTPLAD2	9	0.09	2.16	0.03	DGN_WholeBlood
PTPRR	12	0.01	2.30	0.02	GTEx_ColonTransverse
PTRH1	9	0.06	2.03	0.04	DGN_WholeBlood
PVRIG	7	0.43	−2.19	0.03	DGN_WholeBlood
PXT1	6	0.01	2.04	0.04	DGN_WholeBlood
PYGL	14	0.26	−3.68	2.34×10^{-4}	GTEx_ColonTransverse
PYGL	14	0.07	2.81	4.93×10^{-3}	DGN_WholeBlood
RAB11FIP5	2	0.08	−2.37	0.02	DGN_WholeBlood
RAB27B	18	0.01	−2.22	0.03	DGN_WholeBlood
RAB32	6	0.01	−2.47	0.01	GTEx_ColonTransverse
RAB6A	11	0.12	2.55	0.01	DGN_WholeBlood
RABL2A	2	0.18	2.36	0.02	GTEx_ColonTransverse
RAC3	17	0.01	−2.70	6.94×10^{-3}	GTEx_ColonTransverse

RACGAP1	12	0.02	1.97	0.05	DGN_WholeBlood
RANBP10	16	0.02	−2.09	0.04	GTEx_ColonTransverse
RAPGEF2	4	0.16	2.02	0.04	DGN_WholeBlood
RARS	5	0.07	2.16	0.03	GTEx_ColonTransverse
RBAK	7	0.19	−2.57	0.01	DGN_WholeBlood
RBMS3	3	0.01	−2.16	0.03	DGN_WholeBlood
RBMXL2	11	0.01	−2.13	0.03	GTEx_ColonTransverse
RCVRN	17	0.11	−2.99	2.76×10^{-3}	GTEx_ColonTransverse
RCVRN	17	0.61	−2.90	3.69×10^{-3}	DGN_WholeBlood
RDX	11	0.16	2.84	4.57×10^{-3}	GTEx_ColonTransverse
RDX	11	0.04	2.32	0.02	DGN_WholeBlood
RECK	9	0.01	3.01	2.59×10^{-3}	GTEx_ColonTransverse
REPS1	6	0.16	−2.05	0.04	DGN_WholeBlood
RFFL	17	0.09	1.99	0.05	GTEx_ColonTransverse
RFPL2	22	0.19	2.86	4.23×10^{-3}	DGN_WholeBlood
RFX8	2	0.03	2.32	0.02	DGN_WholeBlood
RGL1	1	0.06	−2.96	3.08×10^{-3}	DGN_WholeBlood
RHBDF2	17	0.02	−2.18	0.03	DGN_WholeBlood
RHOH	4	0.03	2.67	7.51×10^{-3}	DGN_WholeBlood
RIOK1	6	0.14	−2.10	0.04	DGN_WholeBlood
RNASE7	14	0.06	−2.30	0.02	GTEx_ColonTransverse
RNF169	11	0.02	−2.40	0.02	GTEx_ColonTransverse
RNF213	17	0.02	2.18	0.03	GTEx_ColonTransverse

RNF31	14	0.03	2.40	0.02	GTEx_ColonTransverse
ROBO1	3	0.02	2.25	0.02	DGN_WholeBlood
ROS1	6	0.09	2.27	0.02	GTEx_ColonTransverse
RP11−1026M7.2	5	0.09	−2.51	0.01	GTEx_ColonTransverse
RP11−1105G2.3	12	0.01	−2.48	0.01	GTEx_ColonTransverse
RP11−231C14.4	16	0.02	2.08	0.04	GTEx_ColonTransverse
RP11−404L6.2	5	0.04	2.77	5.64×10^{-3}	GTEx_ColonTransverse
RPLP0	12	0.01	2.83	4.62×10^{-3}	DGN_WholeBlood
RPRM	2	0.34	2.02	0.04	DGN_WholeBlood
RPS10	6	0.03	2.03	0.04	DGN_WholeBlood
RPS9	19	0.30	−1.96	0.05	DGN_WholeBlood
RRAS2	11	0.12	2.21	0.03	DGN_WholeBlood
RREB1	6	0.01	2.32	0.02	DGN_WholeBlood
RRH	4	0.04	−2.07	0.04	GTEx_ColonTransverse
RSAD1	17	0.01	1.99	0.05	GTEx_ColonTransverse
RSL24D1	15	0.01	−3.60	3.16×10^{-4}	GTEx_ColonTransverse
RTEL1	20	0.01	−2.62	8.70×10^{-3}	GTEx_ColonTransverse
RWDD3	1	0.04	−2.22	0.03	GTEx_ColonTransverse
SAMD14	17	0.01	−2.30	0.02	DGN_WholeBlood
SCGB2B2	19	0.01	2.05	0.04	GTEx_ColonTransverse
SCN8A	12	0.09	2.13	0.03	DGN_WholeBlood
SCNN1A	12	0.01	−2.41	0.02	DGN_WholeBlood
SDCCAG3	9	0.04	−2.25	0.02	GTEx_ColonTransverse

SEC22C	3	0.01	2.67	7.60×10^{-3}	DGN_WholeBlood
SEC31B	10	0.55	2.27	0.02	DGN_WholeBlood
SEC31B	10	0.15	2.10	0.04	GTEx_ColonTransverse
SEMA4B	15	0.14	−2.67	7.65×10^{-3}	DGN_WholeBlood
SEPN1	1	0.01	2.06	0.04	DGN_WholeBlood
SERPINB10	18	0.36	2.23	0.03	DGN_WholeBlood
SERPINB6	6	0.04	2.18	0.03	GTEx_ColonTransverse
SETMAR	3	0.10	−2.31	0.02	GTEx_ColonTransverse
SFMBT1	3	0.23	2.53	0.01	GTEx_ColonTransverse
SGMS1	10	0.04	2.33	0.02	DGN_WholeBlood
SH2B3	12	0.04	−1.98	0.05	DGN_WholeBlood
SH2D4A	8	0.02	−2.39	0.02	DGN_WholeBlood
SH3RF3	2	0.26	2.14	0.03	DGN_WholeBlood
SHISA4	1	0.04	2.13	0.03	DGN_WholeBlood
SHOC2	10	0.02	2.55	0.01	GTEx_ColonTransverse
SIGIRR	11	0.02	2.22	0.03	GTEx_ColonTransverse
SIRT1	10	0.21	−2.71	6.80×10^{-3}	DGN_WholeBlood
SKOR1	15	0.08	2.91	3.65×10^{-3}	DGN_WholeBlood
SKOR1	15	0.09	2.59	9.56×10^{-3}	GTEx_ColonTransverse
SLC14A1	18	0.08	−2.17	0.03	GTEx_ColonTransverse
SLC14A2	18	0.01	2.23	0.03	GTEx_ColonTransverse
SLC19A3	2	0.15	−2.29	0.02	GTEx_ColonTransverse
SLC1A5	19	0.05	−2.13	0.03	DGN_WholeBlood

SLC20A1	2	0.30	−2.86	4.21×10^{-3}	DGN_WholeBlood
SLC22A31	16	0.14	3.82	1.32×10^{-4}	GTEx_ColonTransverse
SLC23A2	20	0.05	−2.45	0.01	DGN_WholeBlood
SLC25A17	22	0.04	2.10	0.04	DGN_WholeBlood
SLC25A26	3	0.28	−3.24	1.21×10^{-3}	DGN_WholeBlood
SLC25A26	3	0.15	−3.10	1.93×10^{-3}	GTEx_ColonTransverse
SLC25A28	10	0.01	−3.33	8.64×10^{-4}	DGN_WholeBlood
SLC25A4	4	0.07	2.70	6.87×10^{-3}	DGN_WholeBlood
SLC27A5	19	0.01	2.09	0.04	DGN_WholeBlood
SLC30A8	8	0.01	1.98	0.05	GTEx_ColonTransverse
SLC35B3	6	0.05	−2.82	4.79×10^{-3}	DGN_WholeBlood
SLC35E2	1	0.05	1.99	0.05	GTEx_ColonTransverse
SLC35F3	1	0.62	1.98	0.05	DGN_WholeBlood
SLC38A11	2	0.03	−2.13	0.03	DGN_WholeBlood
SLC46A1	17	0.01	2.16	0.03	DGN_WholeBlood
SLC4A5	2	0.03	−2.46	0.01	DGN_WholeBlood
SLC50A1	1	0.01	−2.43	0.02	GTEx_ColonTransverse
SLC5A8	12	0.01	2.21	0.03	GTEx_ColonTransverse
SLC6A1	3	0.01	2.48	0.01	GTEx_ColonTransverse
SLFN11	17	0.07	−2.07	0.04	DGN_WholeBlood
SLU7	5	0.05	−1.97	0.05	GTEx_ColonTransverse
SMAD1	4	0.03	−1.97	0.05	DGN_WholeBlood
SMAD6	15	0.04	3.67	2.45×10^{-4}	DGN_WholeBlood

SMAD7	18	0.03	2.10	0.04	DGN_WholeBlood
SMAD9	13	0.12	−2.42	0.02	GTEx_ColonTransverse
SMARCD1	12	0.01	2.74	6.05×10^{-3}	DGN_WholeBlood
SMC4	3	0.01	2.41	0.02	DGN_WholeBlood
SMCO4	11	0.17	2.01	0.04	GTEx_ColonTransverse
SND1	7	0.04	2.35	0.02	GTEx_ColonTransverse
SNRPA	19	0.02	−2.77	5.68×10^{-3}	DGN_WholeBlood
SNRPB2	20	0.01	−2.01	0.04	GTEx_ColonTransverse
SNRPE	1	0.02	−2.14	0.03	DGN_WholeBlood
SNUPN	15	0.37	2.24	0.03	DGN_WholeBlood
SNX1	15	0.19	−2.35	0.02	DGN_WholeBlood
SNX21	20	0.20	−2.20	0.03	DGN_WholeBlood
SNX27	1	0.03	2.19	0.03	GTEx_ColonTransverse
SNX33	15	0.06	−2.07	0.04	DGN_WholeBlood
SNX8	7	0.01	2.19	0.03	DGN_WholeBlood
SORCS2	4	0.08	−2.29	0.02	DGN_WholeBlood
SORCS3	10	0.03	2.87	4.08×10^{-3}	GTEx_ColonTransverse
SOX12	20	0.04	2.62	8.91×10^{-3}	DGN_WholeBlood
SPATS2L	2	0.05	2.65	8.09×10^{-3}	DGN_WholeBlood
SPEG	2	0.19	−2.29	0.02	DGN_WholeBlood
SPHK1	17	0.02	2.46	0.01	GTEx_ColonTransverse
SPHKAP	2	0.04	1.99	0.05	GTEx_ColonTransverse
SPNS3	17	0.02	−3.44	5.79×10^{-4}	DGN_WholeBlood

SPOCK1	5	0.03	2.57	0.01	GTEx_ColonTransverse
SPPL3	12	0.03	2.09	0.04	DGN_WholeBlood
SPRR2D	1	0.07	−2.64	8.18×10^{-3}	GTEx_ColonTransverse
SPTB	14	0.09	2.76	5.71×10^{-3}	DGN_WholeBlood
SRBD1	2	0.13	−2.28	0.02	DGN_WholeBlood
SRBD1	2	0.02	−2.27	0.02	GTEx_ColonTransverse
SS18L1	20	0.03	−2.80	5.15×10^{-3}	DGN_WholeBlood
SSBP4	19	0.08	2.77	5.56×10^{-3}	DGN_WholeBlood
SSU72	1	0.03	−2.01	0.04	GTEx_ColonTransverse
ST6GAL2	2	0.03	2.06	0.04	GTEx_ColonTransverse
STAP2	19	0.05	2.27	0.02	DGN_WholeBlood
STAT5A	17	0.01	−2.01	0.04	DGN_WholeBlood
STAT6	12	0.02	−2.62	8.68×10^{-3}	DGN_WholeBlood
STK39	2	0.01	−1.97	0.05	GTEx_ColonTransverse
STX1A	7	0.03	2.20	0.03	DGN_WholeBlood
SUCLG2	3	0.33	−2.11	0.03	DGN_WholeBlood
SUGP1	19	0.03	−2.00	0.05	DGN_WholeBlood
SULF2	20	0.38	−2.05	0.04	DGN_WholeBlood
SUPT7L	2	0.01	2.10	0.04	DGN_WholeBlood
SV2A	1	0.07	−2.26	0.02	GTEx_ColonTransverse
SYNDIG1	20	0.05	−3.21	1.33×10^{-3}	GTEx_ColonTransverse
SYNE4	19	0.02	2.43	0.01	GTEx_ColonTransverse
SYNPO2	4	0.19	2.24	0.02	DGN_WholeBlood

SYNPO2	4	0.02	−2.17	0.03	GTEx_ColonTransverse
SYT1	12	0.02	2.24	0.03	GTEx_ColonTransverse
TACSTD2	1	0.76	−2.08	0.04	DGN_WholeBlood
TAF6L	11	0.02	−2.90	3.77×10^{-3}	DGN_WholeBlood
TAS2R3	7	0.02	−2.37	0.02	GTEx_ColonTransverse
TATDN1	8	0.01	−2.04	0.04	DGN_WholeBlood
TBC1D2B	15	0.01	−2.05	0.04	GTEx_ColonTransverse
TBC1D30	12	0.19	2.79	5.31×10^{-3}	DGN_WholeBlood
TBC1D4	13	0.11	−2.13	0.03	GTEx_ColonTransverse
TBC1D9B	5	0.20	−2.50	0.01	GTEx_ColonTransverse
TBC1D9B	5	0.43	−1.97	0.05	DGN_WholeBlood
TBCA	5	0.09	2.71	6.75×10^{-3}	DGN_WholeBlood
TBX2	17	0.04	−2.71	6.77×10^{-3}	GTEx_ColonTransverse
TCEA2	20	0.04	−2.40	0.02	GTEx_ColonTransverse
TCTN2	12	0.17	−1.99	0.05	DGN_WholeBlood
TCTN3	10	0.20	−2.16	0.03	DGN_WholeBlood
TDRD6	6	0.32	−2.62	8.84×10^{-3}	GTEx_ColonTransverse
TDRD6	6	0.18	2.46	0.01	DGN_WholeBlood
TDRKH	1	0.12	2.26	0.02	GTEx_ColonTransverse
TDRKH	1	0.26	2.08	0.04	DGN_WholeBlood
TEDDM1	1	0.02	2.62	8.85×10^{-3}	DGN_WholeBlood
TELO2	16	0.14	2.04	0.04	GTEx_ColonTransverse
TEP1	14	0.01	−1.96	0.05	GTEx_ColonTransverse

TFAP4	16	0.01	2.20	0.03	DGN_WholeBlood
TFB2M	1	0.01	2.22	0.03	DGN_WholeBlood
TFCP2	12	0.04	2.50	0.01	DGN_WholeBlood
TFF2	21	0.01	1.99	0.05	GTEx_ColonTransverse
TFR2	7	0.01	1.96	0.05	GTEx_ColonTransverse
TG	8	0.39	−2.33	0.02	DGN_WholeBlood
TGFB1	19	0.00	−2.18	0.03	GTEx_ColonTransverse
TH1L	20	0.33	−1.98	0.05	DGN_WholeBlood
THADA	2	0.04	−2.03	0.04	DGN_WholeBlood
THBS1	15	0.22	−2.36	0.02	DGN_WholeBlood
THSD7B	2	0.11	2.35	0.02	GTEx_ColonTransverse
THUMPD1	16	0.22	2.17	0.03	DGN_WholeBlood
TIAM1	21	0.02	2.13	0.03	GTEx_ColonTransverse
TIGIT	3	0.02	2.92	3.46×10^{-3}	GTEx_ColonTransverse
TLR4	9	0.44	−2.22	0.03	DGN_WholeBlood
TM2D1	1	0.19	2.46	0.01	DGN_WholeBlood
TM7SF3	12	0.30	−2.19	0.03	DGN_WholeBlood
TM7SF3	12	0.01	−1.98	0.05	GTEx_ColonTransverse
TMA7	3	0.01	−1.97	0.05	GTEx_ColonTransverse
TMBIM1	2	0.05	2.25	0.02	DGN_WholeBlood
TMC8	17	0.09	−2.89	3.90×10^{-3}	GTEx_ColonTransverse
TMCC1	3	0.03	−2.11	0.04	DGN_WholeBlood
TMCO2	1	0.01	2.26	0.02	GTEx_ColonTransverse

TMEM110	3	0.15	−2.73	6.30×10^{-3}	DGN_WholeBlood
TMEM116	12	0.02	2.28	0.02	GTEx_ColonTransverse
TMEM119	12	0.31	2.16	0.03	DGN_WholeBlood
TMEM121	14	0.54	−3.26	1.12×10^{-3}	DGN_WholeBlood
TMEM133	11	0.02	−2.54	0.01	GTEx_ColonTransverse
TMEM154	4	0.01	−2.63	8.45×10^{-3}	DGN_WholeBlood
TMEM183A	1	0.04	2.29	0.02	DGN_WholeBlood
TMEM184B	22	0.03	2.36	0.02	GTEx_ColonTransverse
TMEM194B	2	0.09	−2.20	0.03	GTEx_ColonTransverse
TMEM230	20	0.25	−2.35	0.02	GTEx_ColonTransverse
TMEM44	3	0.20	−2.00	0.05	DGN_WholeBlood
TMEM62	15	0.01	2.68	7.39×10^{-3}	DGN_WholeBlood
TMEM81	1	0.10	2.15	0.03	DGN_WholeBlood
TMEM86B	19	0.16	2.37	0.02	DGN_WholeBlood
TMEM88	17	0.01	−2.06	0.04	DGN_WholeBlood
TMEM97	17	0.01	2.46	0.01	GTEx_ColonTransverse
TMPRSS3	21	0.02	−2.25	0.02	GTEx_ColonTransverse
TNF	6	0.09	−3.10	1.92×10^{-3}	DGN_WholeBlood
TNFSF13	17	0.06	−2.81	4.95×10^{-3}	DGN_WholeBlood
TNNC2	20	0.22	−2.40	0.02	DGN_WholeBlood
TOR1AIP1	1	0.03	1.97	0.05	DGN_WholeBlood
TOX	8	0.03	2.21	0.03	DGN_WholeBlood
TP53AIP1	11	0.02	−2.42	0.02	GTEx_ColonTransverse

TP53BP2	1	0.06	2.80	5.08×10^{-3}	DGN_WholeBlood
TP53RK	20	0.01	2.45	0.01	DGN_WholeBlood
TPD52	8	0.06	−2.52	0.01	GTEx_ColonTransverse
TPD52L2	20	0.05	−3.12	1.80×10^{-3}	DGN_WholeBlood
TPGS1	19	0.01	−2.20	0.03	GTEx_ColonTransverse
TRAF3IP2	6	0.12	−2.23	0.03	DGN_WholeBlood
TRAM1	8	0.02	−2.77	5.69×10^{-3}	DGN_WholeBlood
TRANK1	3	0.09	−2.91	3.61×10^{-3}	DGN_WholeBlood
TRIM24	7	0.13	−2.32	0.02	DGN_WholeBlood
TRIM39	6	0.01	2.71	6.67×10^{-3}	DGN_WholeBlood
TRIM4	7	0.51	−3.76	1.73×10^{-4}	GTEx_ColonTransverse
TRIM4	7	0.70	−3.63	2.81×10^{-4}	DGN_WholeBlood
TRIM5	11	0.09	2.10	0.04	DGN_WholeBlood
TRIP4	15	0.16	−2.54	0.01	DGN_WholeBlood
TRIP4	15	0.11	−2.22	0.03	GTEx_ColonTransverse
TRMT5	14	0.04	−1.99	0.05	DGN_WholeBlood
TRPV5	7	0.22	2.68	7.27×10^{-3}	DGN_WholeBlood
TRUB2	9	0.01	−2.75	5.91×10^{-3}	DGN_WholeBlood
TSEN34	19	0.17	2.32	0.02	DGN_WholeBlood
TSFM	12	0.32	−2.68	7.41×10^{-3}	DGN_WholeBlood
TSGA13	7	0.01	2.03	0.04	GTEx_ColonTransverse
TSPAN31	12	0.16	−2.06	0.04	DGN_WholeBlood
TSTD1	1	0.15	−2.21	0.03	DGN_WholeBlood

TTC18	10	0.11	2.37	0.02	DGN_WholeBlood
TTC18	10	0.07	2.22	0.03	GTEx_ColonTransverse
TTC23	15	0.42	−2.61	8.99×10^{-3}	DGN_WholeBlood
TTC24	1	0.29	2.26	0.02	DGN_WholeBlood
TTLL11	9	0.05	2.37	0.02	DGN_WholeBlood
TTLL6	17	0.04	2.71	6.72×10^{-3}	GTEx_ColonTransverse
TTPAL	20	0.10	−2.14	0.03	DGN_WholeBlood
TTYH1	19	0.01	−2.20	0.03	GTEx_ColonTransverse
TTYH1	19	0.01	2.17	0.03	DGN_WholeBlood
TUBA1B	12	0.07	−2.17	0.03	DGN_WholeBlood
TUBE1	6	0.06	3.00	2.72×10^{-3}	DGN_WholeBlood
TWIST2	2	0.07	2.26	0.02	DGN_WholeBlood
UAP1L1	9	0.15	−2.82	4.74×10^{-3}	GTEx_ColonTransverse
UBE2C	20	0.02	2.70	6.95×10^{-3}	DGN_WholeBlood
UBE2G2	21	0.06	−2.25	0.02	DGN_WholeBlood
UBE2M	19	0.01	1.97	0.05	DGN_WholeBlood
UBE2Z	17	0.32	2.70	6.90×10^{-3}	DGN_WholeBlood
UBR2	6	0.02	2.46	0.01	DGN_WholeBlood
UBXN10	1	0.01	2.22	0.03	GTEx_ColonTransverse
UBXN2A	2	0.04	−2.50	0.01	GTEx_ColonTransverse
UGT2B15	4	0.02	2.39	0.02	GTEx_ColonTransverse
UMODL1	21	0.03	2.10	0.04	GTEx_ColonTransverse
UNC5B	10	0.10	2.19	0.03	GTEx_ColonTransverse

URI1	19	0.01	−2.60	9.44×10^{-3}	GTEx_ColonTransverse
USF1	1	0.32	−2.15	0.03	DGN_WholeBlood
USF2	19	0.02	2.74	6.11×10^{-3}	DGN_WholeBlood
USP13	3	0.06	−2.98	2.89×10^{-3}	DGN_WholeBlood
USP15	12	0.14	−2.32	0.02	DGN_WholeBlood
USP3	15	0.10	2.02	0.04	DGN_WholeBlood
USP33	1	0.04	−2.10	0.04	DGN_WholeBlood
UTP11L	1	0.11	2.22	0.03	DGN_WholeBlood
UTP23	8	0.02	−2.74	6.13×10^{-3}	DGN_WholeBlood
VAMP3	1	0.03	−2.56	0.01	GTEx_ColonTransverse
VANGL1	1	0.15	−2.39	0.02	DGN_WholeBlood
VARS2	6	0.36	−2.35	0.02	DGN_WholeBlood
VGLL2	6	0.10	−2.22	0.03	GTEx_ColonTransverse
VIPR1	3	0.32	2.09	0.04	DGN_WholeBlood
VKORC1	16	0.03	−2.46	0.01	DGN_WholeBlood
VPS13D	1	0.01	2.18	0.03	DGN_WholeBlood
VPS16	20	0.21	2.22	0.03	DGN_WholeBlood
VPS26A	10	0.03	−2.27	0.02	DGN_WholeBlood
VPS33A	12	0.18	2.29	0.02	DGN_WholeBlood
VPS41	7	0.04	2.10	0.04	GTEx_ColonTransverse
WBSCR27	7	0.78	−2.97	2.94×10^{-3}	DGN_WholeBlood
WBSCR27	7	0.62	−2.96	3.09×10^{-3}	GTEx_ColonTransverse
WDR7	18	0.01	−2.18	0.03	GTEx_ColonTransverse

WDR82	3	0.01	−2.45	0.01	DGN_WholeBlood
WDYHV1	8	0.39	−3.08	2.08×10^{-3}	DGN_WholeBlood
WDYHV1	8	0.01	−2.05	0.04	GTEx_ColonTransverse
WFDC10B	20	0.30	−2.63	8.61×10^{-3}	GTEx_ColonTransverse
WFDC13	20	0.04	−2.80	5.05×10^{-3}	GTEx_ColonTransverse
WFDC3	20	0.41	2.55	0.01	GTEx_ColonTransverse
WFDC3	20	0.37	2.55	0.01	DGN_WholeBlood
WLS	1	0.36	−2.54	0.01	DGN_WholeBlood
WNT1	12	0.01	2.28	0.02	GTEx_ColonTransverse
WNT10A	2	0.02	2.45	0.01	GTEx_ColonTransverse
WWP1	8	0.03	1.97	0.05	GTEx_ColonTransverse
XRCC1	19	0.06	−2.28	0.02	DGN_WholeBlood
XRCC2	7	0.15	2.30	0.02	DGN_WholeBlood
XRCC2	7	0.04	2.04	0.04	GTEx_ColonTransverse
YIPF1	1	0.01	3.03	2.48×10^{-3}	DGN_WholeBlood
ZBED4	22	0.03	−2.09	0.04	GTEx_ColonTransverse
ZFAND3	6	0.06	−2.06	0.04	DGN_WholeBlood
ZFP106	15	0.01	−1.99	0.05	DGN_WholeBlood
ZFP36L1	14	0.04	1.99	0.05	DGN_WholeBlood
ZFP64	20	0.09	2.59	9.57×10^{-3}	DGN_WholeBlood
ZFPM1	16	0.13	−2.06	0.04	DGN_WholeBlood
ZFYVE19	15	0.03	3.07	2.11×10^{-3}	GTEx_ColonTransverse
ZFYVE19	15	0.35	2.97	3.00×10^{-3}	DGN_WholeBlood

ZNF17	19	0.01	2.27	0.02	DGN_WholeBlood
ZNF18	17	0.02	−2.82	4.77×10^{-3}	GTEx_ColonTransverse
ZNF253	19	0.01	−2.32	0.02	GTEx_ColonTransverse
ZNF276	16	0.06	−2.15	0.03	DGN_WholeBlood
ZNF282	7	0.28	−2.45	0.01	DGN_WholeBlood
ZNF300	5	0.04	−2.03	0.04	GTEx_ColonTransverse
ZNF318	6	0.07	−2.07	0.04	DGN_WholeBlood
ZNF350	19	0.01	−2.08	0.04	DGN_WholeBlood
ZNF408	11	0.02	2.54	0.01	GTEx_ColonTransverse
ZNF426	19	0.06	−3.36	7.79×10^{-4}	GTEx_ColonTransverse
ZNF467	7	0.31	2.24	0.03	DGN_WholeBlood
ZNF471	19	0.23	2.30	0.02	GTEx_ColonTransverse
ZNF498	7	0.05	−2.25	0.02	DGN_WholeBlood
ZNF506	19	0.18	2.95	3.14×10^{-3}	DGN_WholeBlood
ZNF512	2	0.01	−2.73	6.34×10^{-3}	DGN_WholeBlood
ZNF514	2	0.22	2.12	0.03	GTEx_ColonTransverse
ZNF558	19	0.07	−2.32	0.02	DGN_WholeBlood
ZNF558	19	0.09	−2.07	0.04	GTEx_ColonTransverse
ZNF629	16	0.01	−2.36	0.02	GTEx_ColonTransverse
ZNF638	2	0.02	−2.05	0.04	DGN_WholeBlood
ZNF653	19	0.01	−2.31	0.02	GTEx_ColonTransverse
ZNF668	16	0.26	−2.19	0.03	DGN_WholeBlood
ZNF682	19	0.08	2.70	6.83×10^{-3}	DGN_WholeBlood

ZNF682	19	0.16	2.32	0.02	GTEx_ColonTransverse
ZNF701	19	0.27	2.04	0.04	DGN_WholeBlood
ZNF740	12	0.08	2.62	8.67×10^{-3}	GTEx_ColonTransverse
ZNF740	12	0.06	2.29	0.02	DGN_WholeBlood
ZNF765	19	0.01	1.99	0.05	GTEx_ColonTransverse
ZNF786	7	0.11	−2.29	0.02	GTEx_ColonTransverse
ZNF786	7	0.31	−2.16	0.03	DGN_WholeBlood
ZSCAN16	6	0.03	2.02	0.04	DGN_WholeBlood
ZSCAN29	15	0.16	2.00	0.05	DGN_WholeBlood
ZSWIM1	20	0.01	−2.02	0.04	DGN_WholeBlood

Blue, novel with FDR=0.2; Red, SNP predictors in LD with known index or within 1Mb of known index
