# Supplementary figures and images for: Genetic variant predictors of gene expression provide new insight into risk of colorectal cancer
Source: Hum Genet. 2019 Feb 28;138(4):307–26. doi: 10.1007/s00439-019-01989-8 (PMC6483948; doi:10.1007/s00439-019-01989-8)

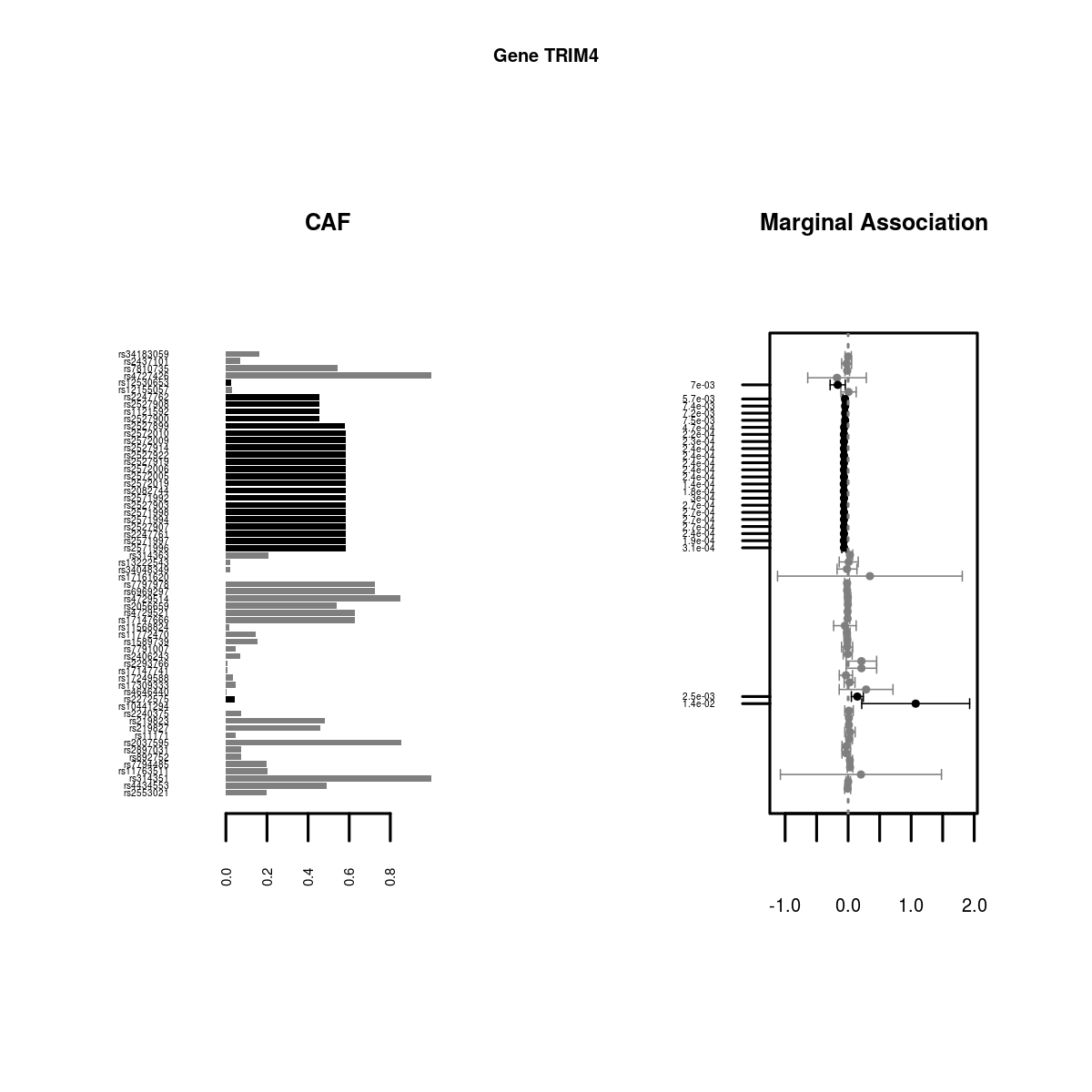

Supplement: Supplementary file 3 — Supplementary material 3 (TIFF 4218 KB) [file 439_2019_1989_MOESM3_ESM.tiff]

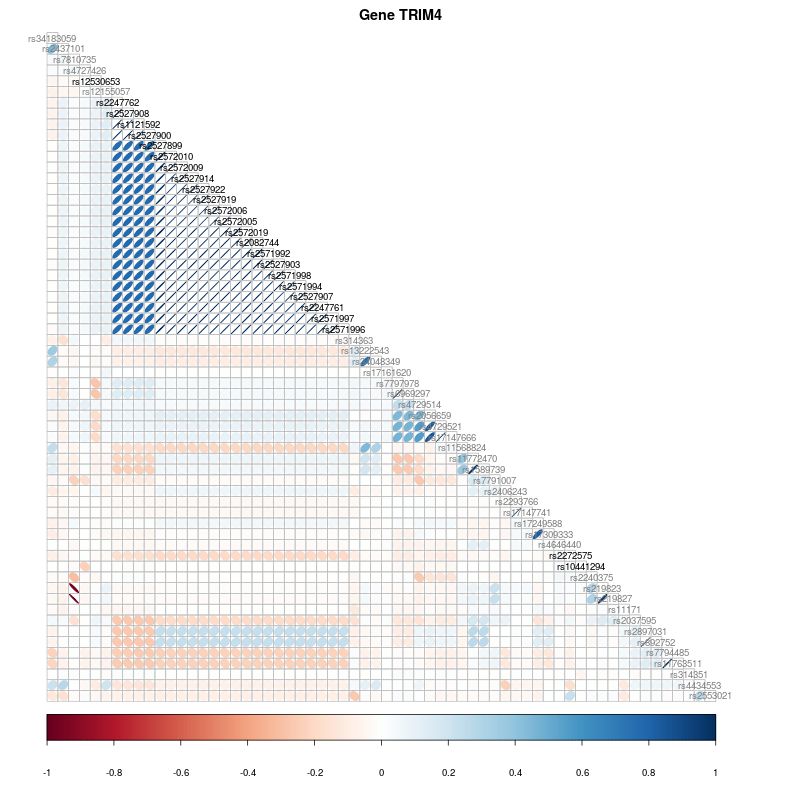

Supplement: Supplementary file 4 — Supplementary material 4 (TIFF 1875 KB) [file 439_2019_1989_MOESM4_ESM.tiff]

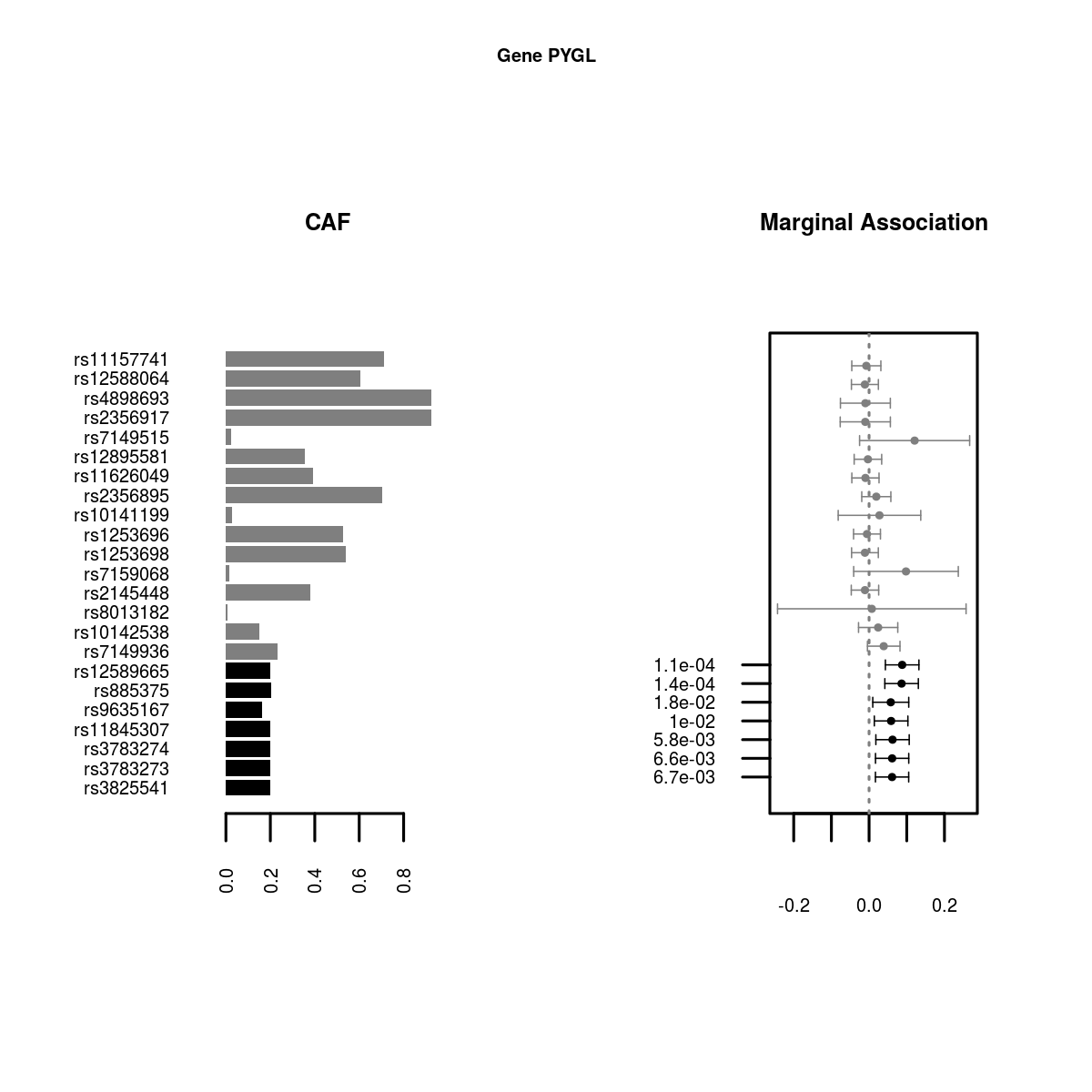

Supplement: Supplementary file 5 — Supplementary material 5 (TIFF 4218 KB) [file 439_2019_1989_MOESM5_ESM.tiff]

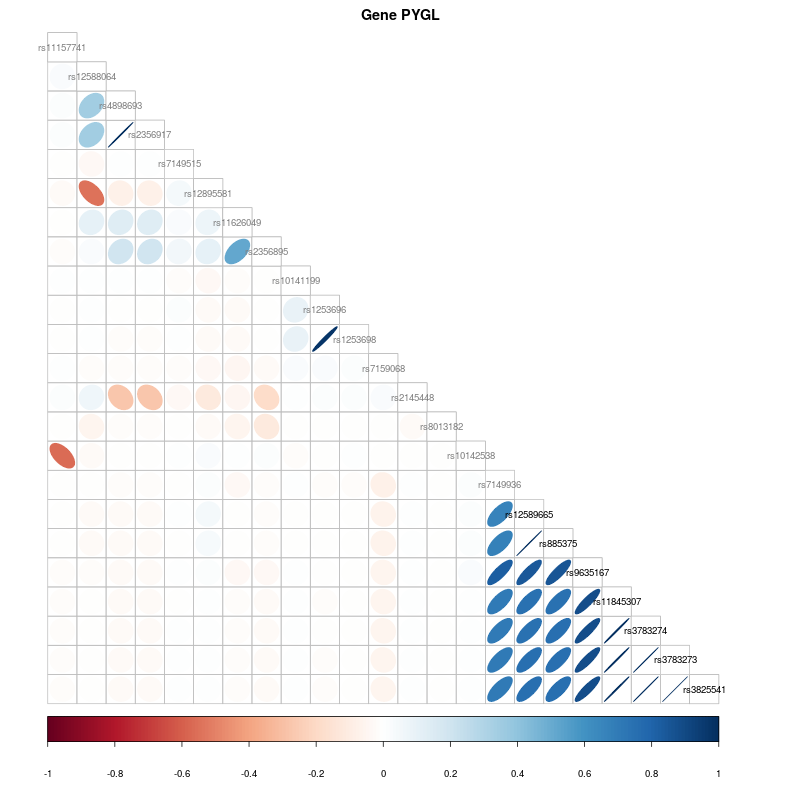

Supplement: Supplementary file 6 — Supplementary material 6 (TIFF 1875 KB) [file 439_2019_1989_MOESM6_ESM.tiff]
